# Supplementary material for: Identification of Novel Benzoxa-[2,1,3]-diazole Substituted Amino Acid Hydrazides as Potential Anti-Tubercular Agents
Source: Molecules. 2019 Feb 23;24(4):811. doi: 10.3390/molecules24040811 (PMC6412293; doi:10.3390/molecules24040811)
Supplement: Supplementary file 1 [file molecules-24-00811-s001.pdf]

Identification of novel benzoxa-[2,1,3]-diazole substituted amino acid hydrazides as potential anti-tubercular agents

Table S1: Initial screen of 99 compounds against a range of Gram-positive, Gram-negative and mycolata bacterial in a 96-well plate REMA assay at 128 µg/ml. Blue cells indicate no bacterial growth. Empty cells indicate bacterial growth.

		Gram-positive									Gram-negative							Mycolata			
Entry	Compound ID	*Bacillus cereus*	*Bacillus subtilis*	*Enterococcus faecalis*	*Enterococcus faecium*	*Listeria monocytogenes*	*Staphylococcus aureus*	*Streptococcus equi subsp. equi*	*Streptococcus agalactiae*	*Streptococcus pyogenes*	*Escherichia coli*	*Klebsiella pneumoniae*	*Proteus mirabilis*	*Pseudomonas aeroginosa*	*Salmonella enterica subsp. enterica*	*Serratia marcescens*	*Yersinia enterocolitica*	*Rhodococcus equi*	*Mycobacterium bovis BCG (pasteur)*	*Mycobacterium smegmatis* mc^{2}155	*Mycobacterium tuberculosis*
1	JDS-A001																				
2	JDS-A002							■										■	■		
3	JDS-A003	■	■								■	■	■			■	■	■	■	■	■
4	JDS-A004																		■		■
5	JDS-A005																		■		
6	JDS-A006																				
7	JDS-A007																				
8	JDS-A008																		■		
9	JDS-A009	■	■				■	■	■		■		■				■	■	■	■	■
10	JDS-A010																				
11	JDS-A011								■									■	■		
12	JDS-A012																		■		■
13	JDS-A013																		■		
14	JDS-A014																		■		
15	JDS-A015																	■	■		■

16	JDS-A016																				
17	JDS-A017																				
18	JDS-A018																				
19	JDS-A019																				
20	JDS-A020																				
21	JDS-A021																				
22	JDS-A022																				
23	JDS-A023																				
24	JDS-A024																				
25	JDS-A025																				
26	JDS-A026																				
27	JDS-A027																				
28	JDS-A028																				
29	JDS-A029																				
30	JDS-A030																				
31	JDS-A031																				
32	JDS-A032																				
33	JDS-A033																				
34	JDS-A034																				
35	JDS-A035																				
36	JDS-A036																				
37	JDS-A037																				
38	JDS-A038																				
39	JDS-A039																				
40	JDS-A040																				
41	JDS-A041																				
42	JDS-A042																				

43	JDS-A043																				
44	JDS-A044																				
45	JDS-A045																				
46	JDS-A046																				
47	JDS-A047																				
48	JDS-A048																				
49	JDS-A049																				
50	JDS-A050																				
51	JDS-A051																				
52	JDS-A052																				
53	JDS-A053																				
54	JDS-A054																				
55	JDS-A055																				
56	JDS-A056																				
57	JDS-A057																				
58	JDS-A058																				
59	JDS-A058-1																				
60	JDS-A059																				
61	JDS-A060																				
62	JDS-A061																				
63	JDS-A062																				
64	JDS-A063																				
65	JDS-A064																				
66	JDS-A065																				
67	JDS-A066																				
68	JDS-A067																				
69	JDS-A068																				

70	JDS-A069																				
71	JDS-A070																				
72	JDS-A071																				
73	JDS-A072																				
74	JDS-A073																				
75	JDS-A074																				
76	JDS-A075																				
77	JDS-A076																				
78	JDS-A077																				
79	JDS-A078																				
80	JDS-A079																				
81	JDS-A080																				
82	JDS-A081																				
83	JDS-A082																				
84	JDS-A083																				
85	JDS-A084																				
86	JDS-A085																				
87	JDS-A086																				
88	JDS-A087																				
89	JDS-A088																				
90	JDS-A089																				
91	JDS-A090																				
92	JDS-A091																				
93	JDS-A092																				
94	JDS-A093																				
95	JDS-A094																				
96	JDS-A095																				
97	JDS-A096																				

98	JDS-A097																				
99	JDS-A098																				
100	JDS-A099																				

Figure S1: Several of the amino acid hydrazides presented as a mixture of rotamers by NMR. To prove this, variable temperature ^{1}H-NMR of **14** in DMSO was undertaken. Raising the temperature from ambient (Spectrum 1) to 50 °C (Spectrum 2) demonstrated no discernible difference in signals however, a further increase to 100 °C saw coalescence of the signals (Spectrum 3). Furthermore, returning the sample to room temperature allowed for the rotamers to regress back to their original values and a spectrum identical to spectrum 1.

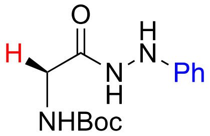

14

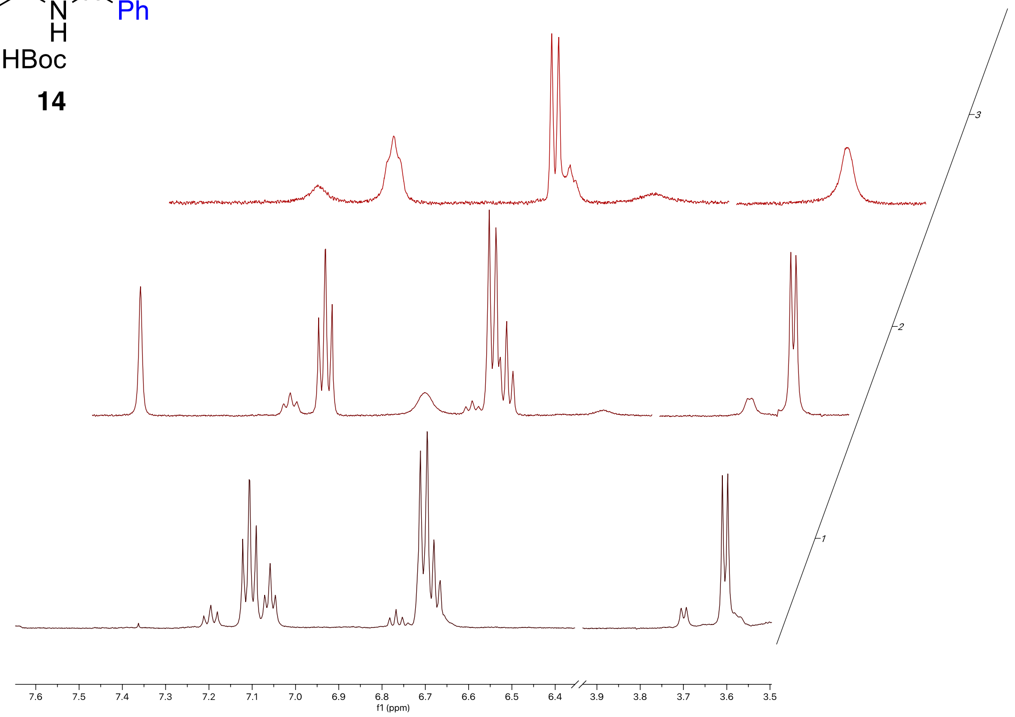

VT ^{1}H-NMR of 12 at 1) room temperature; 2) 50 ℃; 3) 100 ℃; Blank space in the spectra have been removed for clarity and increased coverage of the spectrum.

Table S2: Antibacterial activity against Gram-positive, -negative and mycolata bacteria and Mammalian Cell Toxicity of amino acid hydrazides and benzoxa-[2,1,3]-diazole amino acid hydrazides, expressed as MIC (µM) or percentage relative to control (100 %). - = No activity from the REMA assay at 128 µg/ml

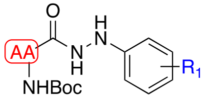

13a - 22a

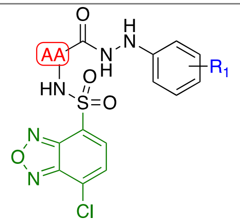

9, 10, 14b - 22b

MIC (µM)

Amino Acid (AA)	Hydrazine (R_1)	**Compound**	Yield	Rotamer ratio	***M. tuberculosis* mc²7000** µM	**Mammalian Cell Toxicity**	***S. aureus* (MRSA)**	***M. luteus***	***B. subtilis***	***B. cereus***	***E. faecalis***	***E. faecium***	***S. agalactiae***	***S. pyogenes***	***Y. enterocolitica***	***P. aeruginosa***	***S. marcescens***	***E. coli***	***M. smegmatis***
Gly	4-CF_3	**13a**	47 %	-	**12.01**	**0 %**	-	-	-	-	-	-	-	-	-	-	-	-	-
		**9**	7 %	7:1	**31.13**	**>90 %**	-	-	-	-	-	-	-	-	-	-	-	-	-
Phg	4-CF_3	**14a**	95 %	-	**117.24**	**0 %**	-	-	-	-	-	-	-	-	-	-	-	-	-
		**14b**	63 %	-	**60.85**	**>90 %**	-	-	-	-	-	-	-	-	-	-	-	-	-
Pro	4-CF_3	**15a**	85 %	-	**214.26**	**0 %**	-	-	-	-	-	-	-	-	-	-	-	-	-
		**15b**	85 %		**16.33**	**>90 %**	-	-	-	-	-	-	-	-	-	-	-	-	-
Phe	4-CF_3	**16a**	83 %	-	**151.14**	**<10 %**	-	-	-	-	-	-	-	-	-	-	-	-	-
		**16b**	85 %	-	**59.27**	**>90 %**	-	-	-	-	-	-	-	-	-	-	-	-	-
Ala	4-CF_3	**17a**	93 %	-	**46.06**	**0 %**	-	-	-	-	-	-	-	-	-	-	-	-	-

		**17b**	60 %	-	**17.25**	**> 90 %**	-	-	-	-	-	-	-	-	-	-	-	-	-
Gly	H	**18a**	76 %	5:1	**90.46**	**57 %**	-	-	-	-	-	-	-	-	-	-	-	-	-
		**10**	60 %	4:1	**36.67**	**0 %**	-	-	-	-	-	-	-	-	-	-	-	-	20.95
Gly	4-F	**19a**	78 %	4:1	**112.95**	**59 %**	-	-	-	-	-	-	-	-	-	-	-	-	-
		**19b**	57 %	4:1	**20.00**	**60 %**	-	-	-	-	-	-	-	-	-	-	-	-	-
Gly	4-Cl	**20a**	96 %	4:1	**106.75**	**43 %**	-	-	-	-	-	-	-	-	-	-	-	-	1.67
		**20b**	43 %	6:1	**19.22**	**80 %**	-	-	-	-	-	-	-	-	-	-	-	-	-
Gly	3-Cl	**21a**	80 %	4:1	**213.50**	**55 %**	-	-	-	-	-	-	-	-	-	-	-	-	3.34
		**21b**	22 %	5:1	**19.22**	**45 %**	-	-	-	-	-	-	-	-	-	-	-	-	4.81
Gly	2-Cl	**22a**	95 %	3:1	**106.75**	**<20 %**	-	-	-	-	-	-	-	-	-	-	-	-	26.69
		**22b**	29 %	4:1	**19.22**	**70 %**	-	-	-	-	-	-	-	-	-	-	-	-	-

Table S3: Bacterial strains and growth media used in the REMA assays.

**Test Organisms**	**Strain**	**Growth Media (Broth)**
Bacillus cereus	7464	Brain Heart Infusion
Bacillus subtilis	9372	Brain Heart Infusion
Enterococcus faecalis	775	Brain Heart Infusion
Enterococcus faecium	7171	Brain Heart Infusion
Escherichia coli	K12	Nutrient
Klebsiella pneumonae	9282	Brain Heart Infusion
Listeria monocytogenes	NCTC 10357	Nutrient
Micrococcus luteus	ATCC10786	Nutrient
Mycobacterium bovis BCG	Pasteur	Middlebrook 7H9
Mycobacterium smegmatis	mc^{2}155	Middlebrook 7H9
Mycobacterium tuberculosis	mc^{2}7000 (RD1 *panCD*)	Middlebrook 7H9
Proteus mirabilis	NCTC 11938	Nutrient
Pseudomonas aeruginosa	19880	Nutrient
Rhodococcus equi	NCTC1621	Nutrient
Salmonella enterica subsp. enterica	NCTC 6754	Nutrient
Serratia marcescens	NCTC 10211	Nutrient
Staphylococcus aureus	DSM110	Nutrient
Staphylococcus aureus	MRSA	Nutrient
Streptococcus agalactiae	NCTC8181	Todd Hewitt
Streptococcus equi	4044	Todd Hewitt
Streptococcus equi	4047	Todd Hewitt
Streptococcus pyogenes	20565	Todd Hewitt
Yersinia enterocolitica	NCTC11176	Nutrient

^{1}H NMR, ^{13}C NMR for all compounds

Compound 1 is commercially available (CAS: 20138-79-8)

Compound 2, 3, 4 are all patented compounds (WO2009034396A2)

Compound 5

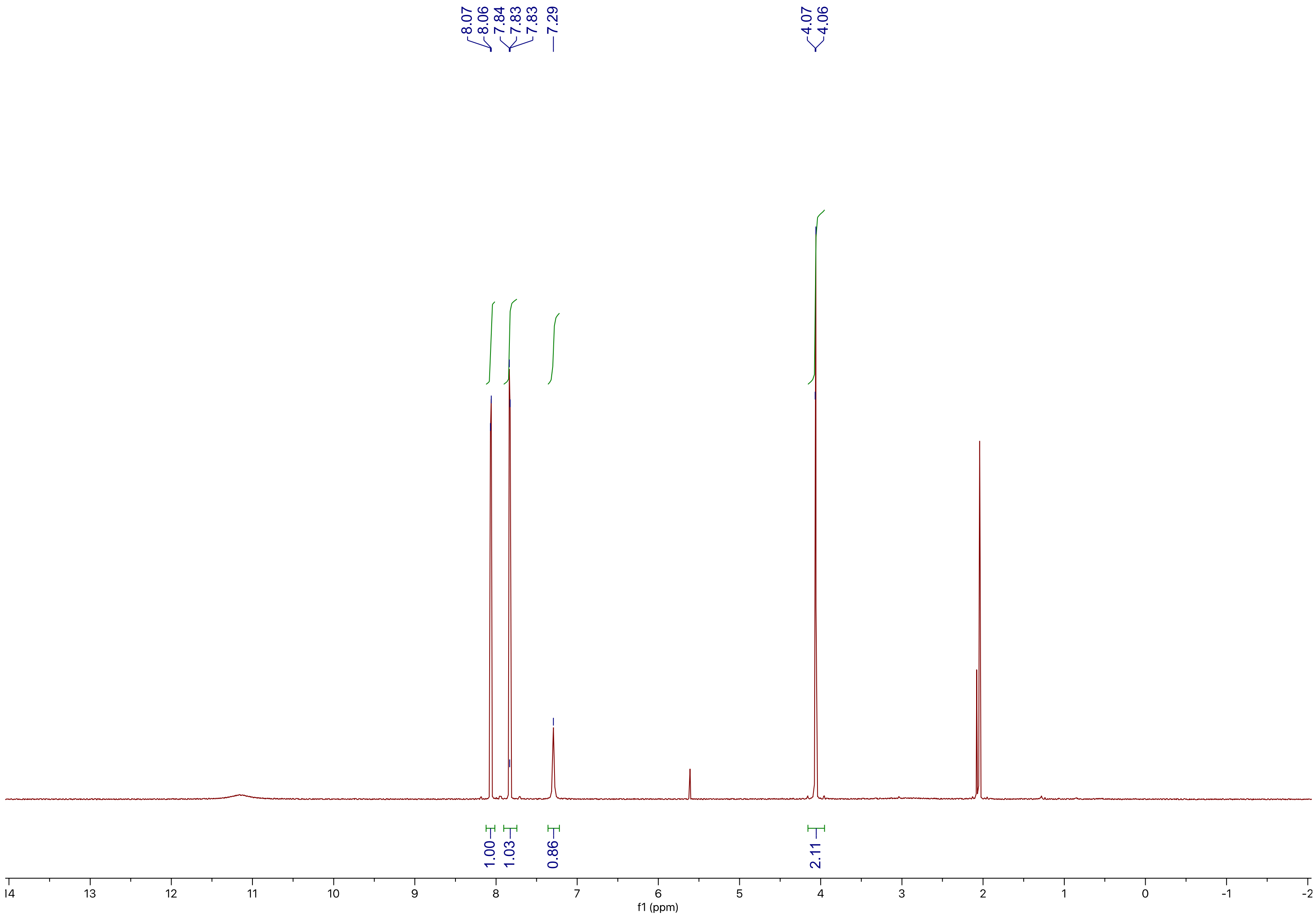
8.07
8.06
7.84
7.83
7.83
7.29
4.07
4.06
1.00
1.03
0.86
2.11
14
13
12
11
10
9
8
7
6
5
4
3
2
1
0
-1
-2
f1 (ppm)

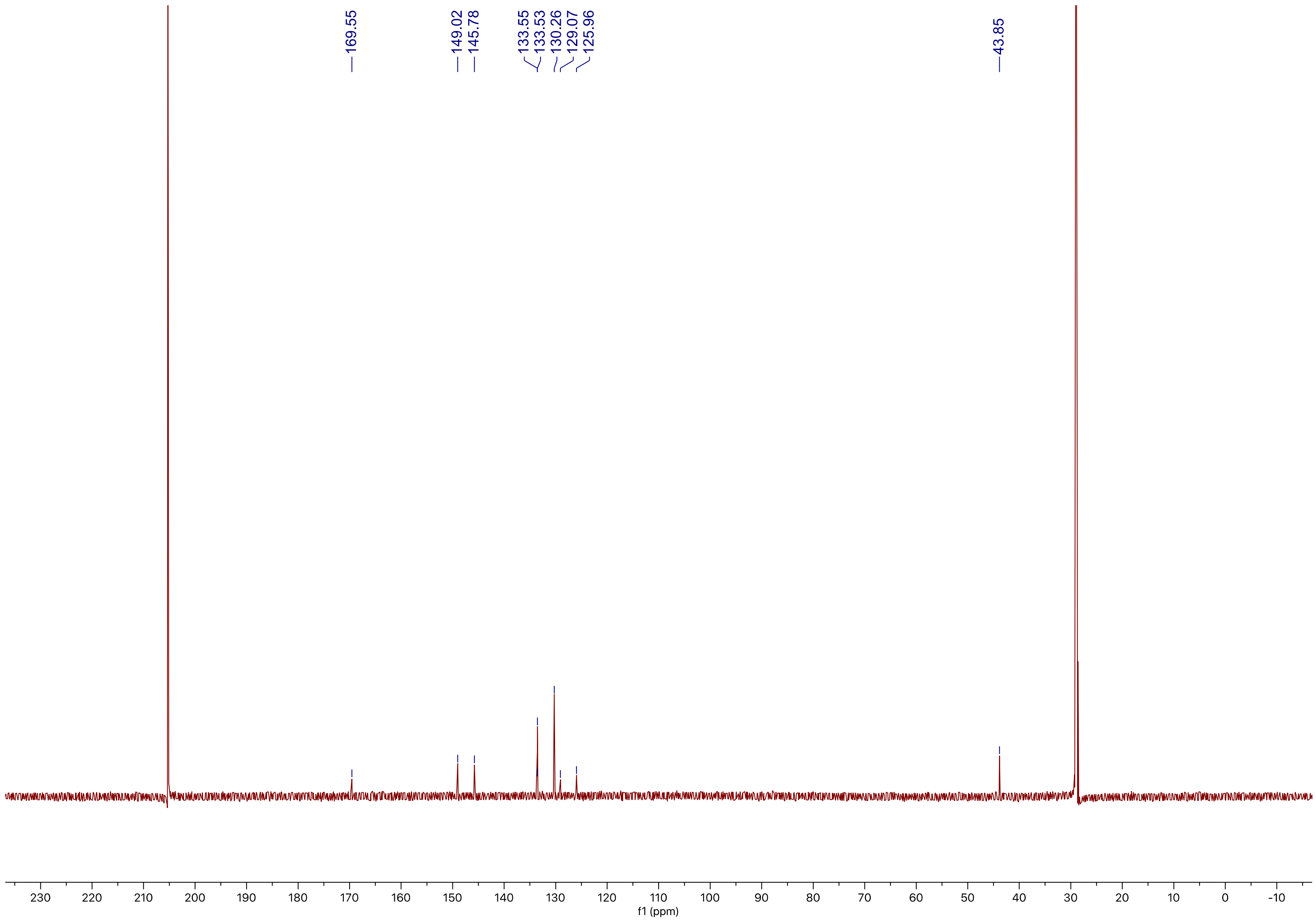
169.55
149.02
145.78
133.55
133.53
130.26
129.07
125.96
43.85
230 220 210 200 190 180 170 160 150 140 130 120 110 100 90 80 70 60 50 40 30 20 10 0 -10
f1 (ppm)

Compound 6

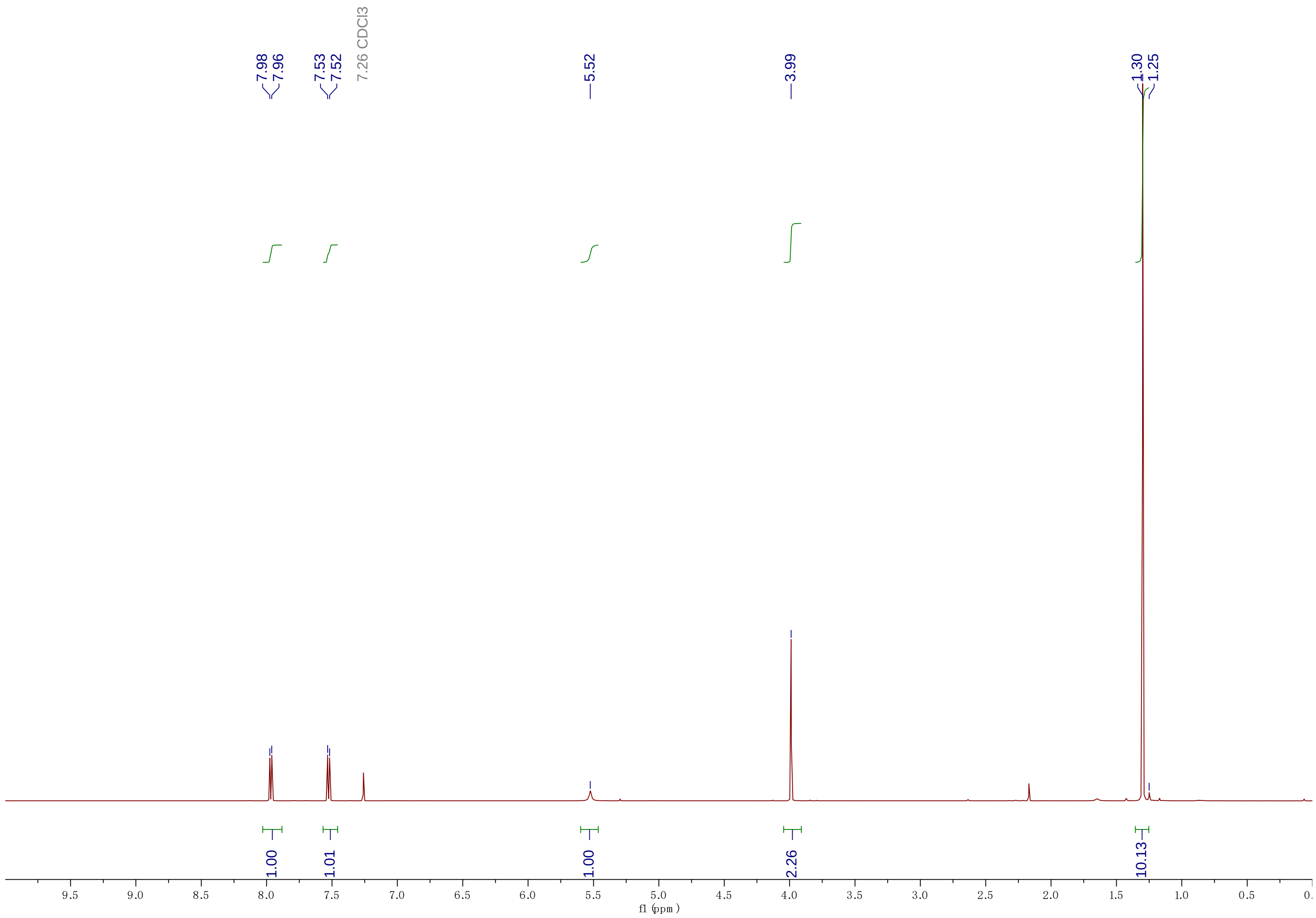
7.98
7.96
7.53
7.52
7.26 CDCl3
5.52
3.99
1.30
1.25
1.00
1.01
1.00
2.26
10.13
9.5 9.0 8.5 8.0 7.5 7.0 6.5 6.0 5.5 5.0 4.5 4.0 3.5 3.0 2.5 2.0 1.5 1.0 0.5
f1 (ppm)

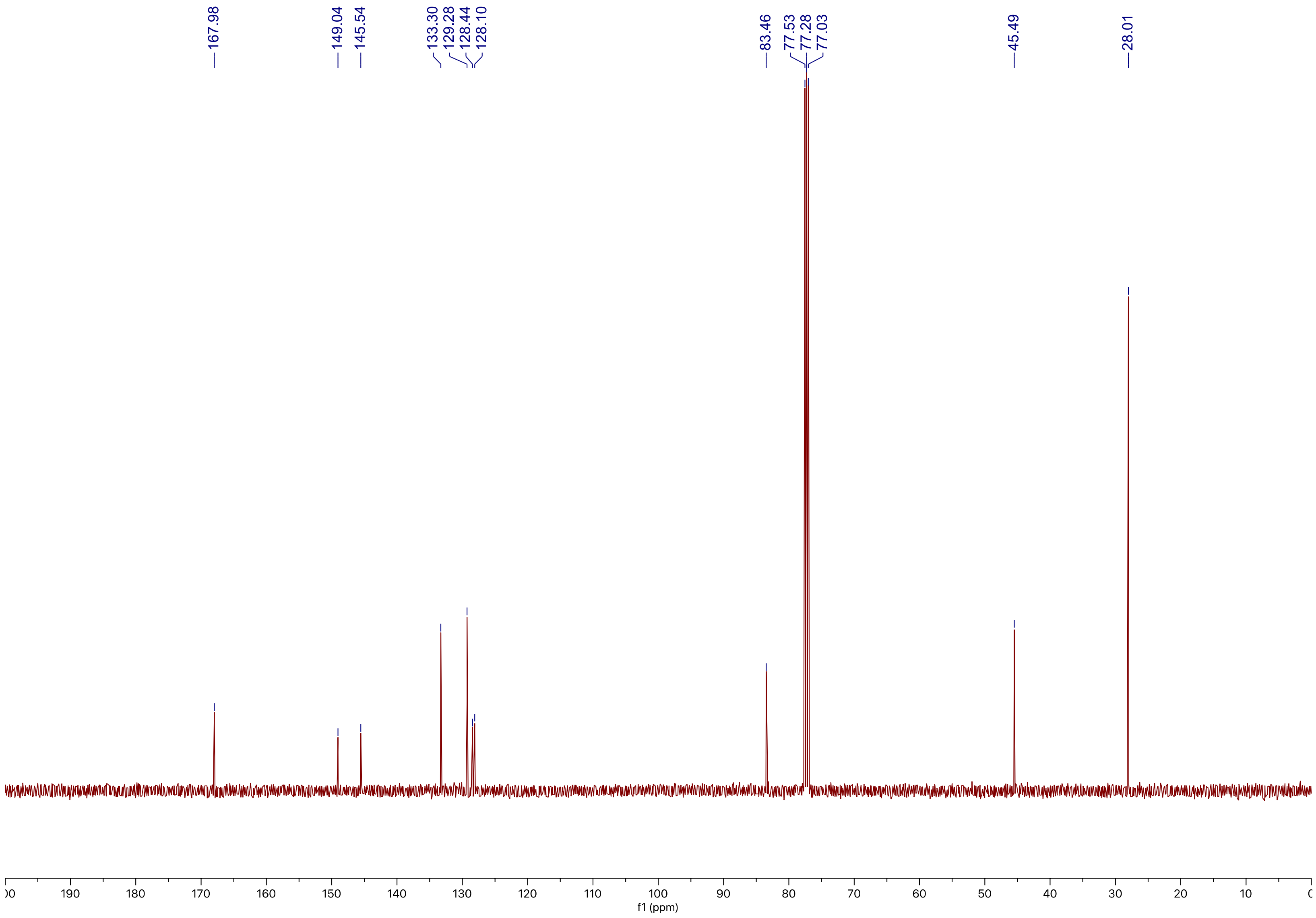
167.98
149.04
145.54
133.30
129.28
128.44
128.10
83.46
77.53
77.28
77.03
45.49
28.01
f1 (ppm)

Compound 7

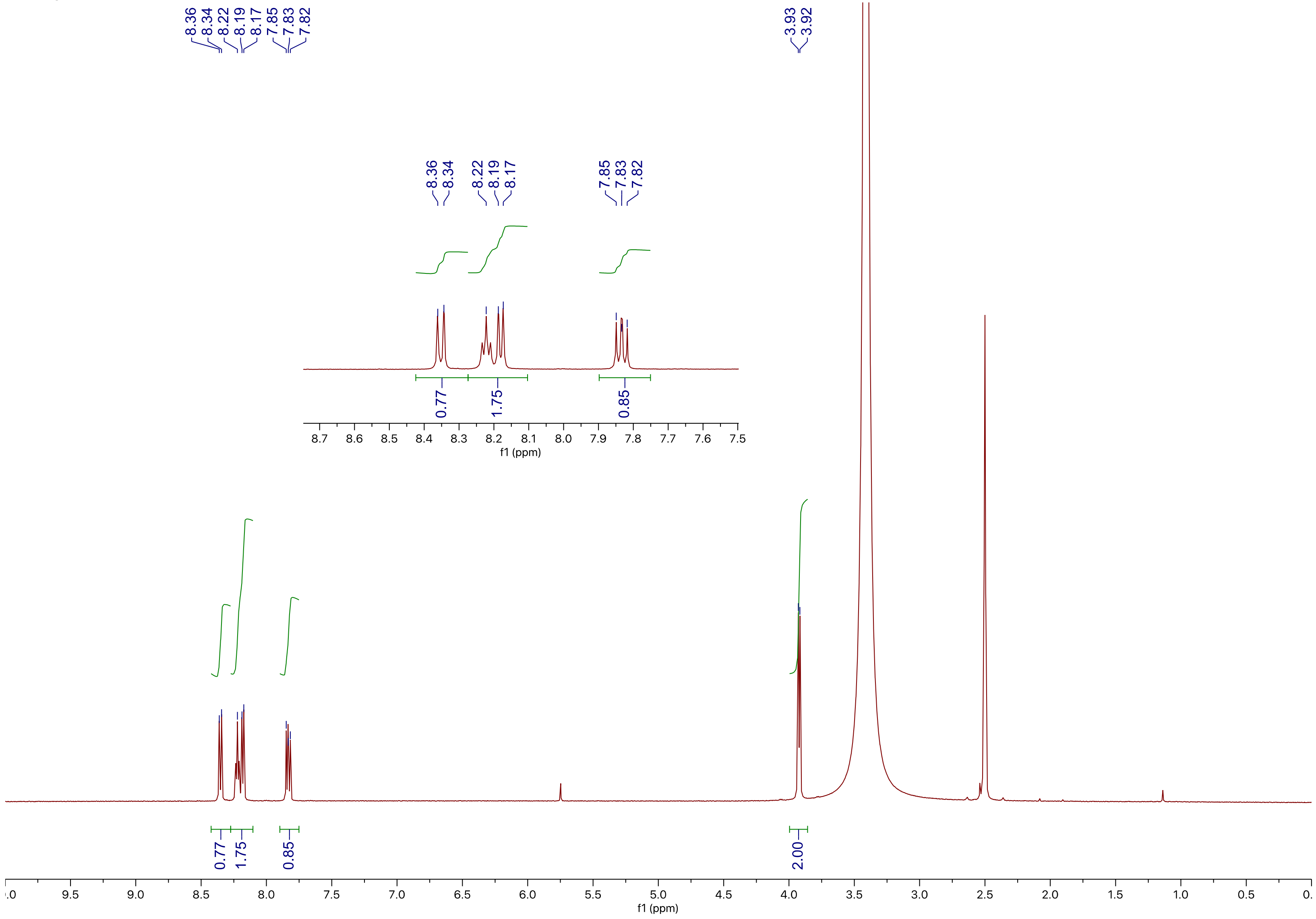

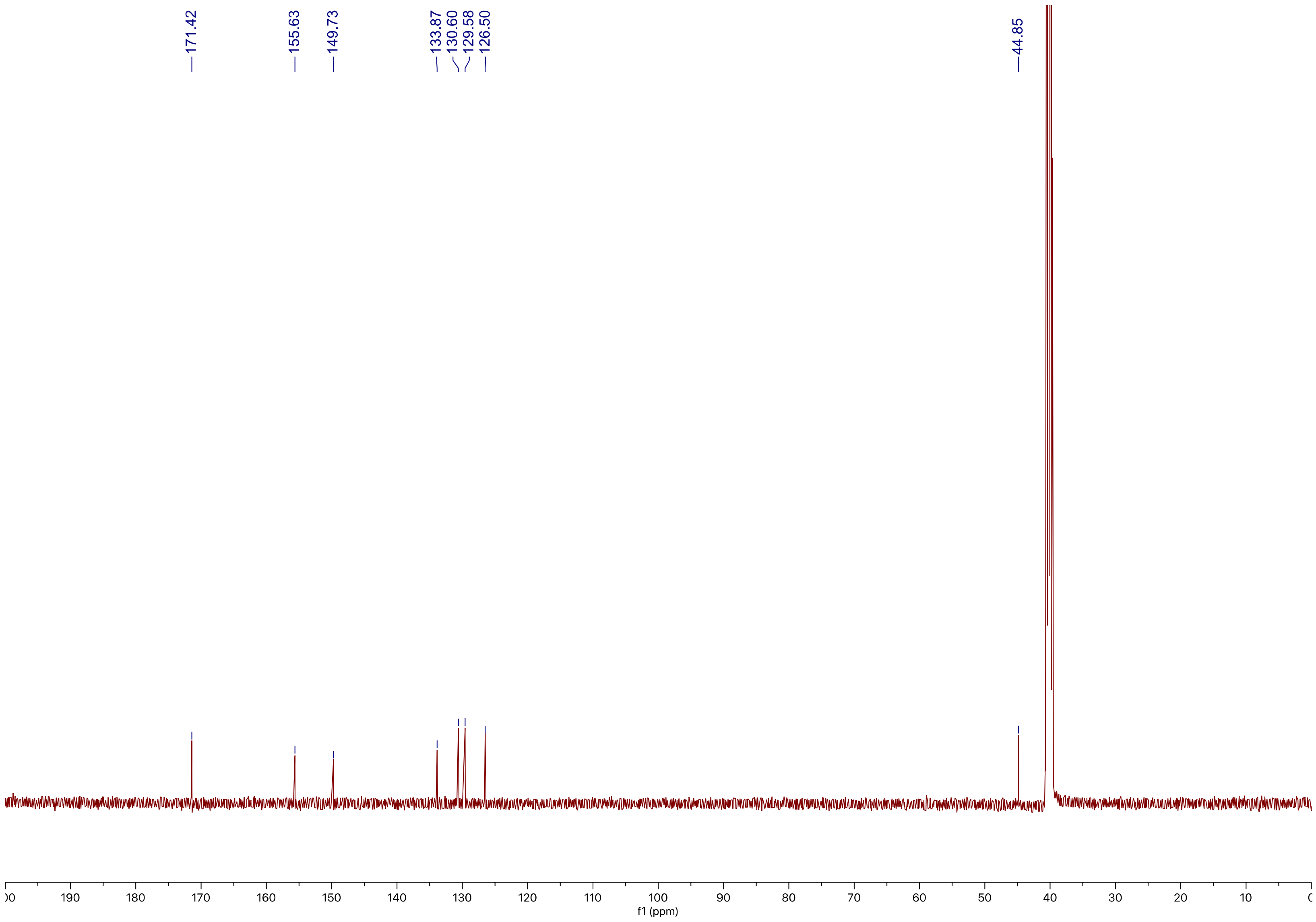
171.42
155.63
149.73
133.87
130.60
129.58
126.50
44.85
f1 (ppm)

Compound 8

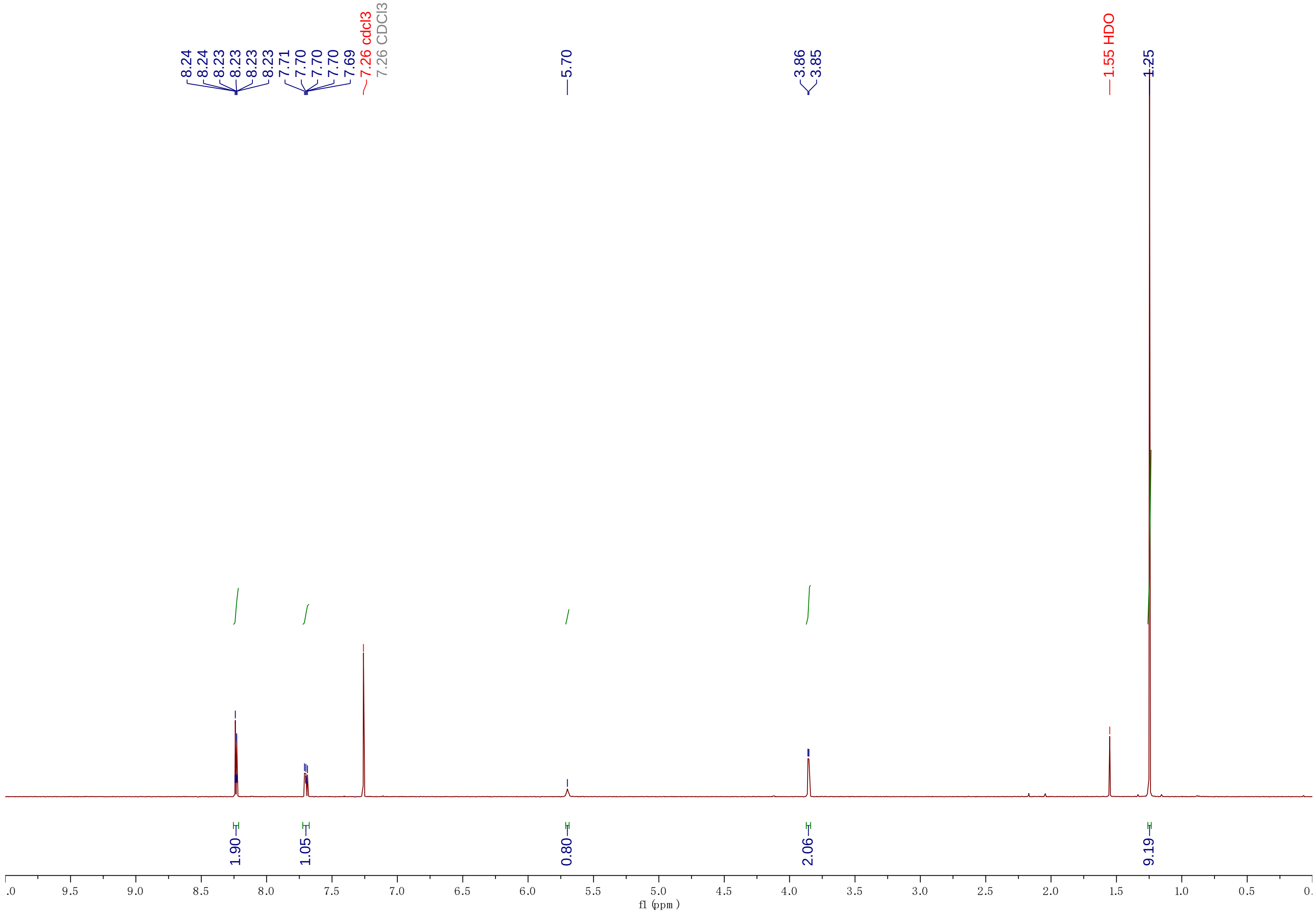
8.24
8.24
8.23
8.23
8.23
8.23
7.71
7.70
7.70
7.70
7.69
7.26 cdcl3
7.26 CDCl3
5.70
3.86
3.85
1.55 HDO
1.25
1.90
1.05
0.80
2.06
9.19
f1 (ppm)

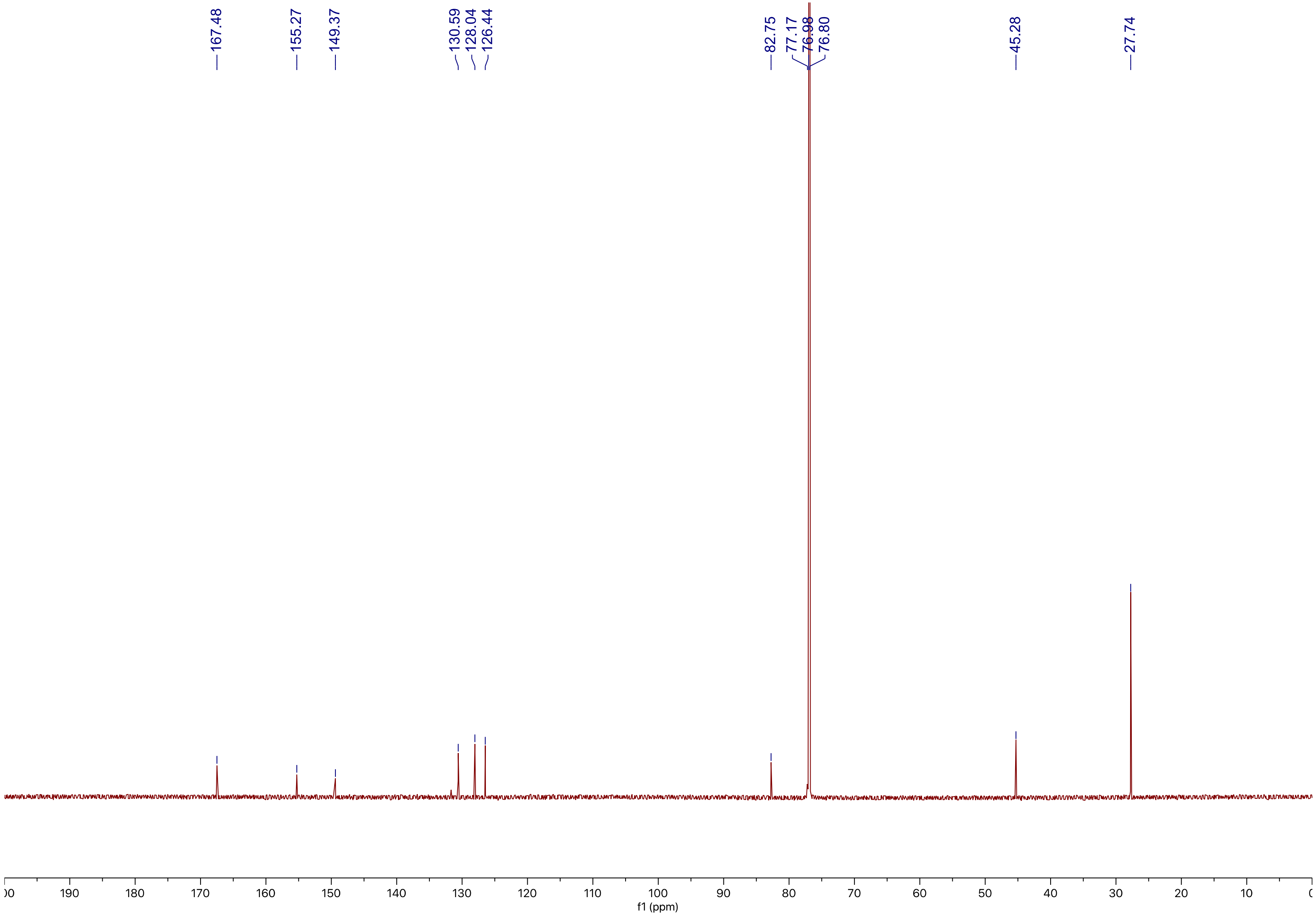
167.48
155.27
149.37
130.59
128.04
126.44
82.75
77.17
76.98
76.80
45.28
27.74
190 180 170 160 150 140 130 120 110 100 90 80 70 60 50 40 30 20 10
f1 (ppm)

Compound 9

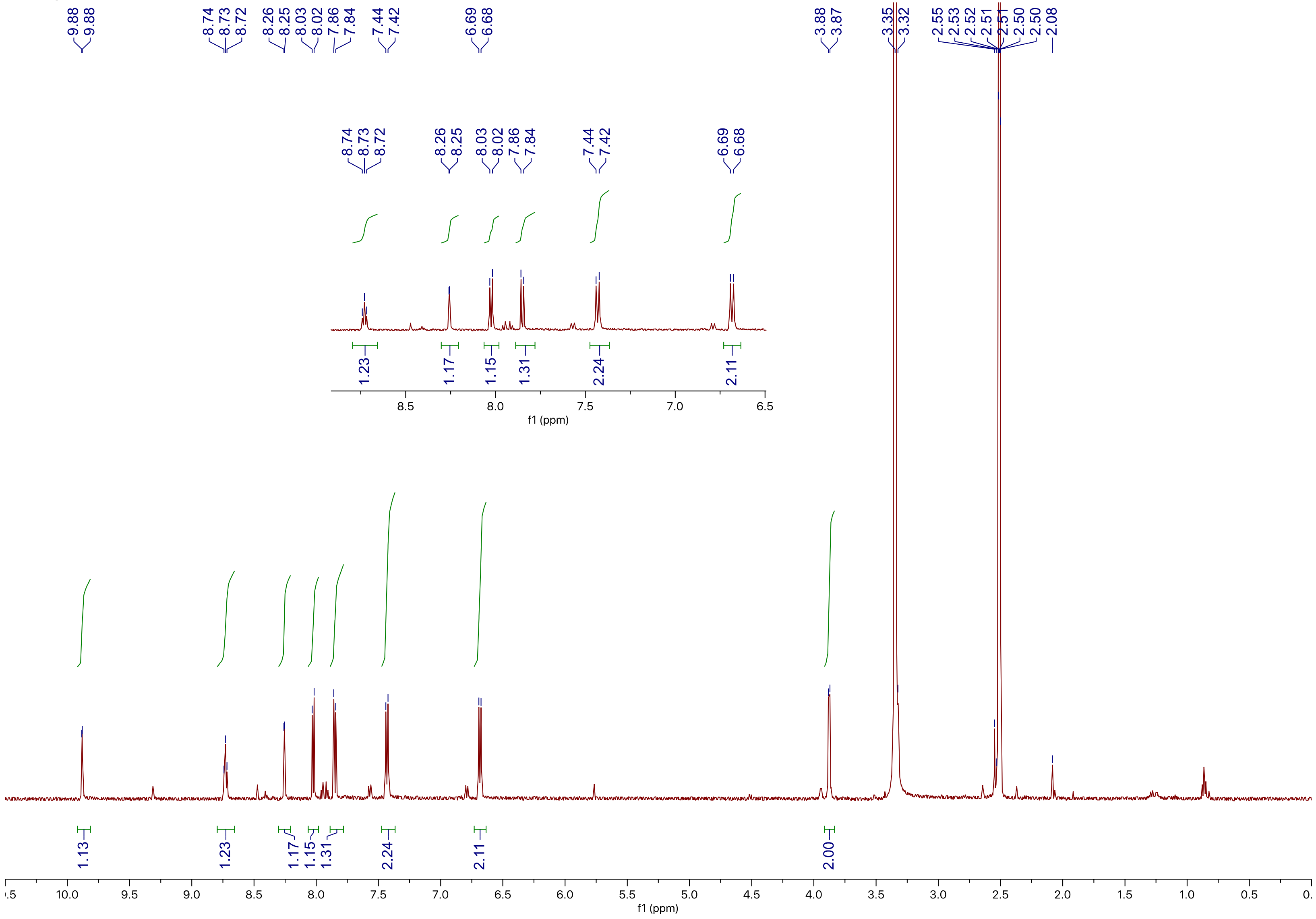
9.88
9.88
8.74
8.73
8.72
8.26
8.25
8.03
8.02
7.86
7.84
7.44
7.42
6.69
6.68
3.88
3.87
3.35
3.32
2.55
2.53
2.52
2.51
2.51
2.50
2.08
1.13
1.23
1.17
1.15
1.31
2.24
2.11
2.00
f1 (ppm)

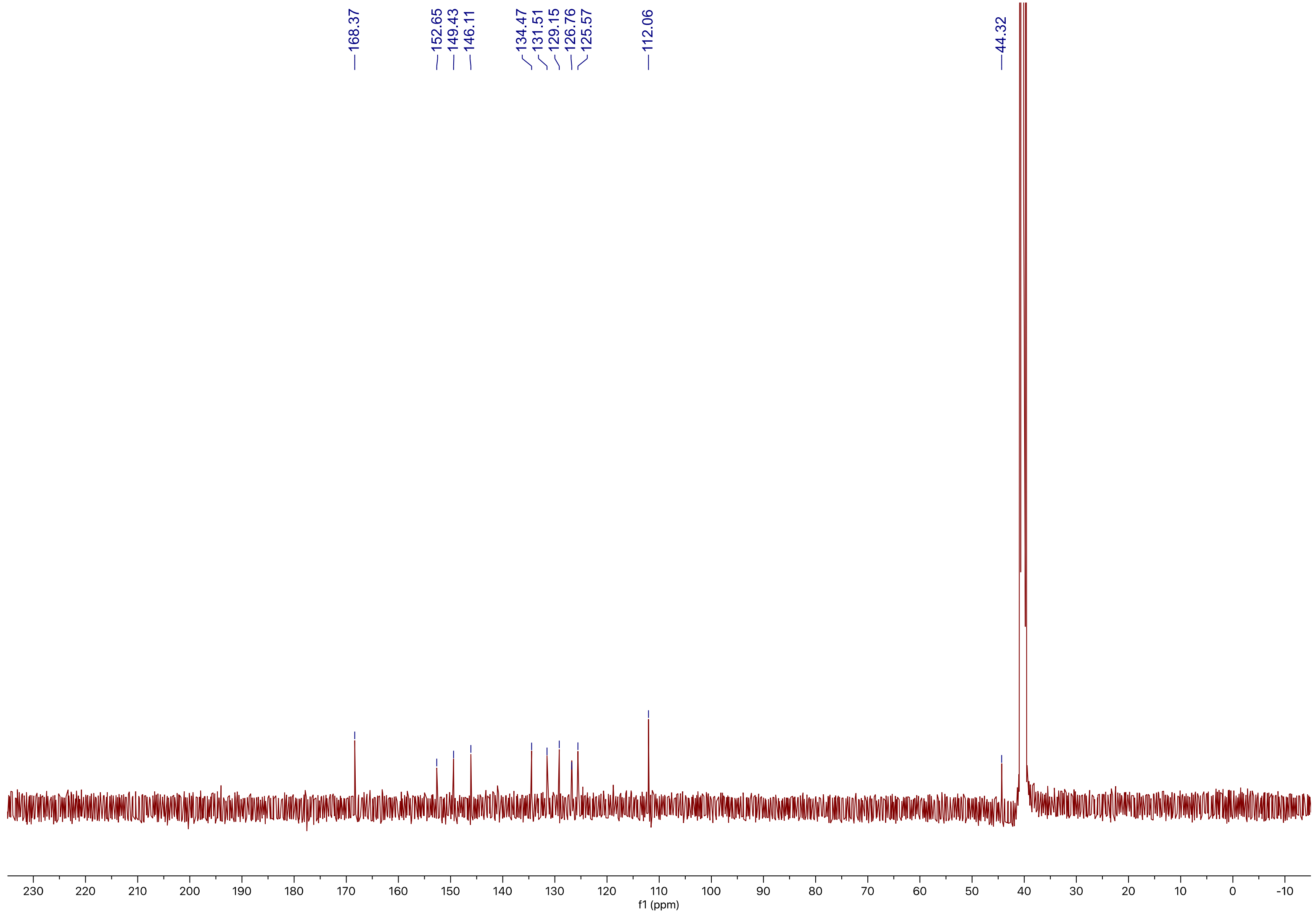
168.37
152.65
149.43
146.11
134.47
131.51
129.15
126.76
125.57
112.06
44.32
230
220
210
200
190
180
170
160
150
140
130
120
110
100
90
80
70
60
50
40
30
20
10
0
-10
f1 (ppm)

Compound 10

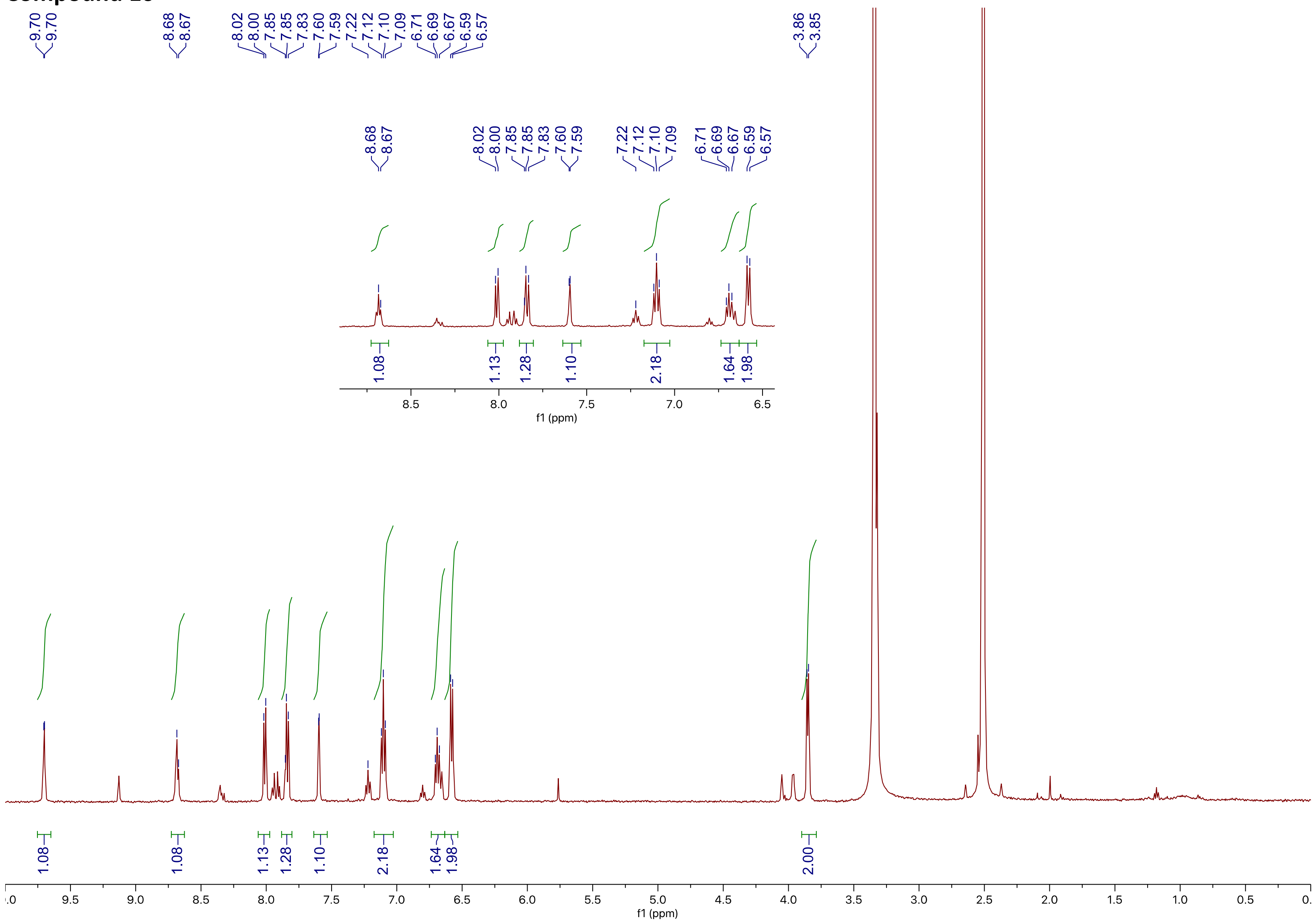
9.70
9.70
8.68
8.67
8.02
8.00
7.85
7.85
7.83
7.60
7.59
7.22
7.12
7.10
7.09
6.71
6.69
6.67
6.59
6.57
3.86
3.85
1.08
1.08
1.13
1.28
1.10
2.18
1.64
1.98
2.00
f1 (ppm)

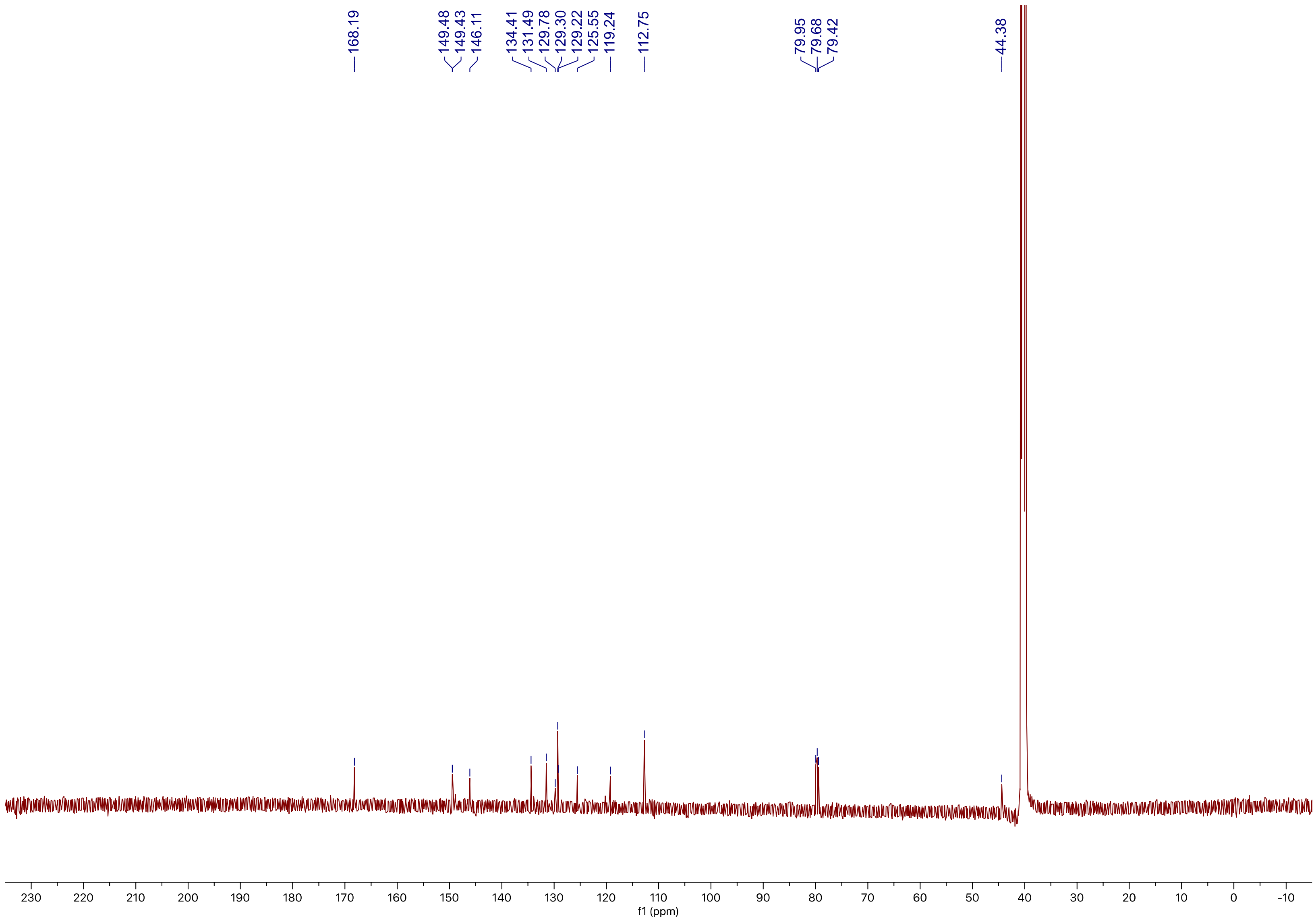
168.19
149.48
149.43
146.11
134.41
131.49
129.78
129.30
129.22
125.55
119.24
112.75
79.95
79.68
79.42
44.38
230
220
210
200
190
180
170
160
150
140
130
120
110
100
90
80
70
60
50
40
30
20
10
0
-10
f1 (ppm)

Compound 11

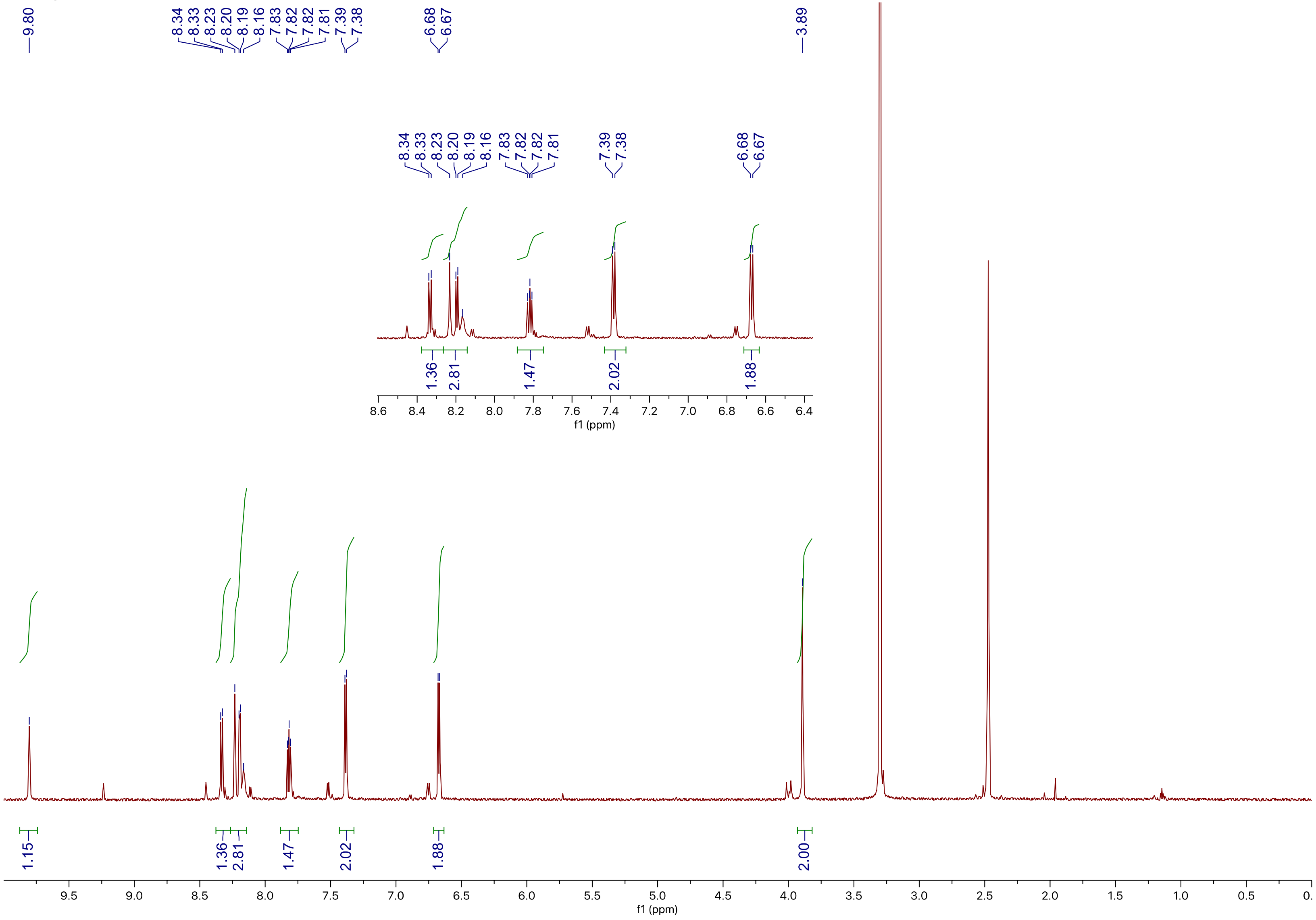
9.80
8.34
8.33
8.23
8.20
8.19
8.16
7.83
7.82
7.82
7.81
7.39
7.38
6.68
6.67
3.89
1.15
1.36
2.81
1.47
2.02
1.88
2.00
f1 (ppm)

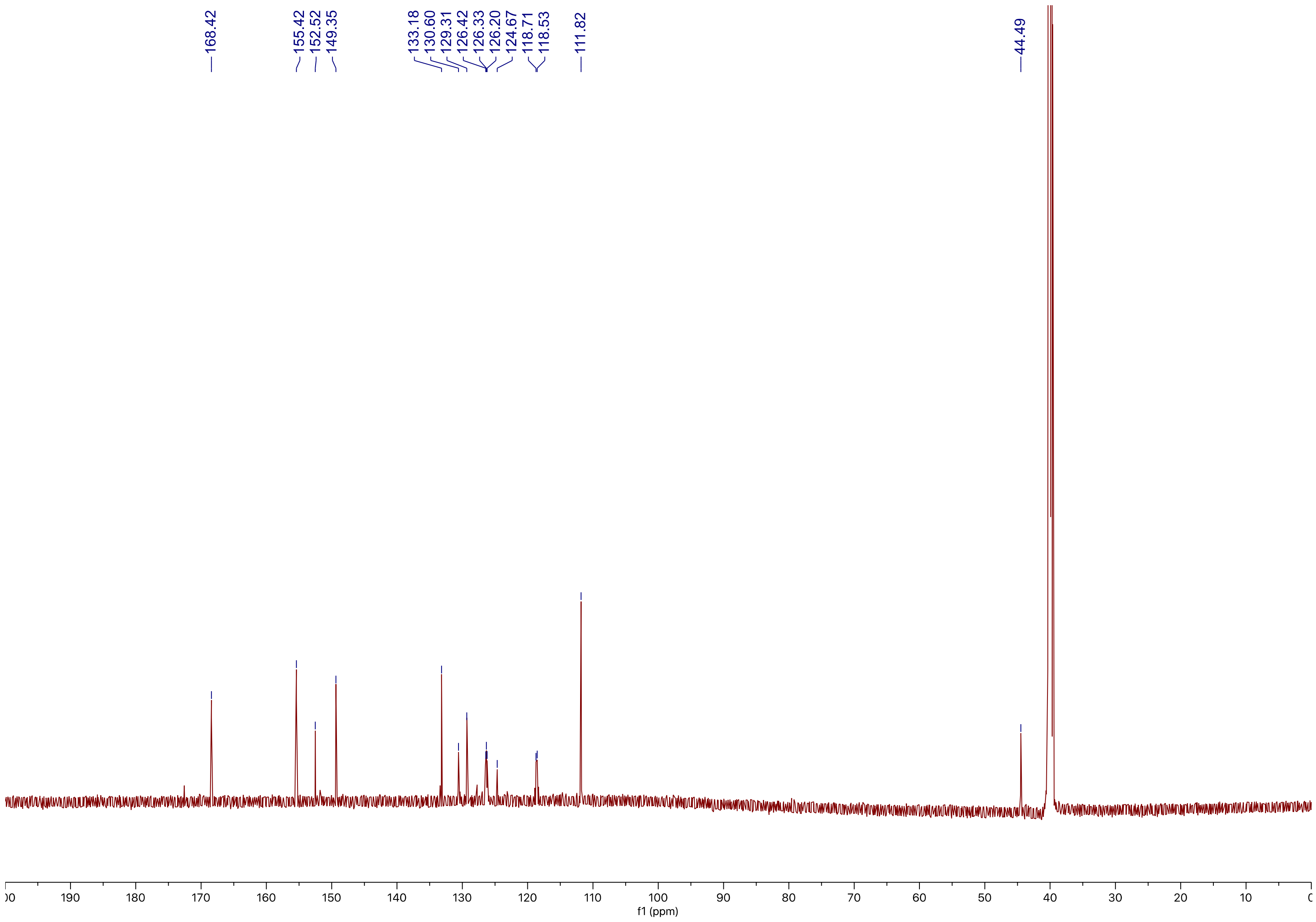

168.42
155.42
152.52
149.35
133.18
130.60
129.31
126.42
126.33
126.20
124.67
118.71
118.53
111.82
44.49
f1 (ppm)

Compound 12

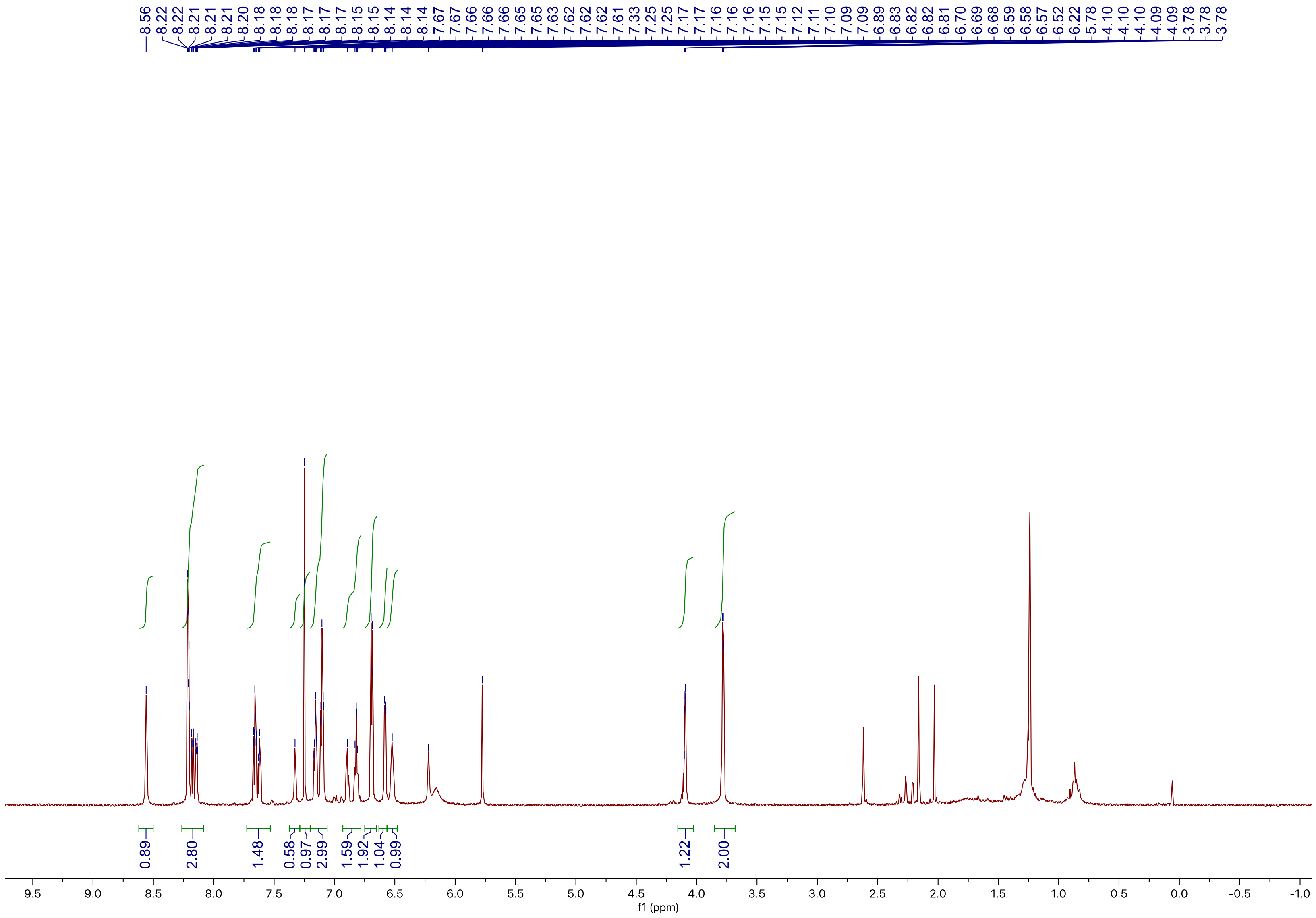

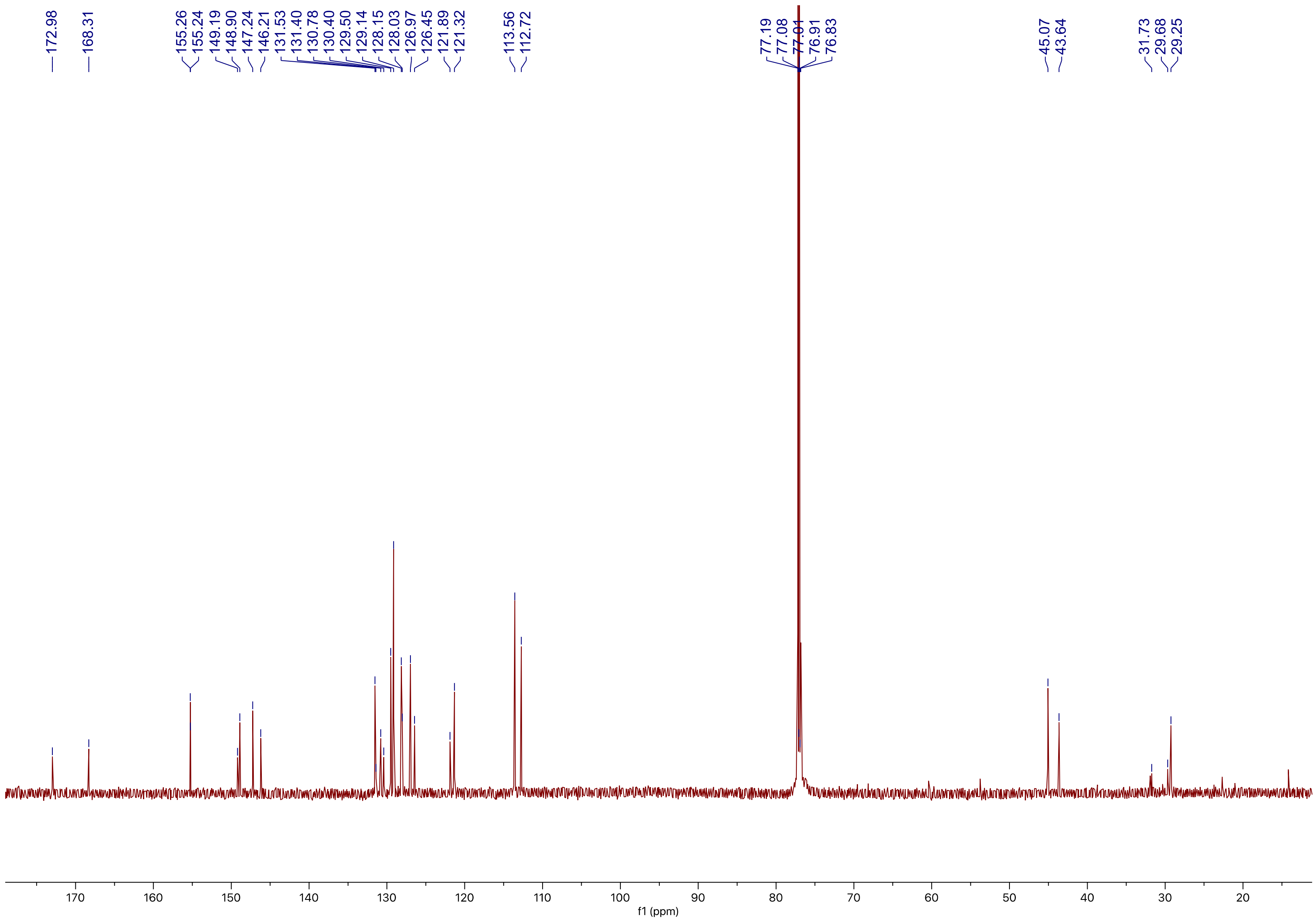

172.98
168.31
155.26
155.24
149.19
148.90
147.24
146.21
131.53
131.40
130.78
130.40
129.50
129.14
128.15
128.03
126.97
126.45
121.89
121.32
113.56
112.72
77.19
77.08
77.01
76.91
76.83
45.07
43.64
31.73
29.68
29.25
170
160
150
140
130
120
110
100
90
80
70
60
50
40
30
20
f1 (ppm)

Compound 13a

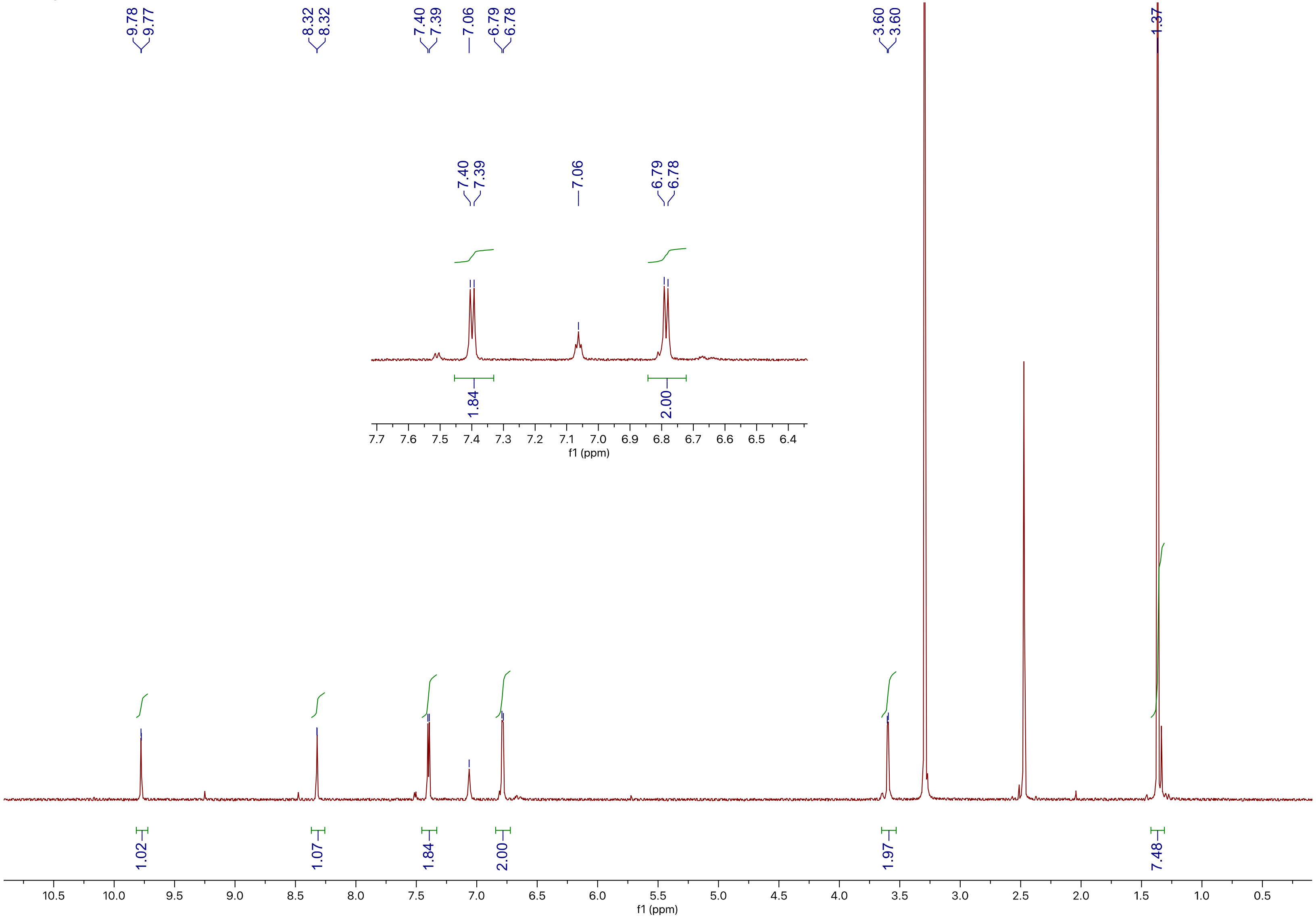

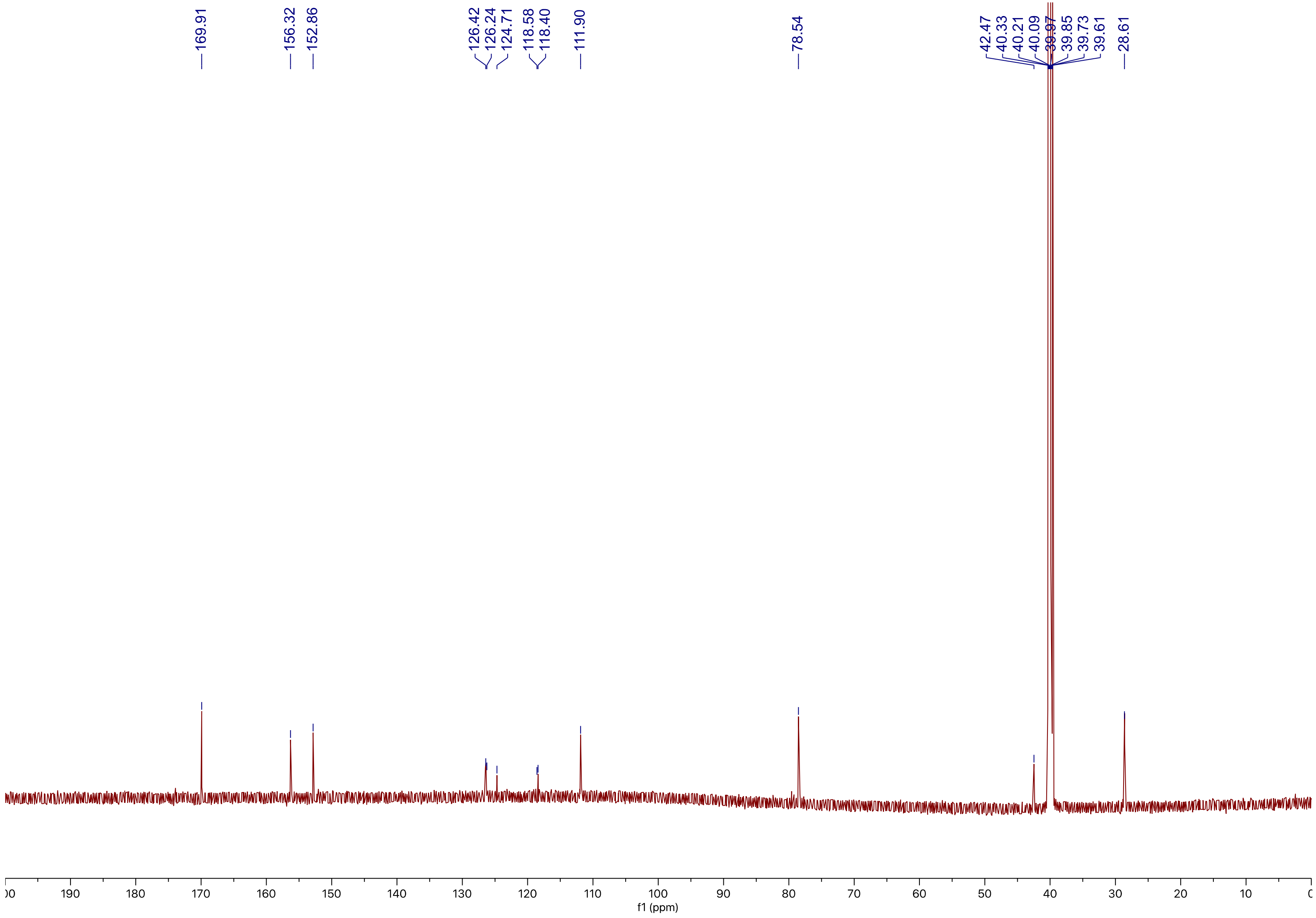

169.91
156.32
152.86
126.42
126.24
124.71
118.58
118.40
111.90
78.54
42.47
40.33
40.21
40.09
39.97
39.85
39.73
39.61
28.61
190
180
170
160
150
140
130
120
110
100
90
80
70
60
50
40
30
20
10
f1 (ppm)

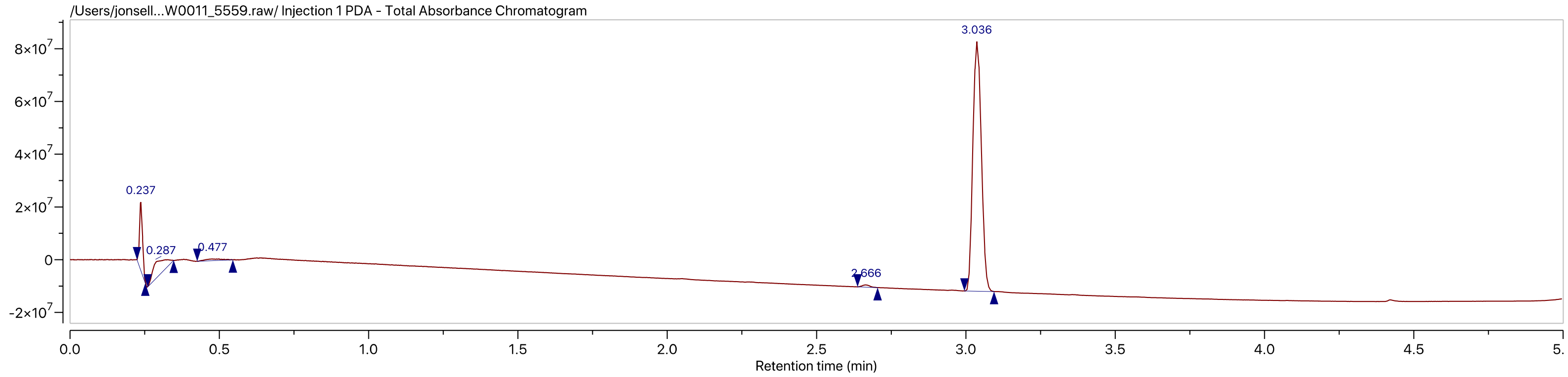
/Users/jonsell...W0011_5559.raw/ Injection 1 PDA - Total Absorbance Chromatogram
3.036
0.237
0.287
0.477
2.666
8x10^7
6x10^7
4x10^7
2x10^7
0
-2x10^7
0.0
0.5
1.0
1.5
2.0
2.5
3.0
3.5
4.0
4.5
5.
Retention time (min)

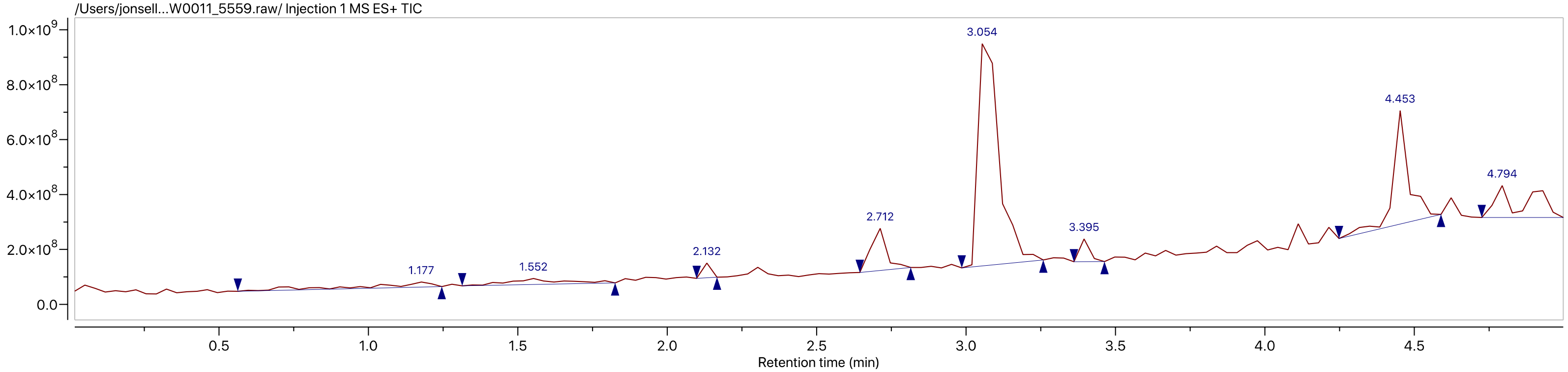
/Users/jonsell...W0011_5559.raw/ Injection 1 MS ES+ TIC
3.054
4.453
4.794
2.712
3.395
2.132
1.177
1.552
1.0x10^9
8.0x10^8
6.0x10^8
4.0x10^8
2.0x10^8
0.0
0.5
1.0
1.5
2.0
2.5
3.0
3.5
4.0
4.5
Retention time (min)

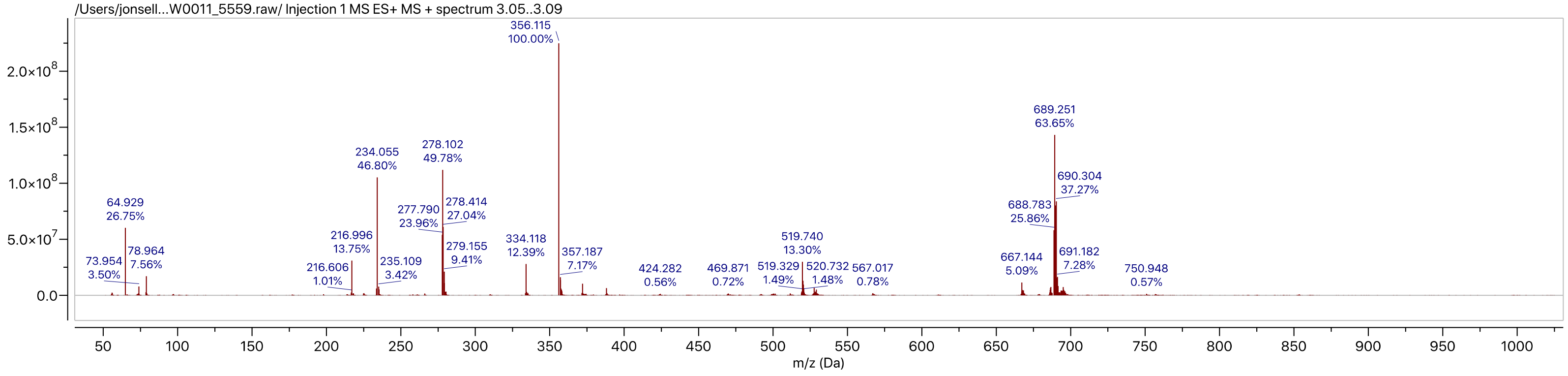
/Users/jonsell...W0011_5559.raw/ Injection 1 MS ES+ MS + spectrum 3.05..3.09
356.115
100.00%
689.251
63.65%
278.102
49.78%
234.055
46.80%
690.304
37.27%
64.929
26.75%
278.414
27.04%
277.790
23.96%
688.783
25.86%
216.996
13.75%
519.740
13.30%
334.118
12.39%
279.155
9.41%
78.964
7.56%
357.187
7.17%
691.182
7.28%
73.954
3.50%
216.606
1.01%
235.109
3.42%
667.144
5.09%
424.282
0.56%
469.871
0.72%
519.329
1.49%
520.732
1.48%
567.017
0.78%
750.948
0.57%
2.0x10^8
1.5x10^8
1.0x10^8
5.0x10^7
0.0
50
100
150
200
250
300
350
400
450
500
550
600
650
700
750
800
850
900
950
1000
m/z (Da)

Compound 14a

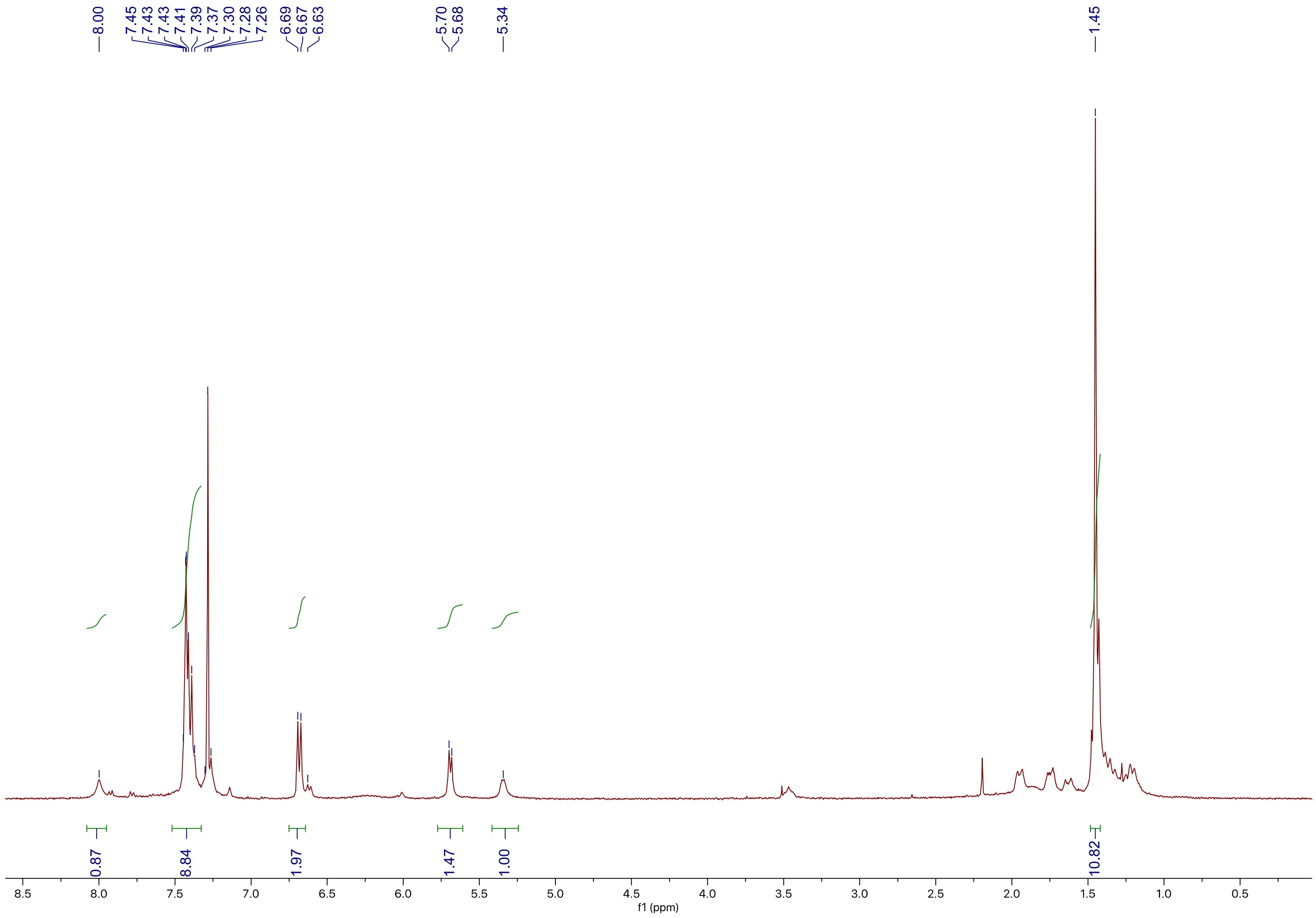

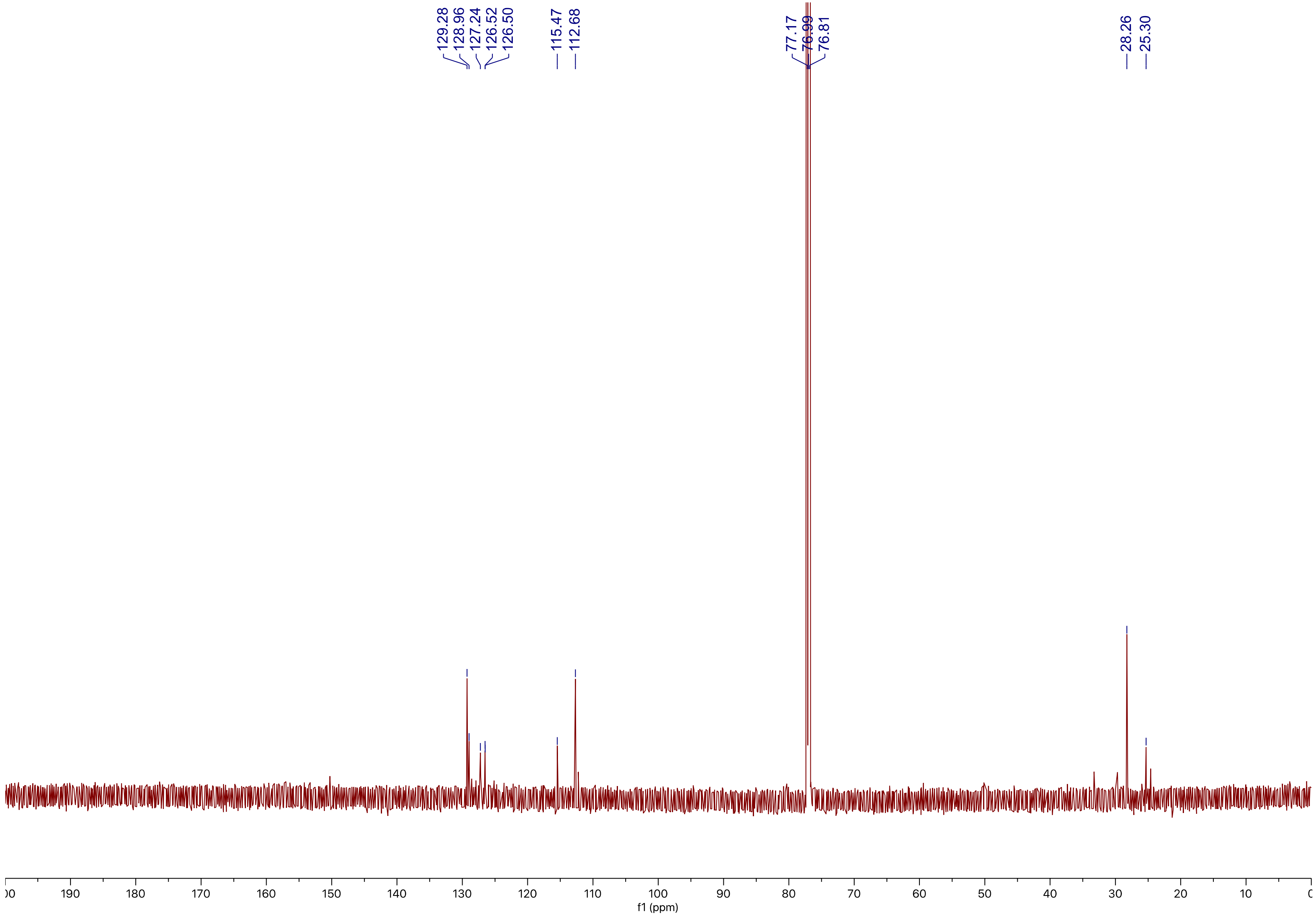
129.28
128.96
127.24
126.52
126.50
115.47
112.68
77.17
76.99
76.81
28.26
25.30
f1 (ppm)

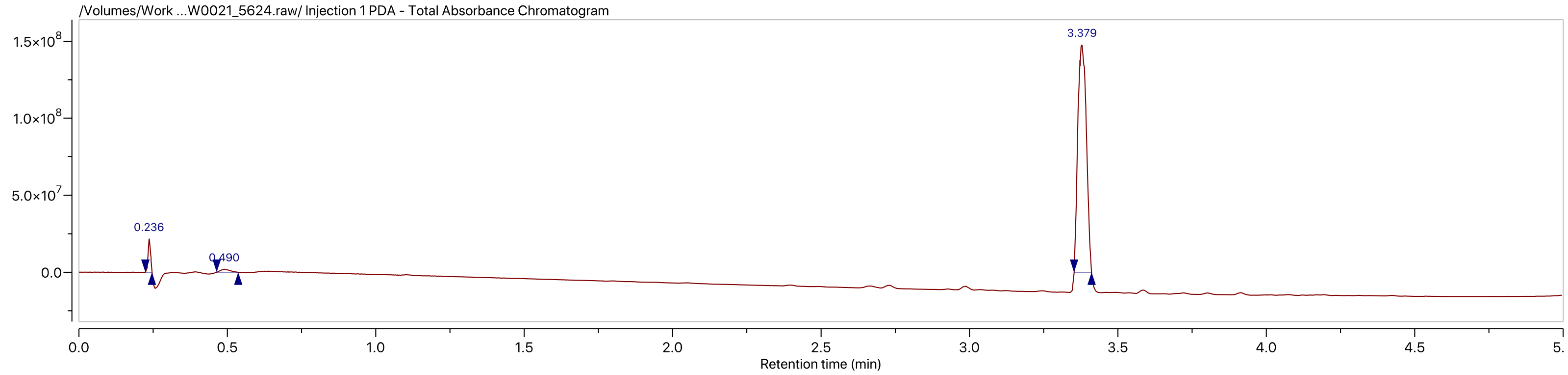
/Volumes/Work ...W0021_5624.raw/ Injection 1 PDA - Total Absorbance Chromatogram
3.379
0.236
0.490
Retention time (min)

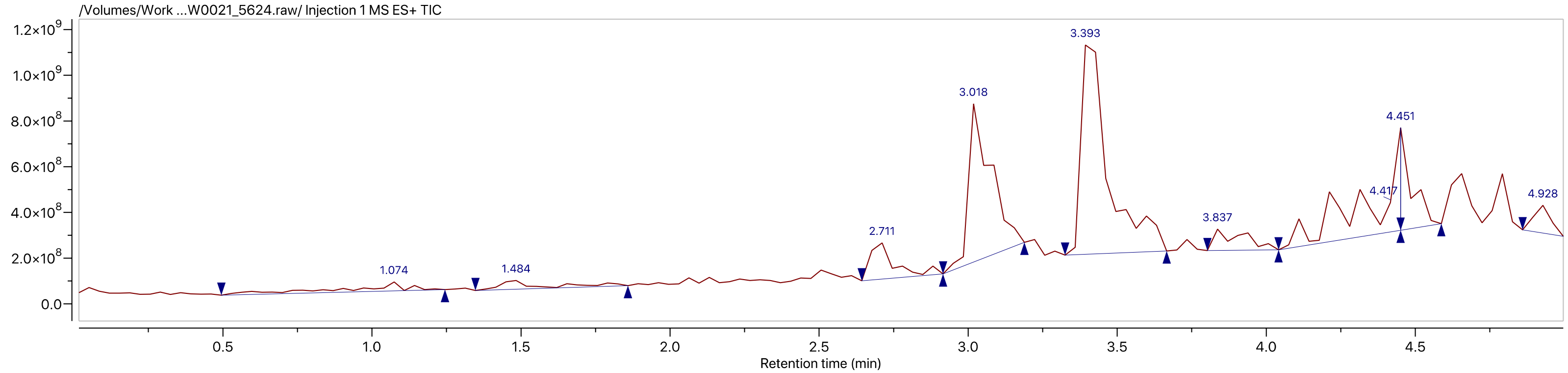
/Volumes/Work ...W0021_5624.raw/ Injection 1 MS ES+ TIC
3.393
3.018
4.451
2.711
1.074
1.484
3.837
4.417
4.928
Retention time (min)

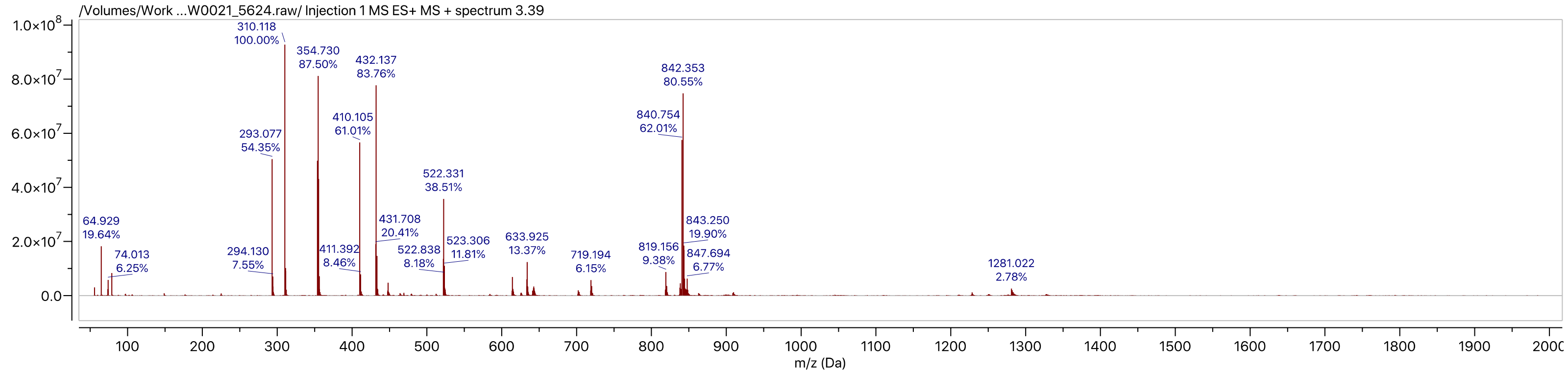
/Volumes/Work ...W0021_5624.raw/ Injection 1 MS ES+ MS + spectrum 3.39
310.118 100.00%
354.730 87.50%
432.137 83.76%
842.353 80.55%
840.754 62.01%
410.105 61.01%
293.077 54.35%
522.331 38.51%
64.929 19.64%
74.013 6.25%
294.130 7.55%
411.392 8.46%
431.708 20.41%
522.838 8.18%
523.306 11.81%
633.925 13.37%
719.194 6.15%
819.156 9.38%
843.250 19.90%
847.694 6.77%
1281.022 2.78%
m/z (Da)

Compound 14b

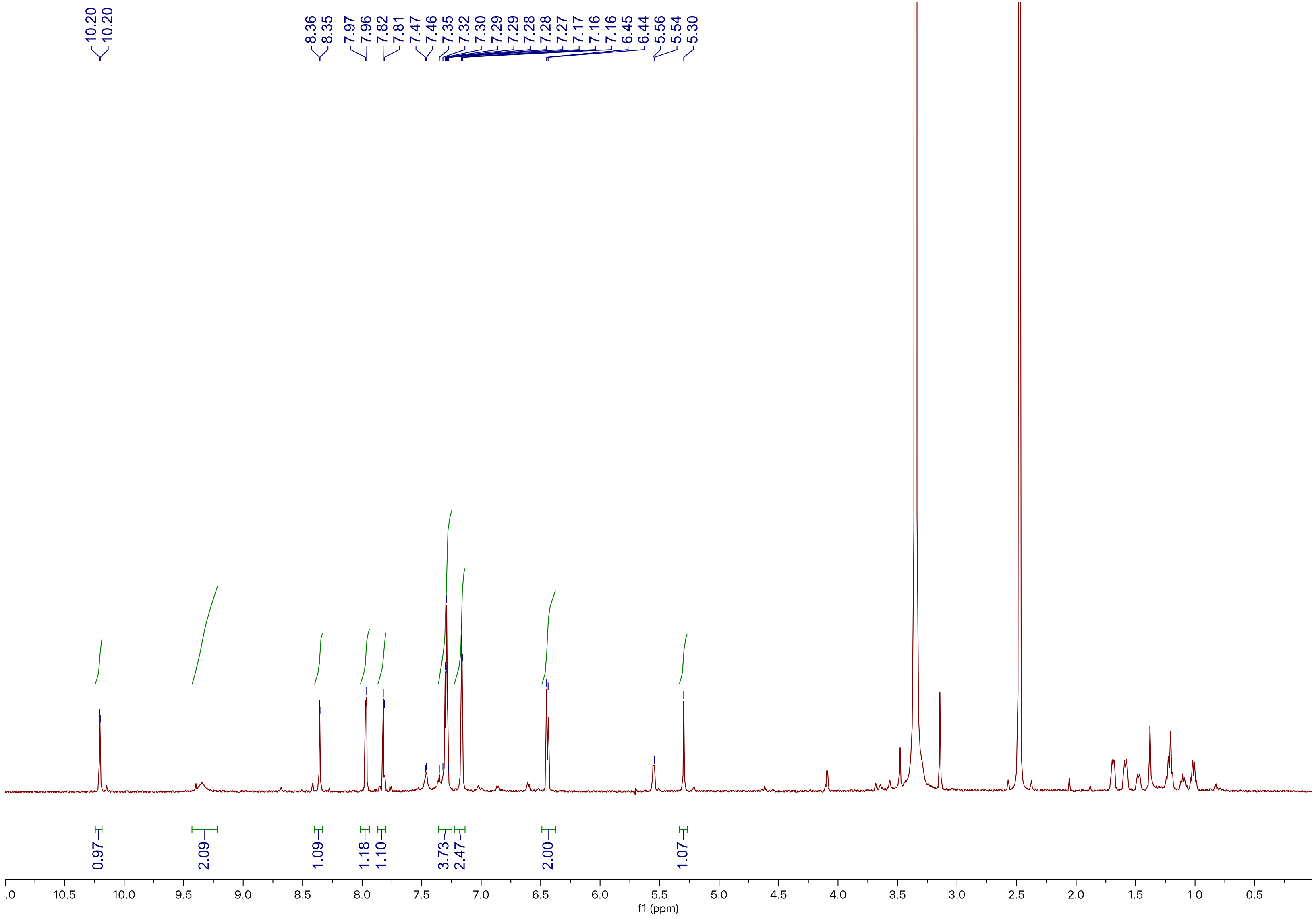

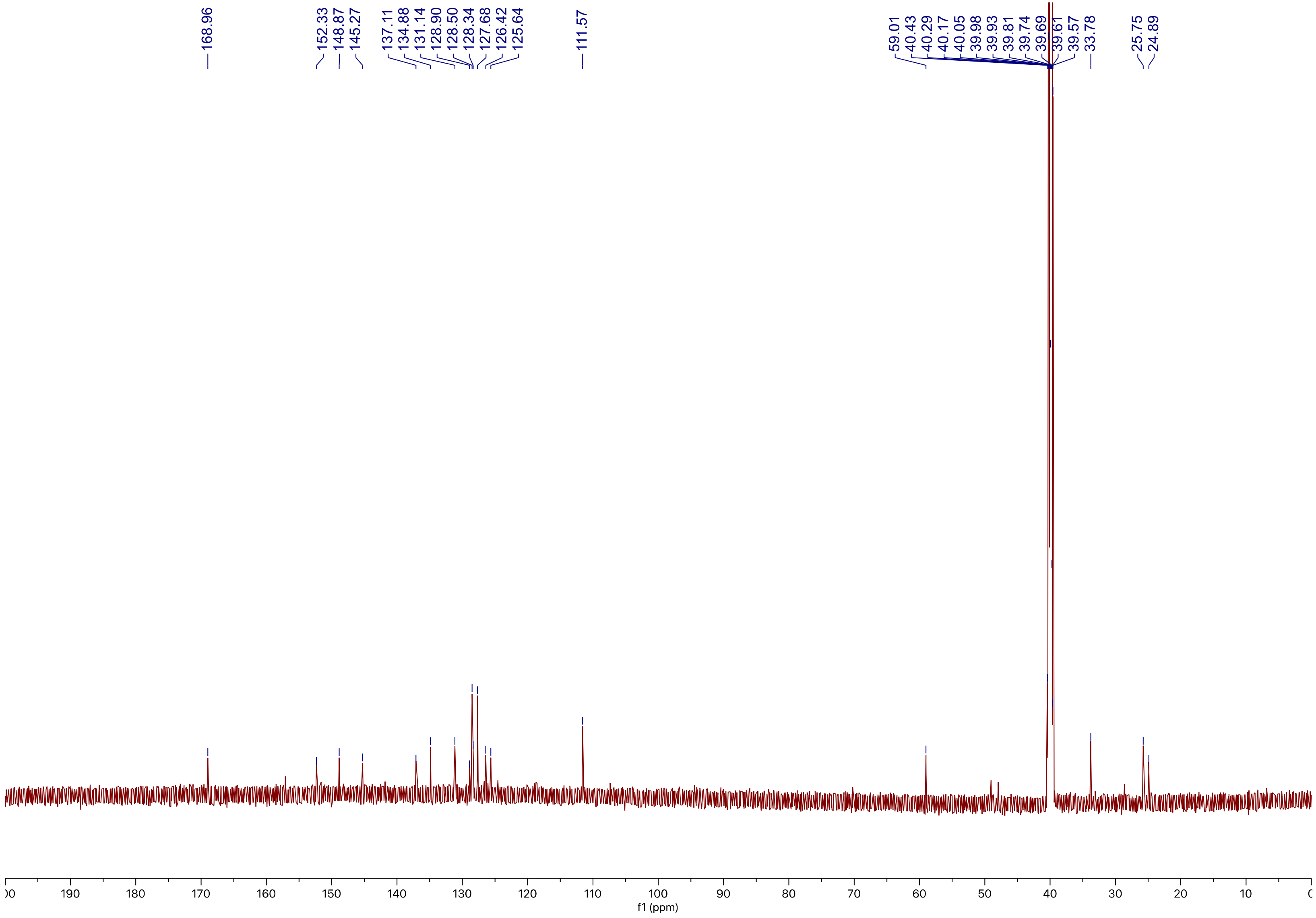

168.96
152.33
148.87
145.27
137.11
134.88
131.14
128.90
128.50
128.34
127.68
126.42
125.64
111.57
59.01
40.43
40.29
40.17
40.05
39.98
39.93
39.81
39.74
39.69
39.61
39.57
33.78
25.75
24.89
f1 (ppm)

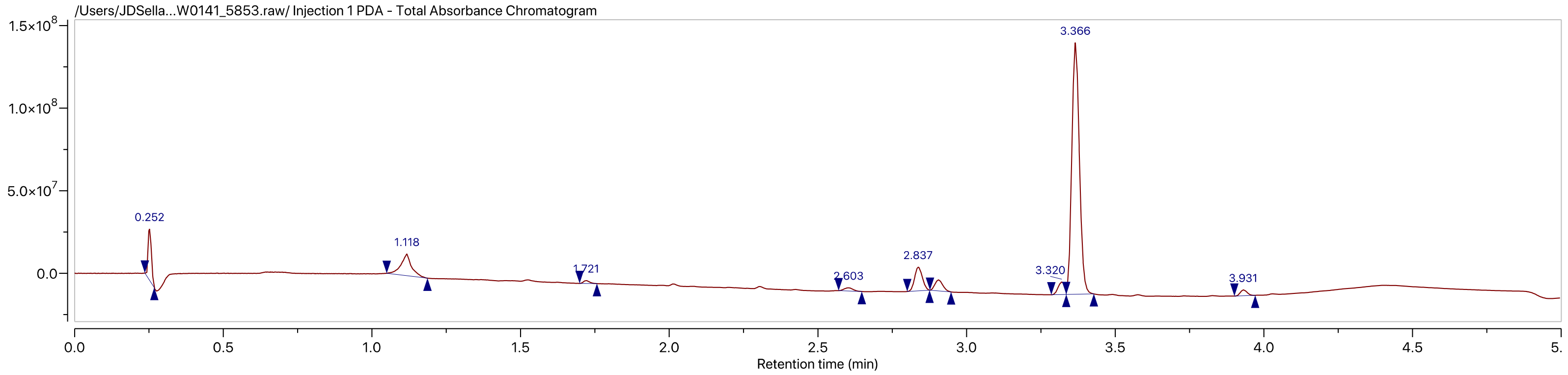
/Users/JDSella...W0141_5853.raw/ Injection 1 PDA - Total Absorbance Chromatogram
0.252
1.118
1.721
2.603
2.837
3.320
3.366
3.931
Retention time (min)

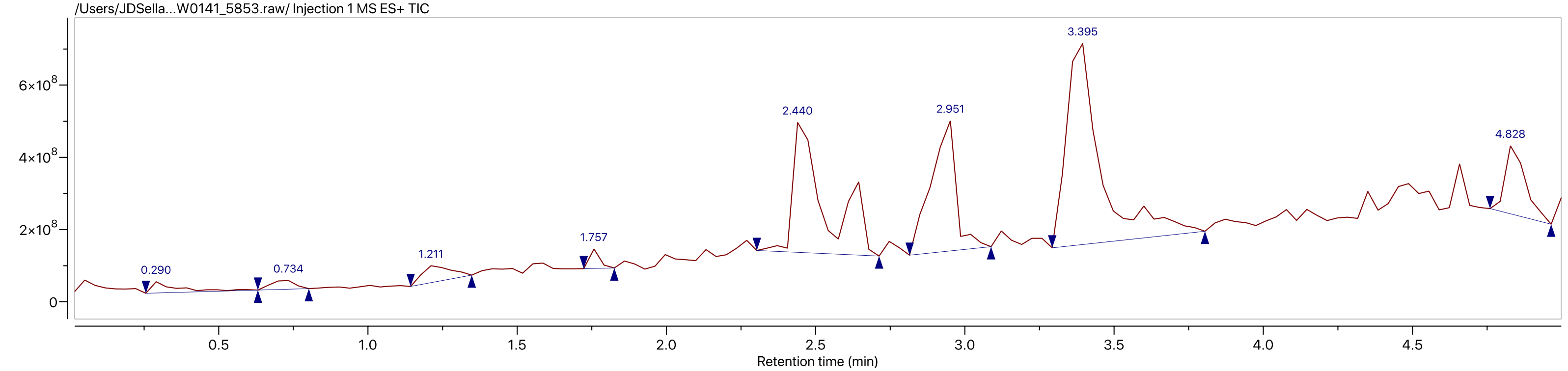
/Users/JDSella...W0141_5853.raw/ Injection 1 MS ES+ TIC
0.290
0.734
1.211
1.757
2.440
2.951
3.395
4.828
Retention time (min)

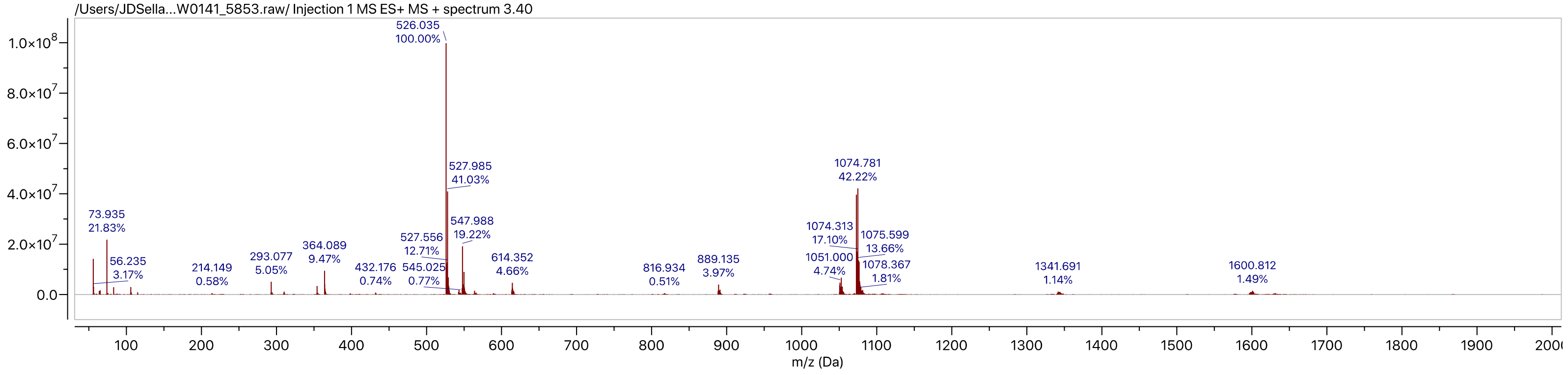
/Users/JDSella...W0141_5853.raw/ Injection 1 MS ES+ MS + spectrum 3.40
526.035
100.00%
527.985
41.03%
547.988
19.22%
527.556
12.71%
545.025
0.77%
73.935
21.83%
56.235
3.17%
214.149
0.58%
293.077
5.05%
364.089
9.47%
432.176
0.74%
614.352
4.66%
816.934
0.51%
889.135
3.97%
1074.781
42.22%
1074.313
17.10%
1075.599
13.66%
1051.000
4.74%
1078.367
1.81%
1341.691
1.14%
1600.812
1.49%
m/z (Da)

Compound 15a

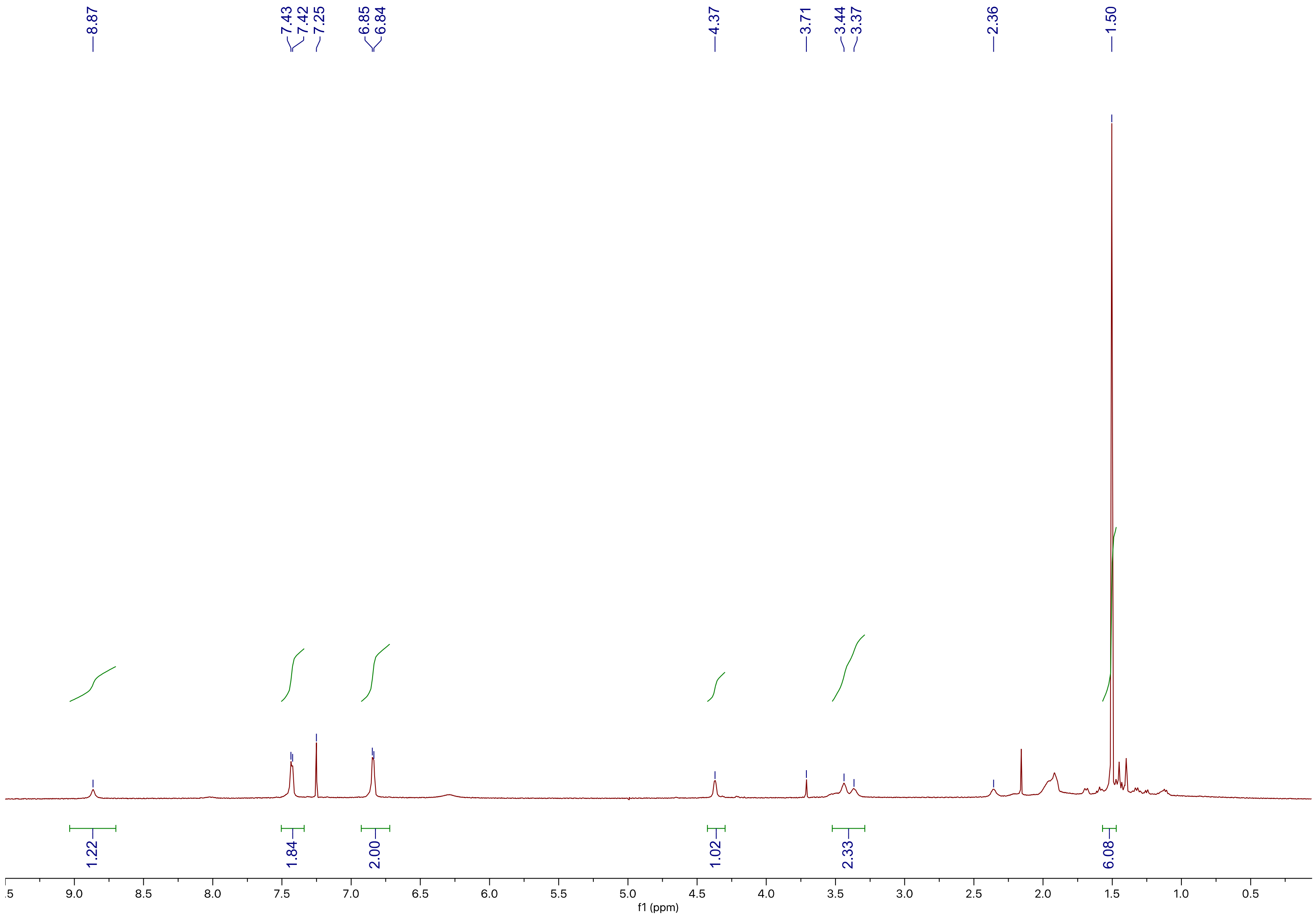

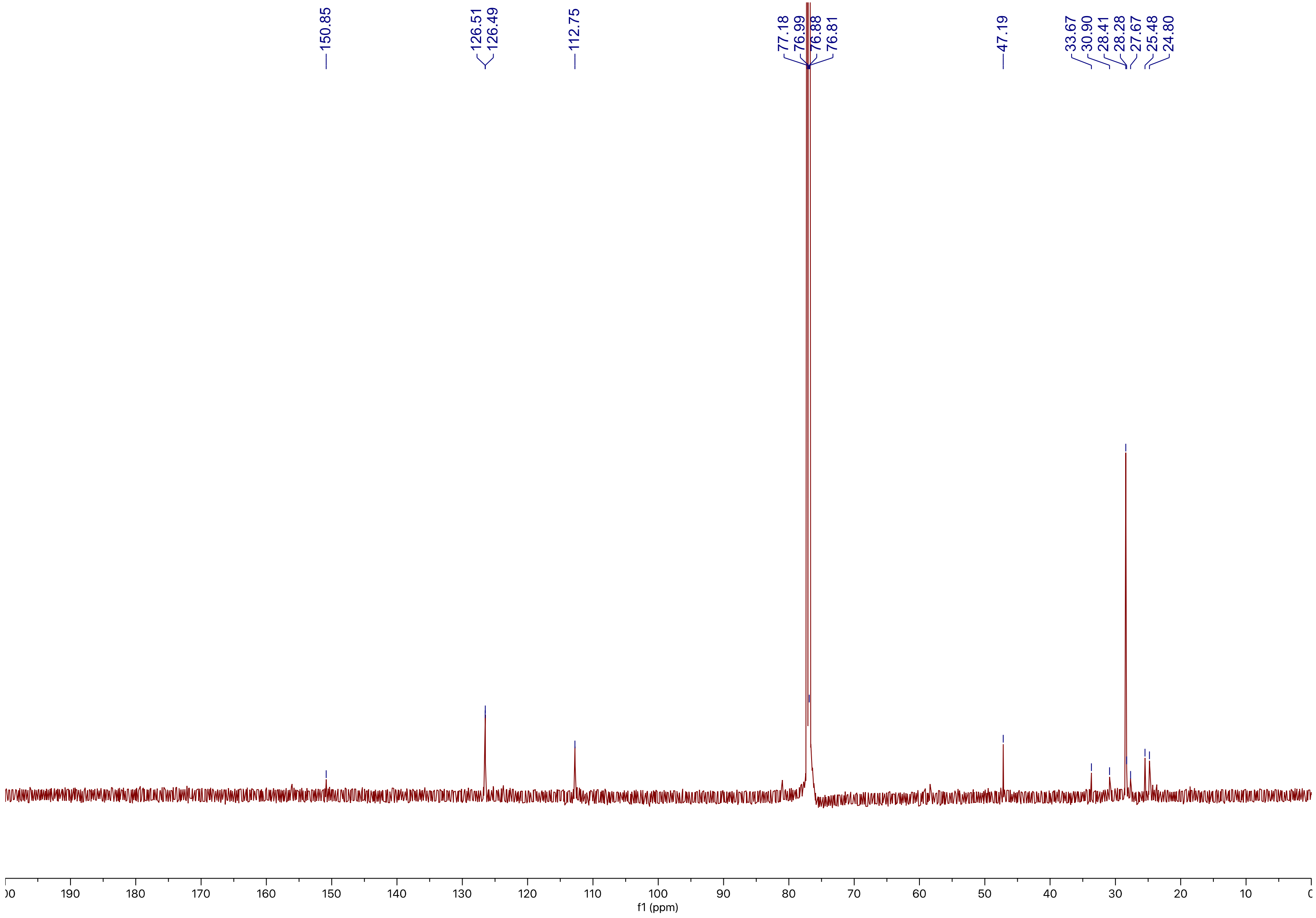

150.85
126.51
126.49
112.75
77.18
76.99
76.88
76.81
47.19
33.67
30.90
28.41
28.28
27.67
25.48
24.80
190
180
170
160
150
140
130
120
110
100
90
80
70
60
50
40
30
20
10
f1 (ppm)

/Users/JDSella...W0151_5935.raw/ Injection 1 PDA - Total Absorbance Chromatogram

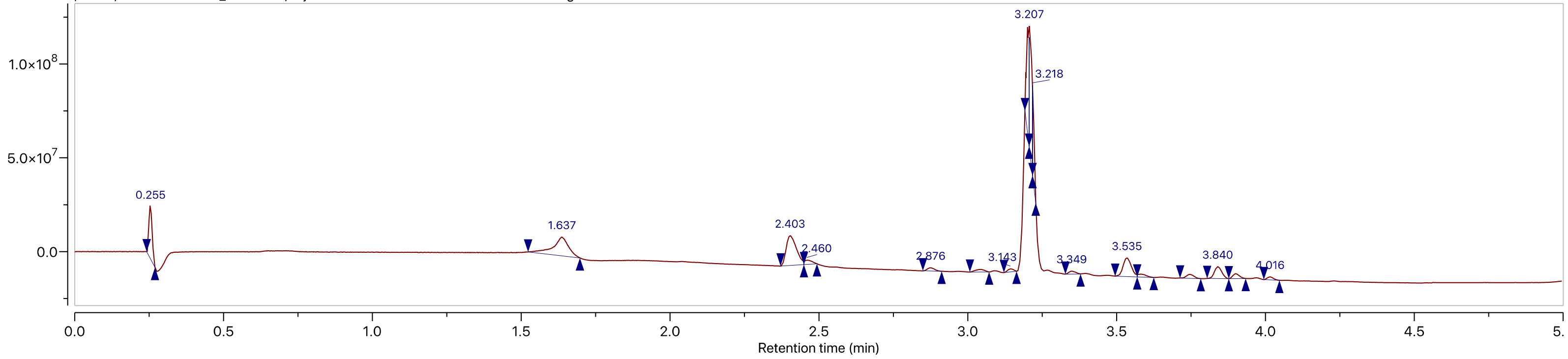

/Users/JDSella...W0151_5935.raw/ Injection 1 MS ES+ TIC

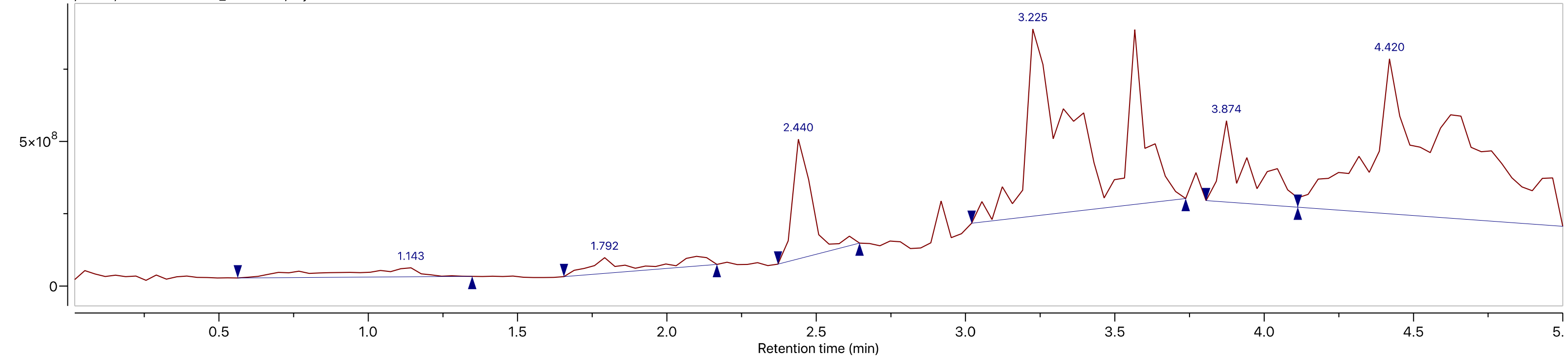

/Users/JDSella...W0151_5935.raw/ Injection 1 MS ES+ MS + spectrum 3.23

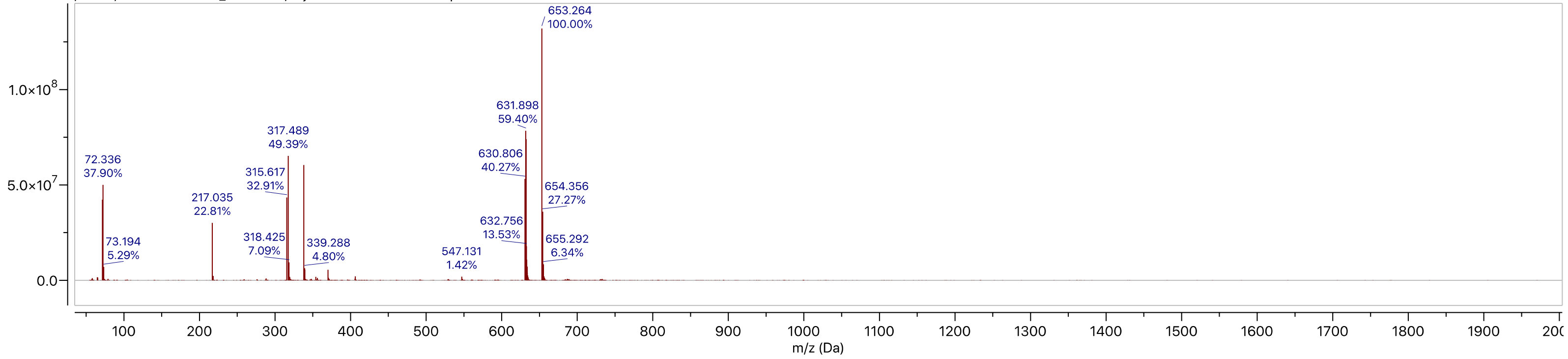

Compound 15b

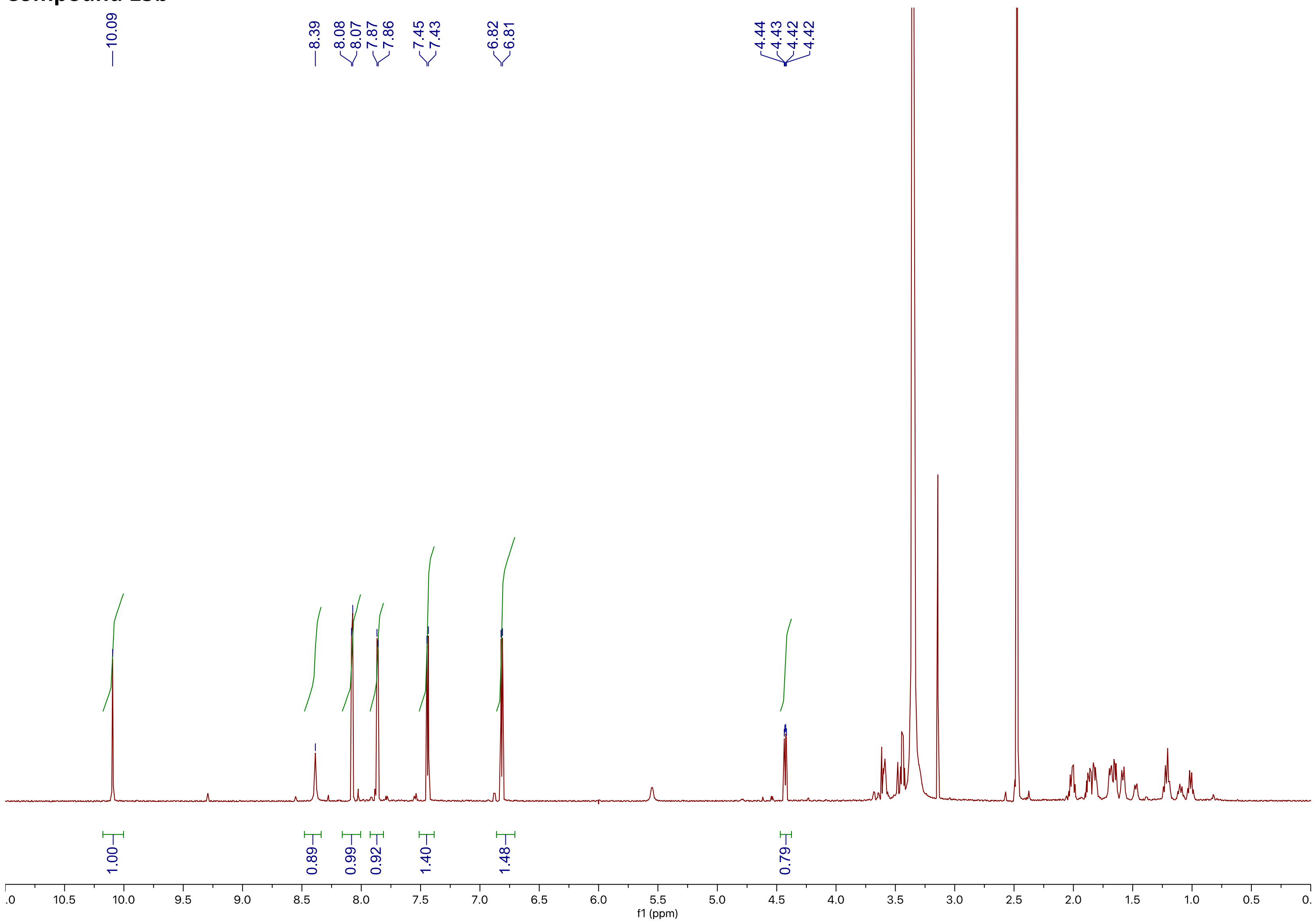

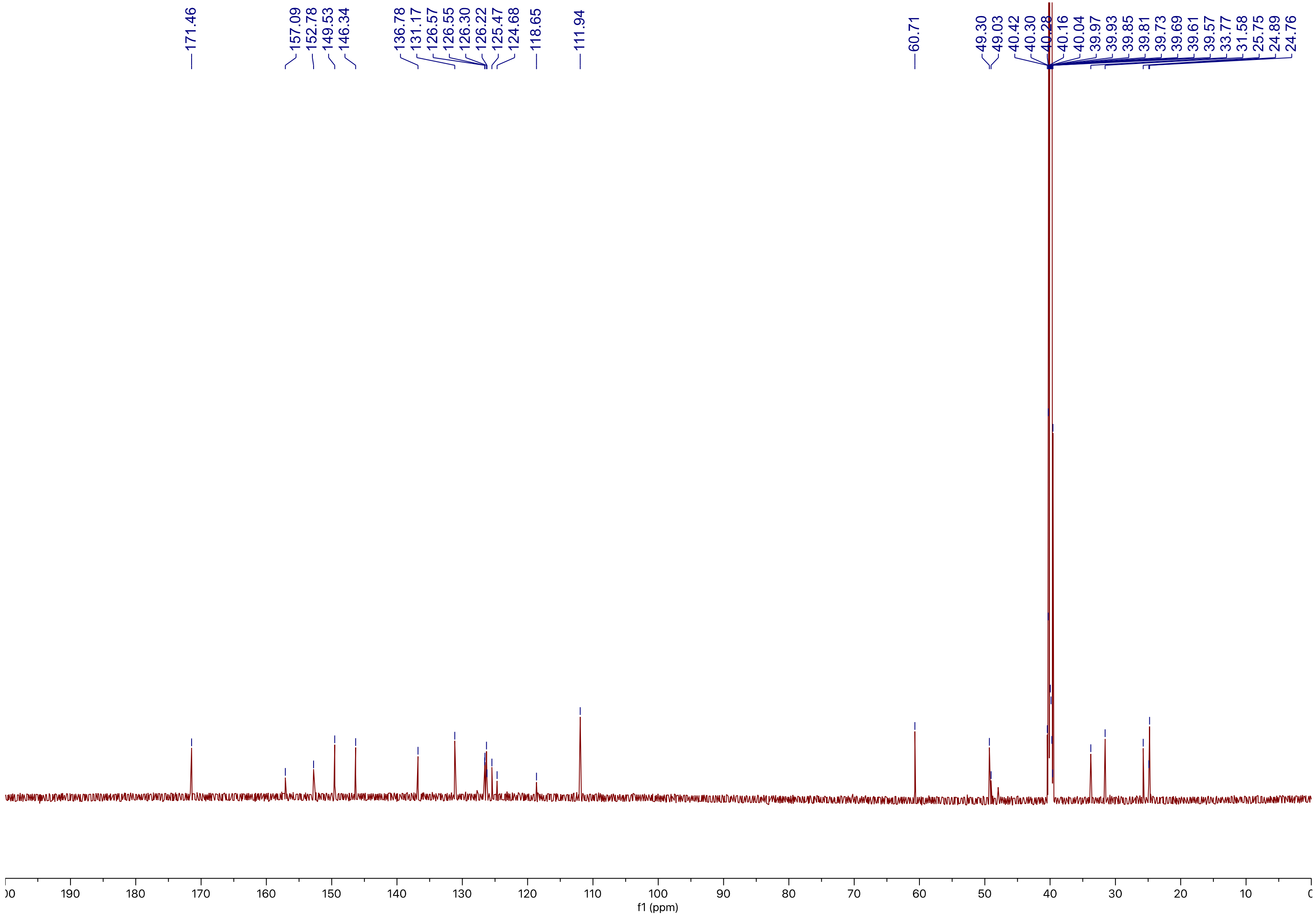
171.46
157.09
152.78
149.53
146.34
136.78
131.17
126.57
126.55
126.30
126.22
125.47
124.68
118.65
111.94
60.71
49.30
49.03
40.42
40.30
40.28
40.16
40.04
39.97
39.93
39.85
39.81
39.73
39.69
39.61
39.57
33.77
31.58
25.75
24.89
24.76
190
180
170
160
150
140
130
120
110
100
90
80
70
60
50
40
30
20
10
f1 (ppm)

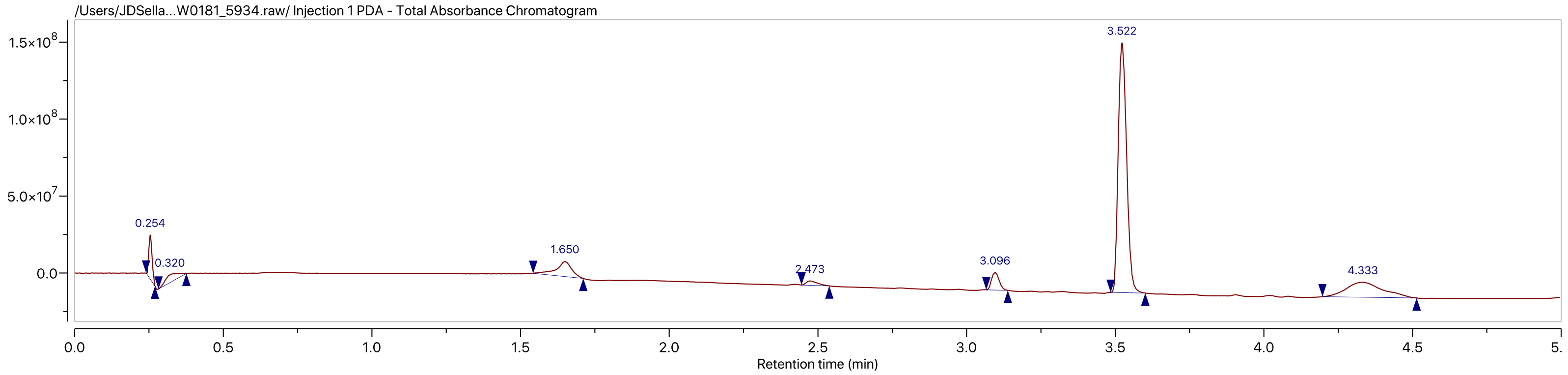
/Users/JDSella...W0181_5934.raw/ Injection 1 PDA - Total Absorbance Chromatogram
Retention time (min)
0.254
0.320
1.650
2.473
3.096
3.522
4.333

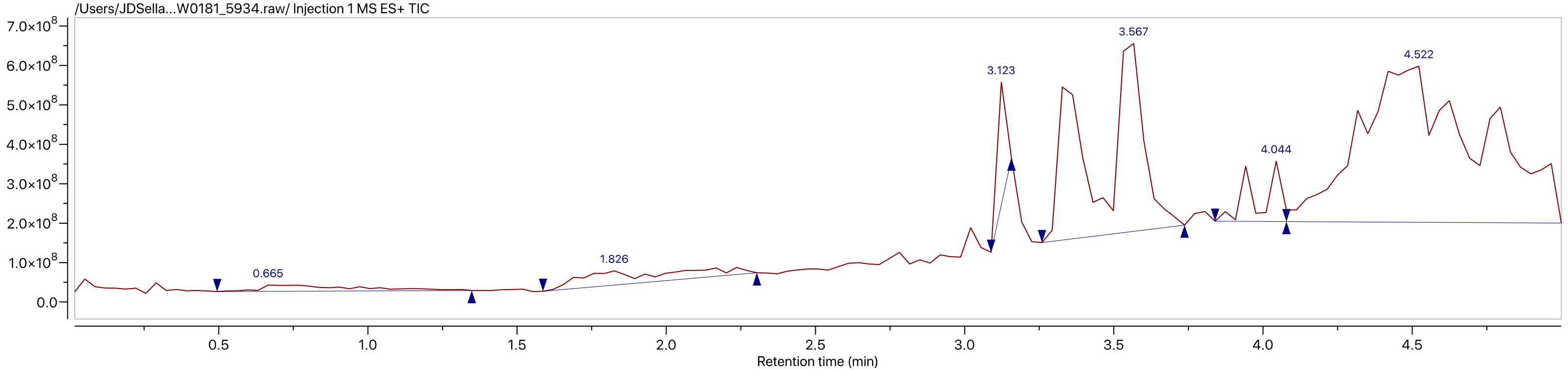
/Users/JDSella...W0181_5934.raw/ Injection 1 MS ES+ TIC
Retention time (min)
0.665
1.826
3.123
3.567
4.044
4.522

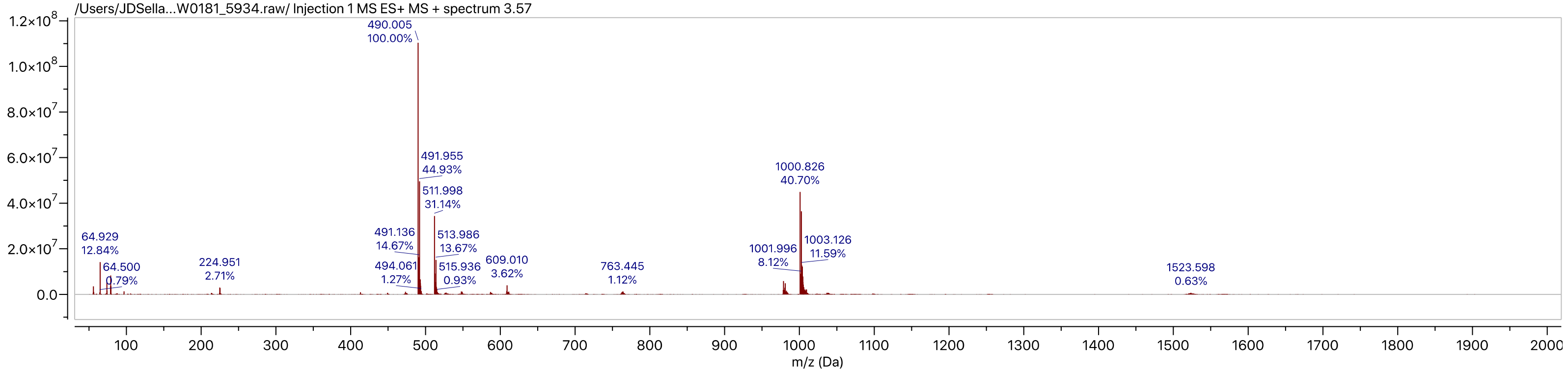
/Users/JDSella...W0181_5934.raw/ Injection 1 MS ES+ MS + spectrum 3.57
m/z (Da)
490.005
100.00%
491.955
44.93%
511.998
31.14%
491.136
14.67%
513.986
13.67%
494.061
1.27%
515.936
0.93%
609.010
3.62%
64.929
12.84%
64.500
0.79%
224.951
2.71%
763.445
1.12%
1000.826
40.70%
1001.996
8.12%
1003.126
11.59%
1523.598
0.63%

Compound 16a

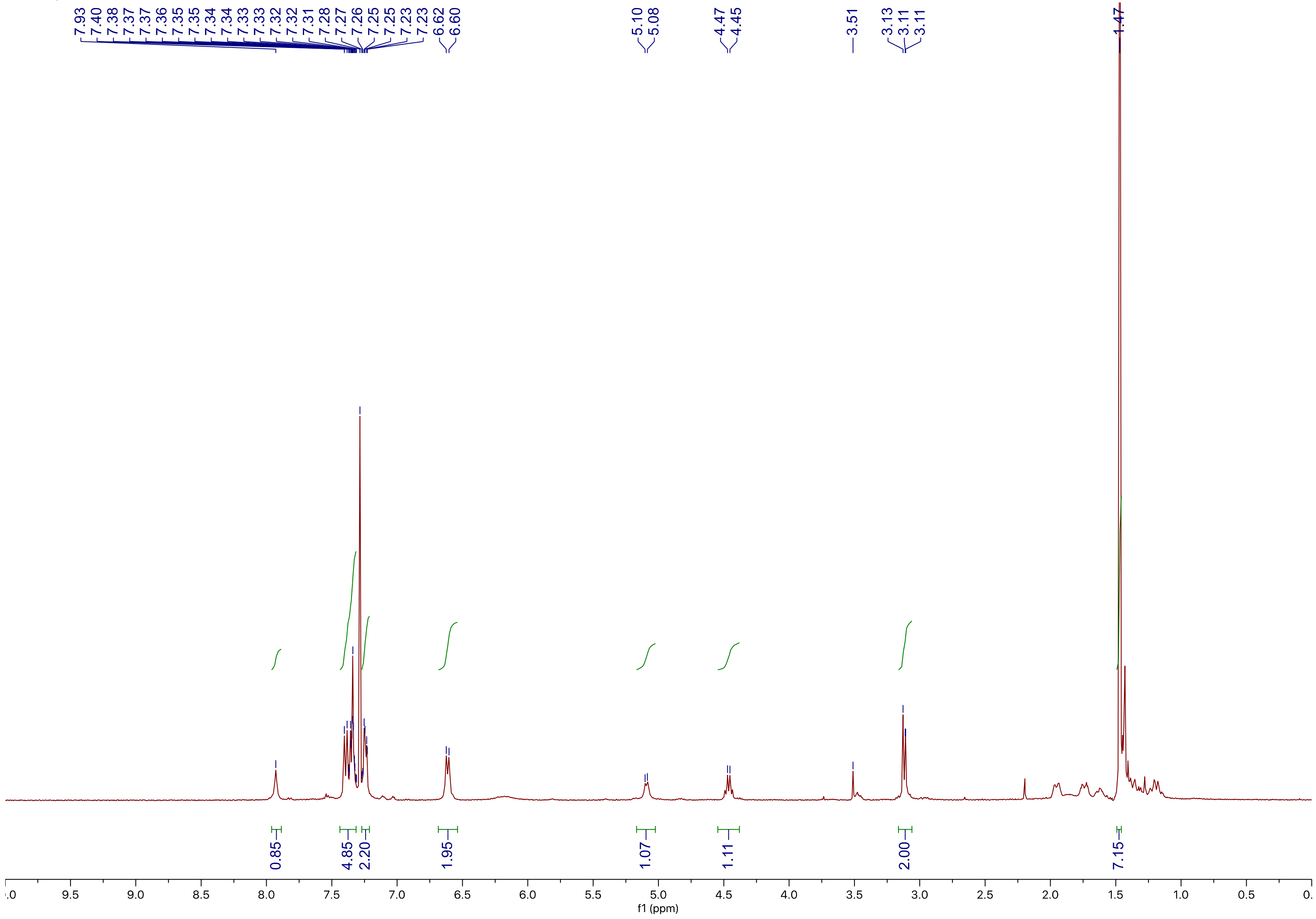

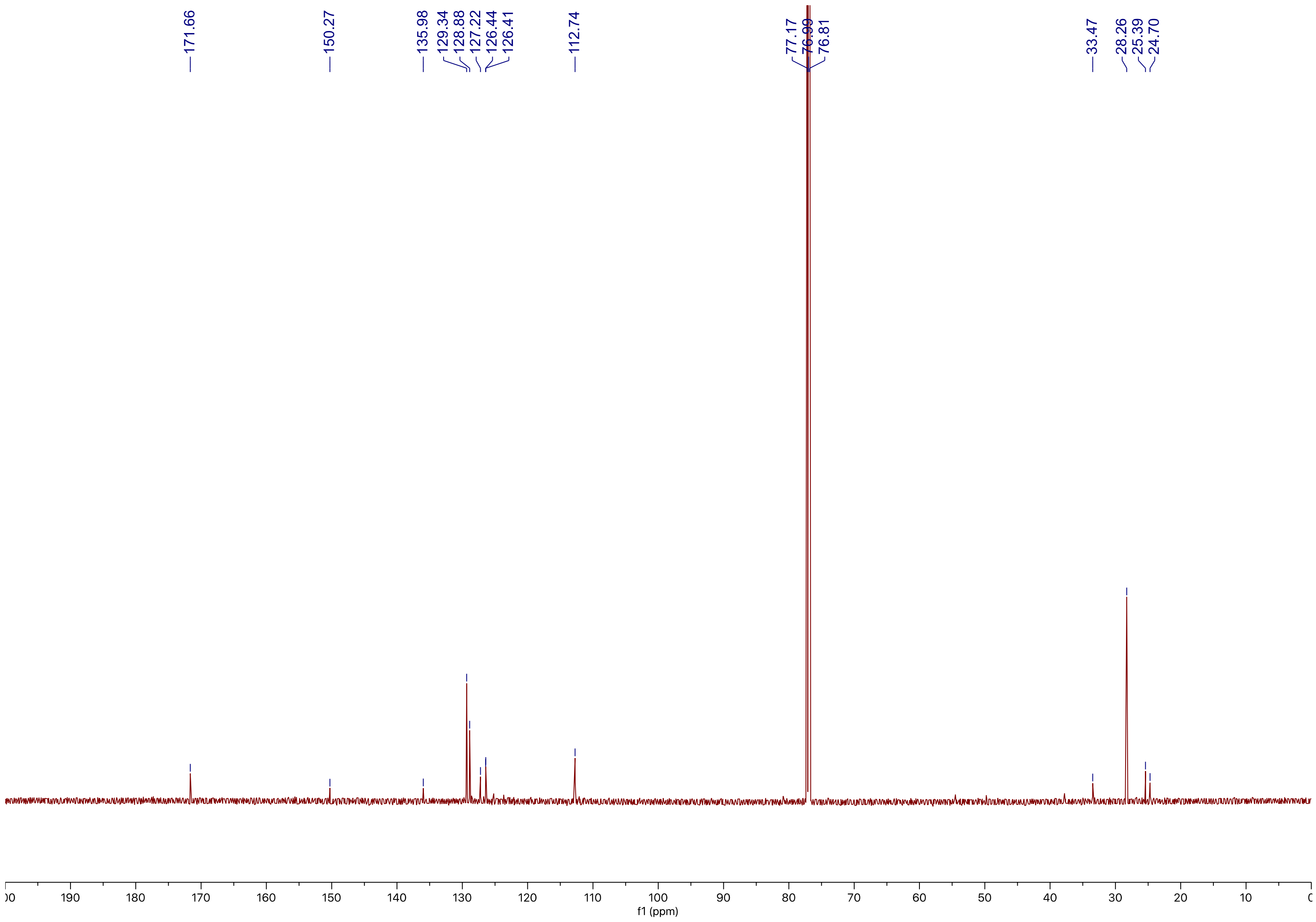

171.66
150.27
135.98
129.34
128.88
127.22
126.44
126.41
112.74
77.17
76.99
76.81
33.47
28.26
25.39
24.70
190
180
170
160
150
140
130
120
110
100
90
80
70
60
50
40
30
20
10
f1 (ppm)

/Volumes/Work ...W0041_5621.raw/ Injection 1 PDA - Total Absorbance Chromatogram

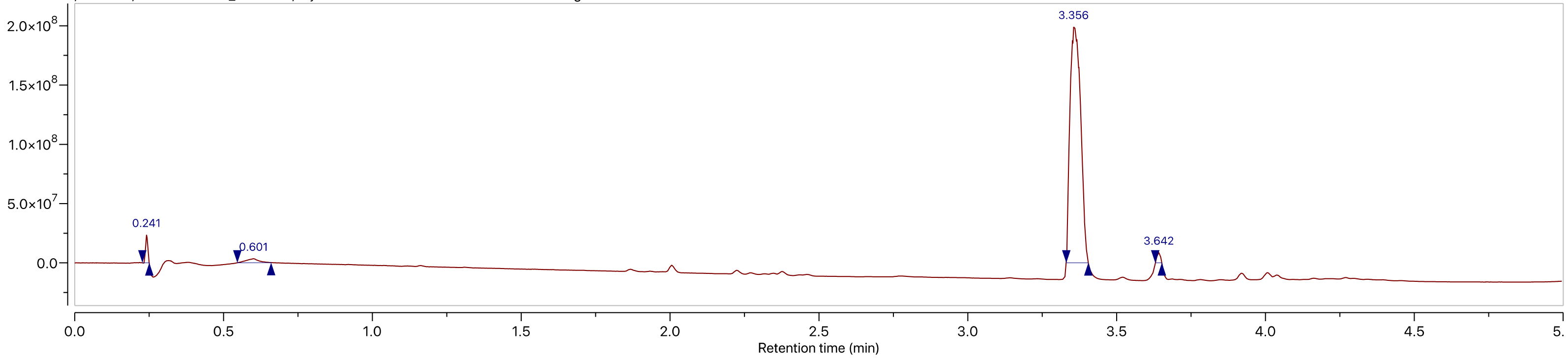

/Volumes/Work ...W0041_5621.raw/ Injection 1 MS ES+ TIC

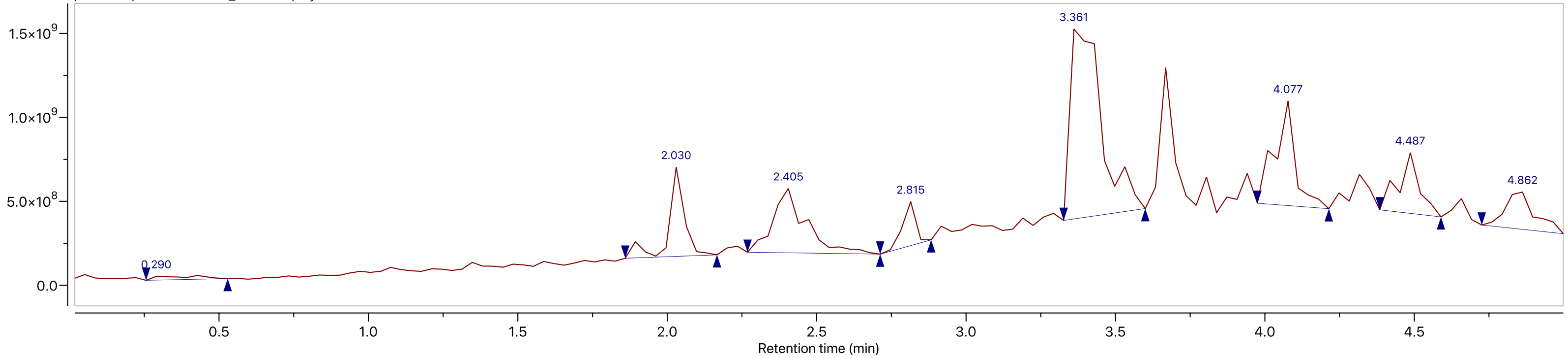

/Volumes/Work ...W0041_5621.raw/ Injection 1 MS ES+ MS + spectrum 3.36

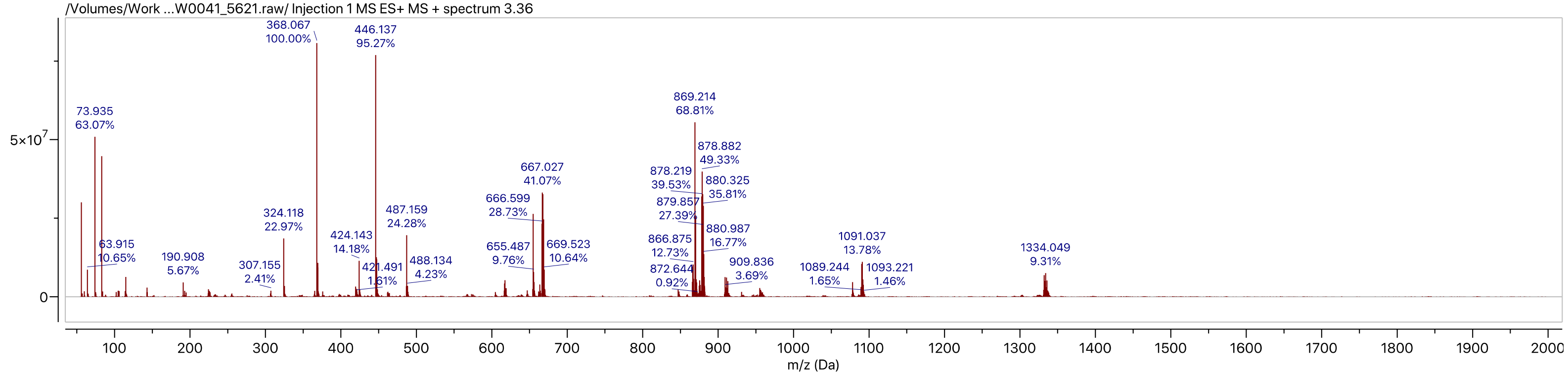

Compound 16b

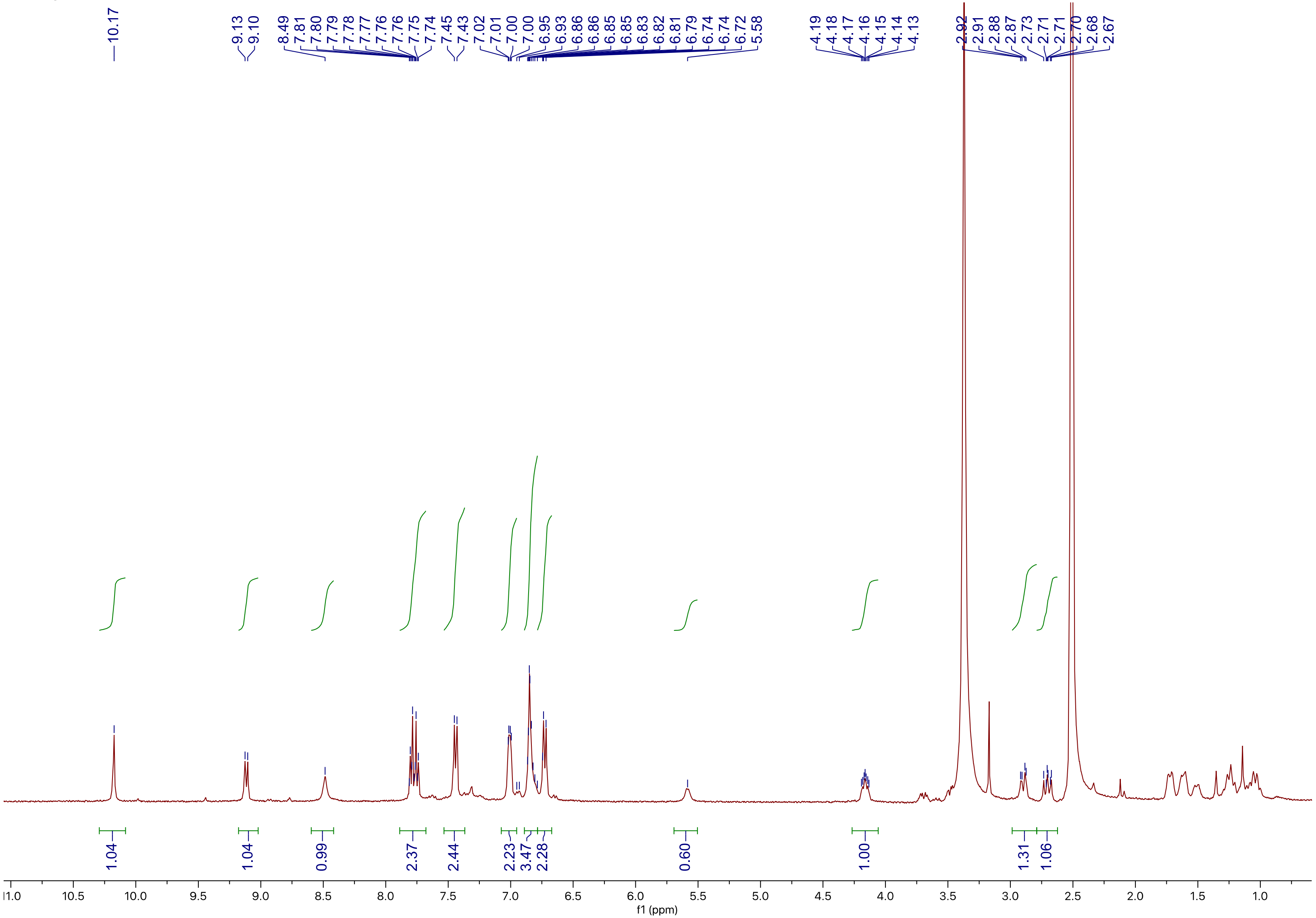

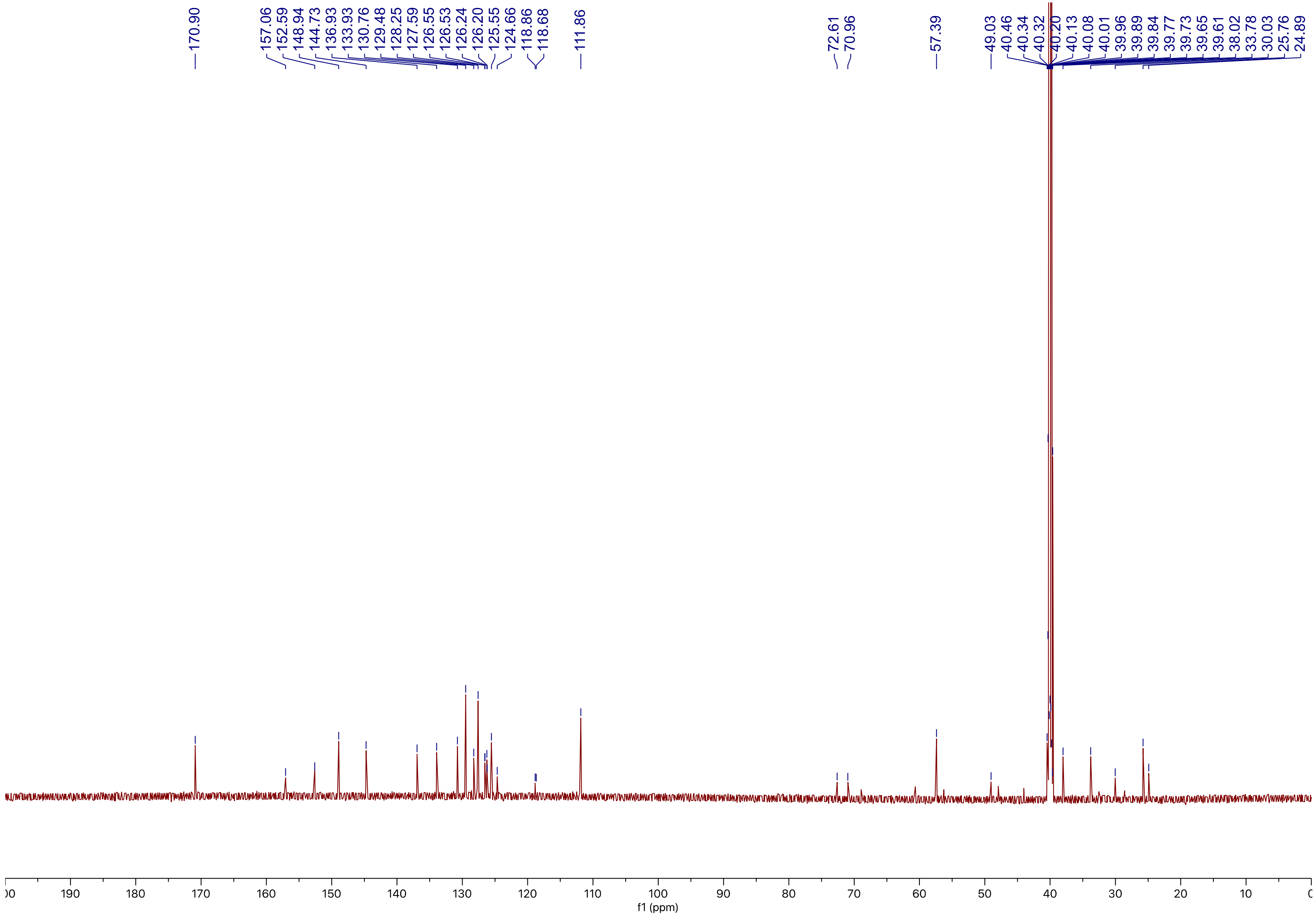
170.90
157.06
152.59
148.94
144.73
136.93
133.93
130.76
129.48
128.25
127.59
126.55
126.53
126.24
126.20
125.55
124.66
118.86
118.68
111.86
72.61
70.96
57.39
49.03
40.46
40.34
40.32
40.20
40.13
40.08
40.01
39.96
39.89
39.84
39.77
39.73
39.65
39.61
38.02
33.78
30.03
25.76
24.89
f1 (ppm)

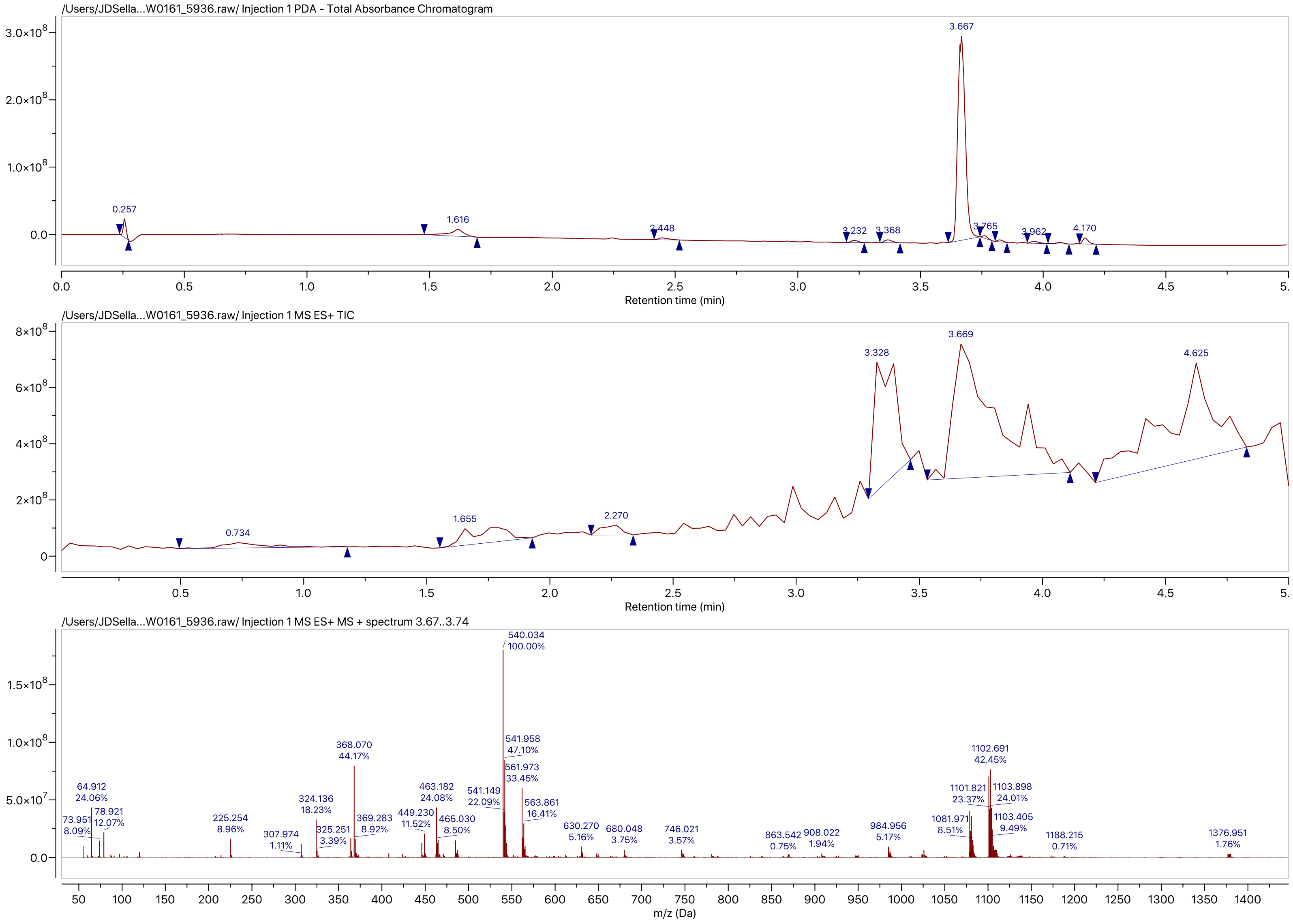
/Users/JDSella...W0161_5936.raw/ Injection 1 PDA - Total Absorbance Chromatogram
Retention time (min)
/Users/JDSella...W0161_5936.raw/ Injection 1 MS ES+ TIC
Retention time (min)
/Users/JDSella...W0161_5936.raw/ Injection 1 MS ES+ MS + spectrum 3.67..3.74
m/z (Da)
540.034 100.00%
541.958 47.10%
368.070 44.17%
1102.691 42.45%
561.973 33.45%

Compound 17a

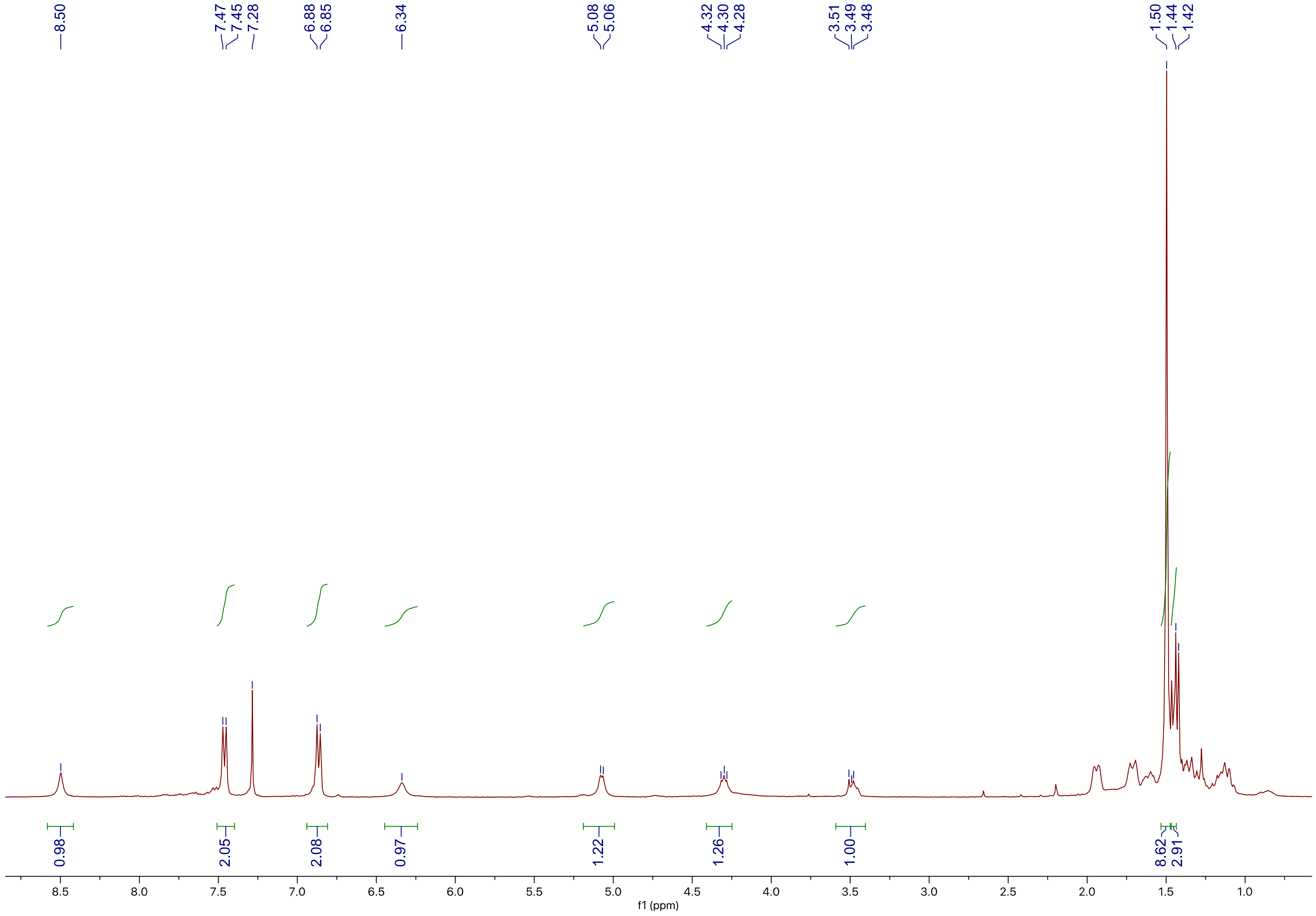

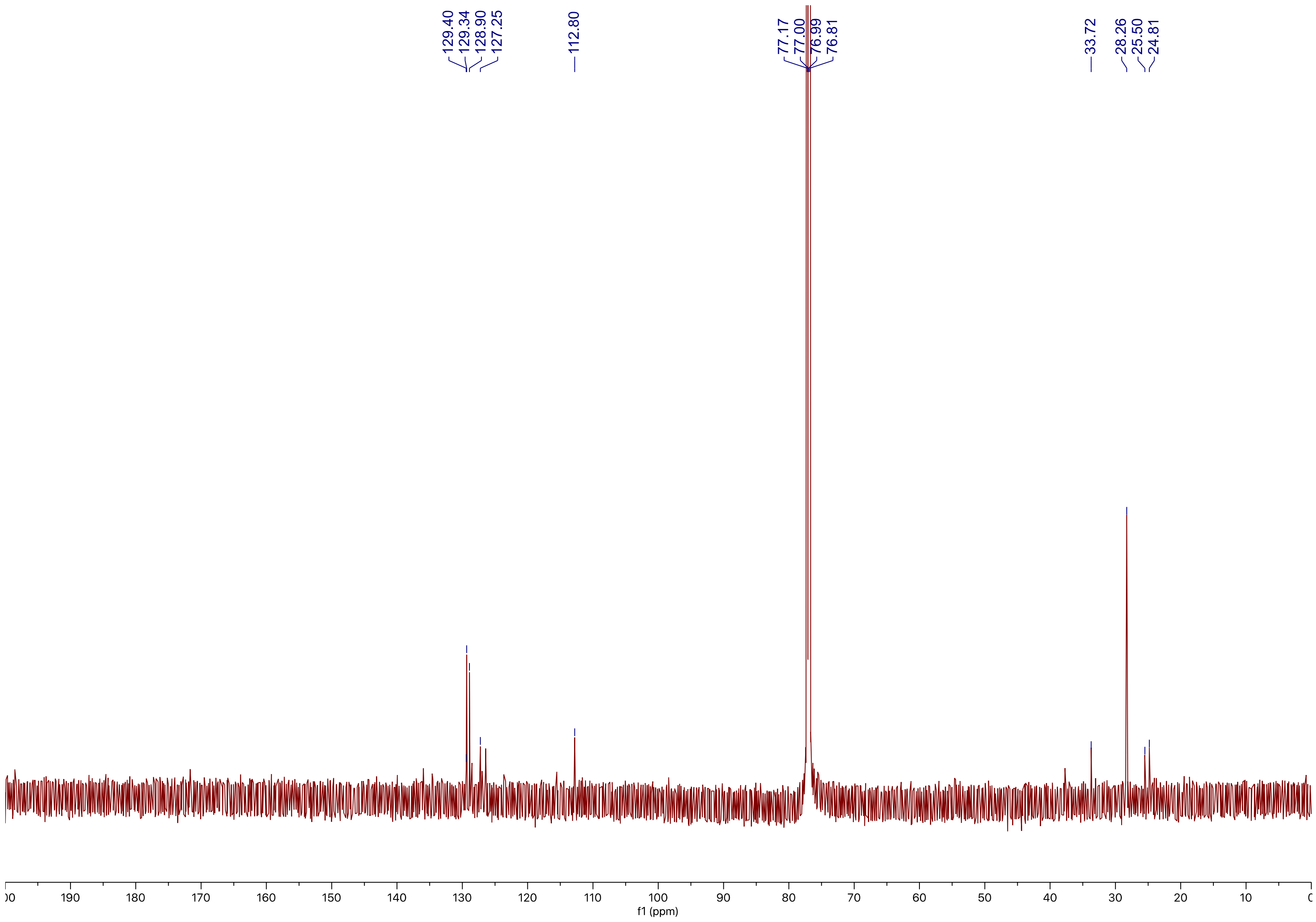
129.40
129.34
128.90
127.25
112.80
77.17
77.00
76.99
76.81
33.72
28.26
25.50
24.81
190 180 170 160 150 140 130 120 110 100 90 80 70 60 50 40 30 20 10
f1 (ppm)

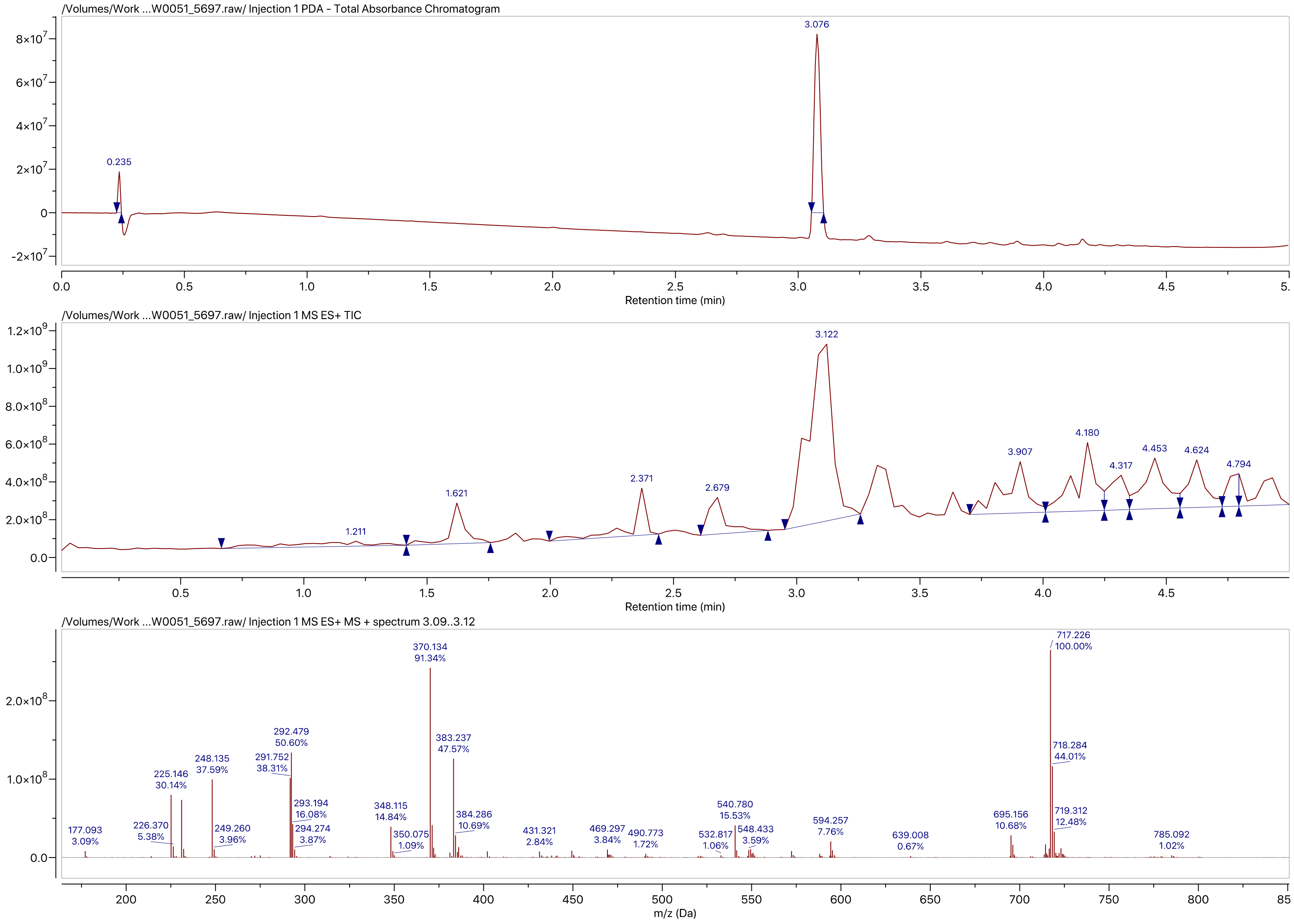
/Volumes/Work ...W0051_5697.raw/ Injection 1 PDA - Total Absorbance Chromatogram
3.076
0.235
Retention time (min)
/Volumes/Work ...W0051_5697.raw/ Injection 1 MS ES+ TIC
3.122
1.621
1.211
2.371
2.679
3.907
4.180
4.317
4.453
4.624
4.794
Retention time (min)
/Volumes/Work ...W0051_5697.raw/ Injection 1 MS ES+ MS + spectrum 3.09..3.12
717.226 100.00%
370.134 91.34%
292.479 50.60%
383.237 47.57%
718.284 44.01%
291.752 38.31%
248.135 37.59%
225.146 30.14%
293.194 16.08%
540.780 15.53%
348.115 14.84%
719.312 12.48%
384.286 10.69%
695.156 10.68%
594.257 7.76%
226.370 5.38%
249.260 3.96%
294.274 3.87%
469.297 3.84%
548.433 3.59%
177.093 3.09%
431.321 2.84%
490.773 1.72%
350.075 1.09%
532.817 1.06%
785.092 1.02%
639.008 0.67%
m/z (Da)

Compound 17b

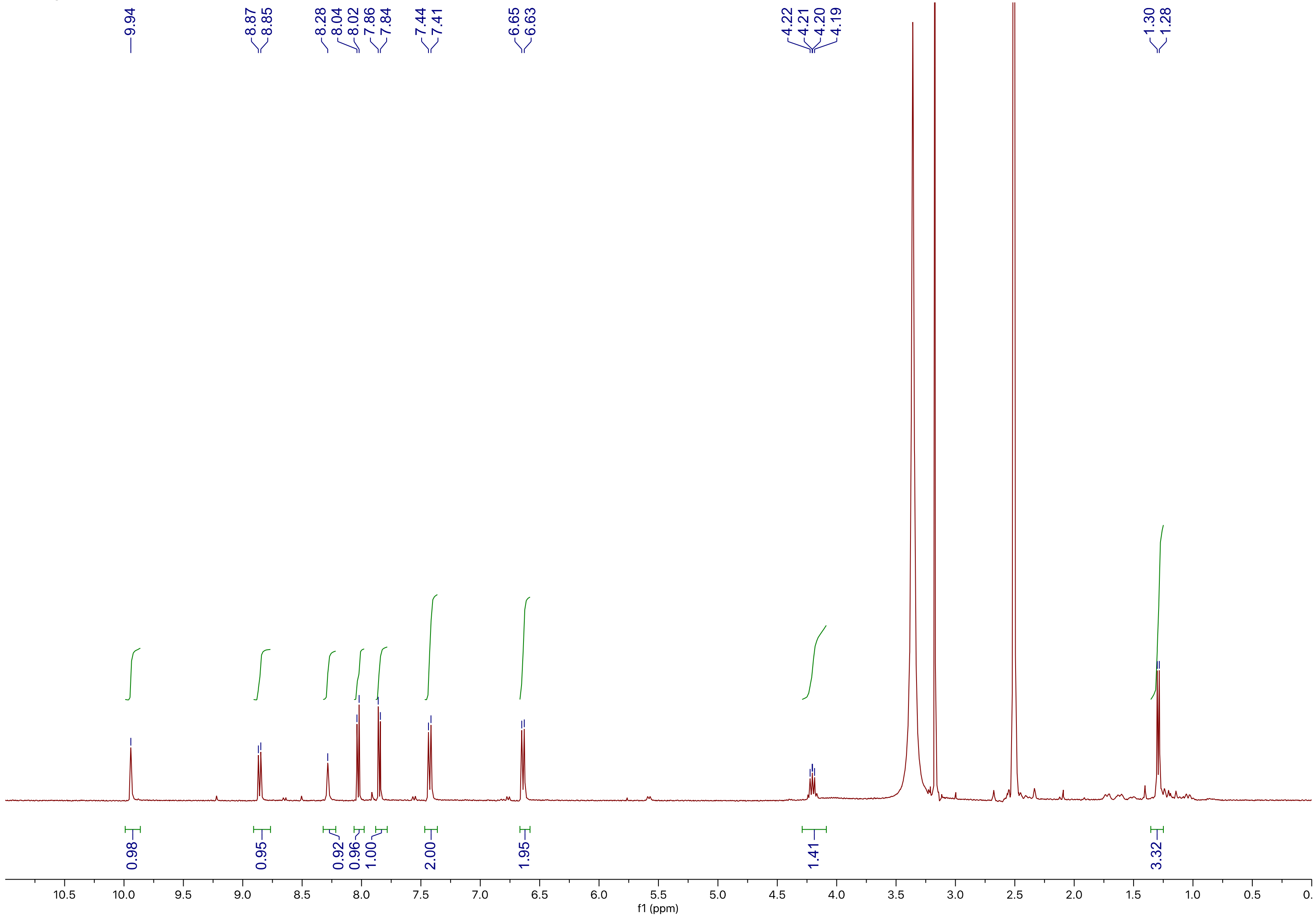

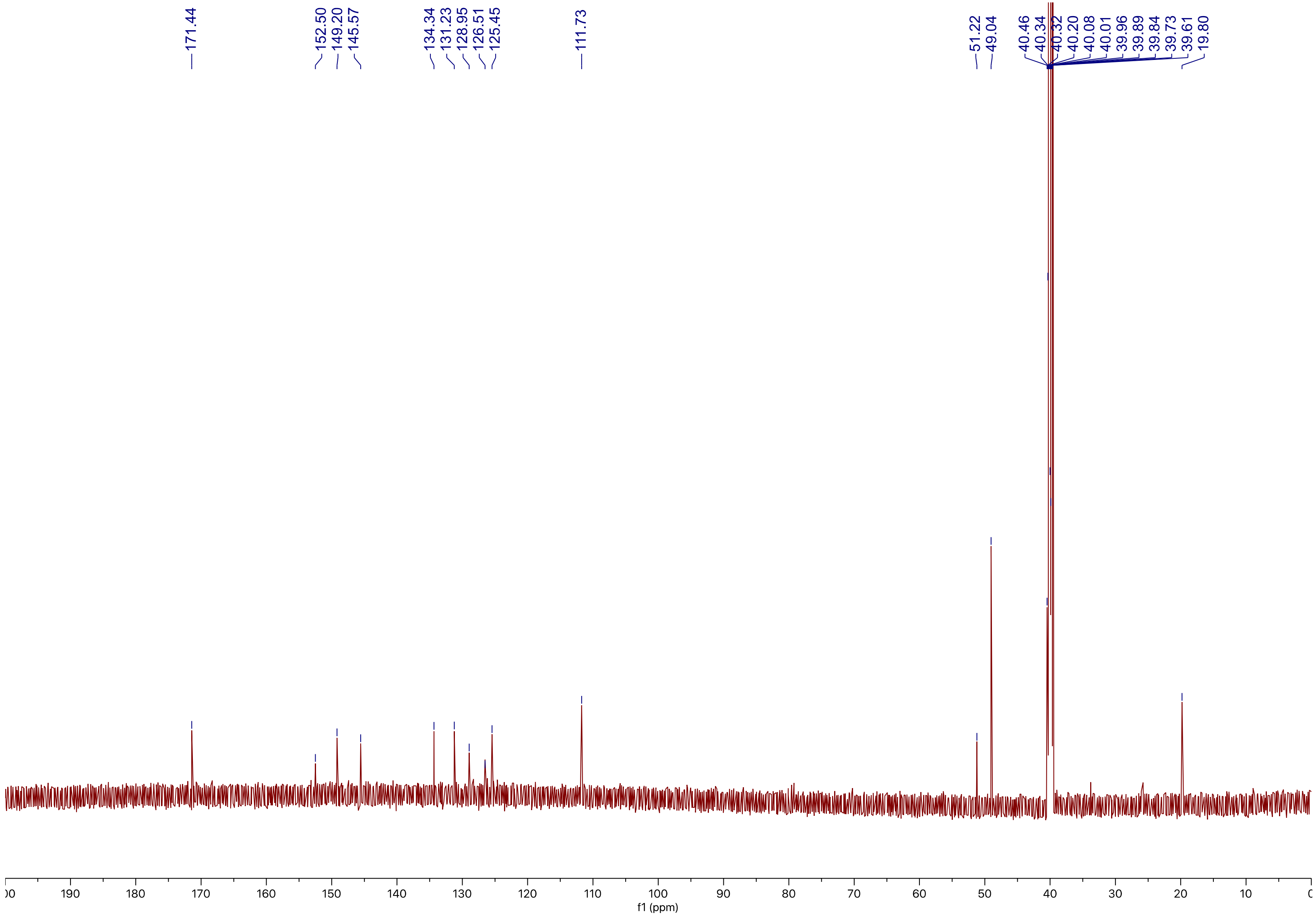
171.44
152.50
149.20
145.57
134.34
131.23
128.95
126.51
125.45
111.73
51.22
49.04
40.46
40.34
40.32
40.20
40.08
40.01
39.96
39.89
39.84
39.73
39.61
19.80
f1 (ppm)

/Users/JDSella...W0171_5932.raw/ Injection 1 PDA - Total Absorbance Chromatogram

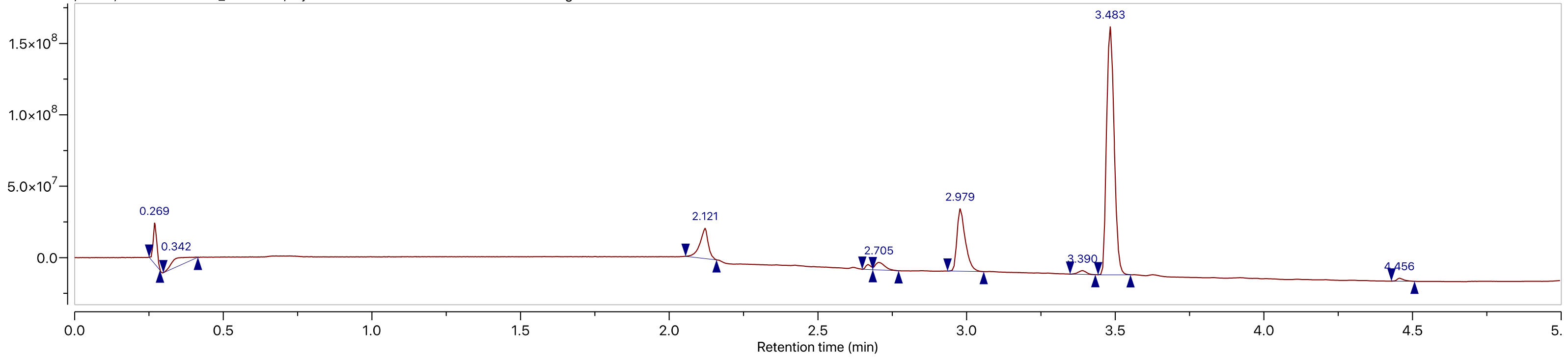

/Users/JDSella...W0171_5932.raw/ Injection 1 MS ES+ TIC

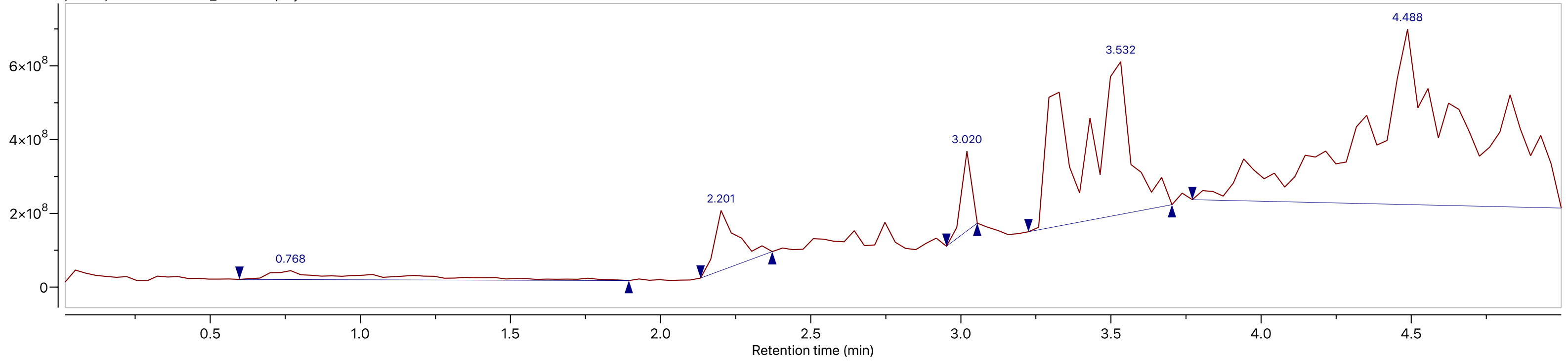

/Users/JDSella...W0171_5932.raw/ Injection 1 MS ES+ MS + spectrum 3.53

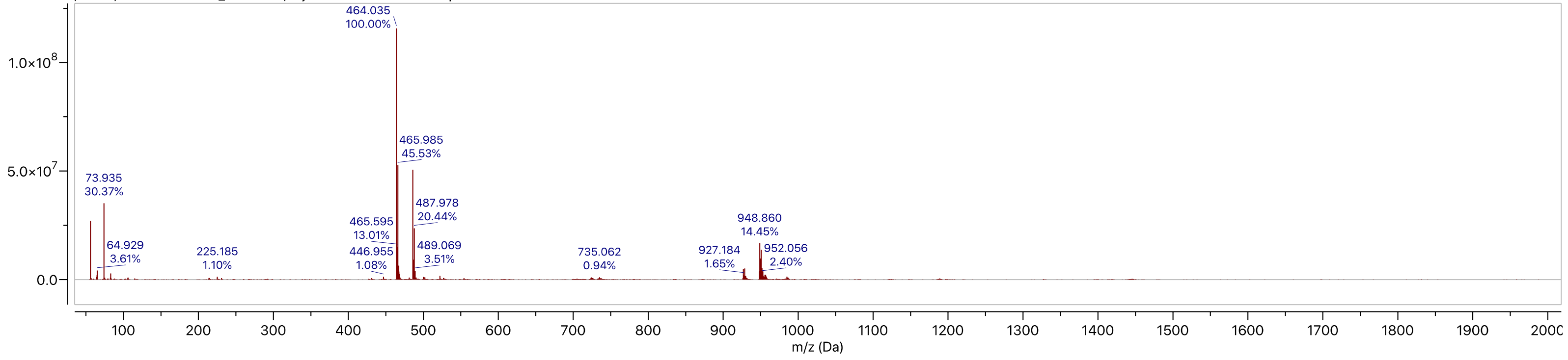

Compound 18a

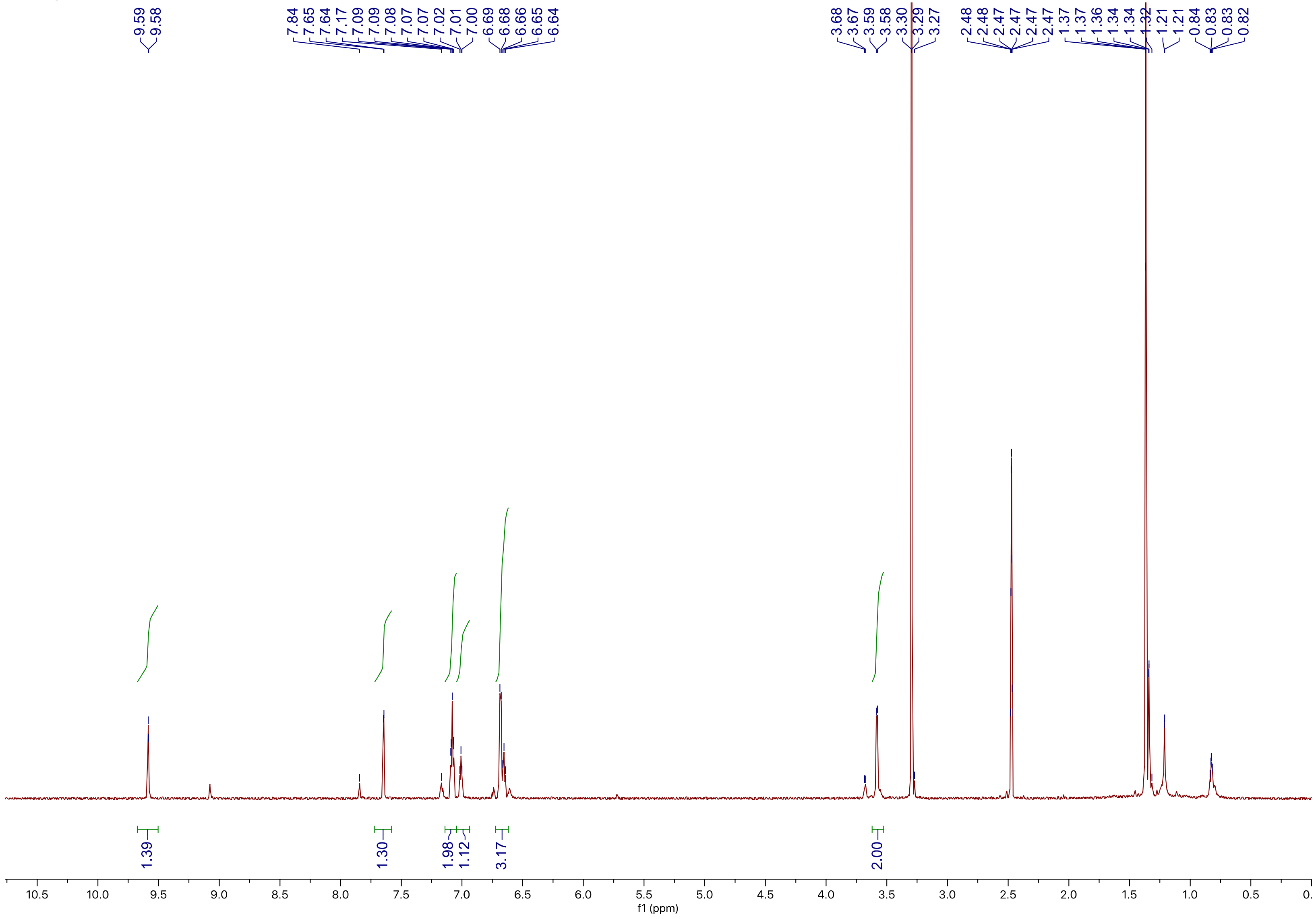

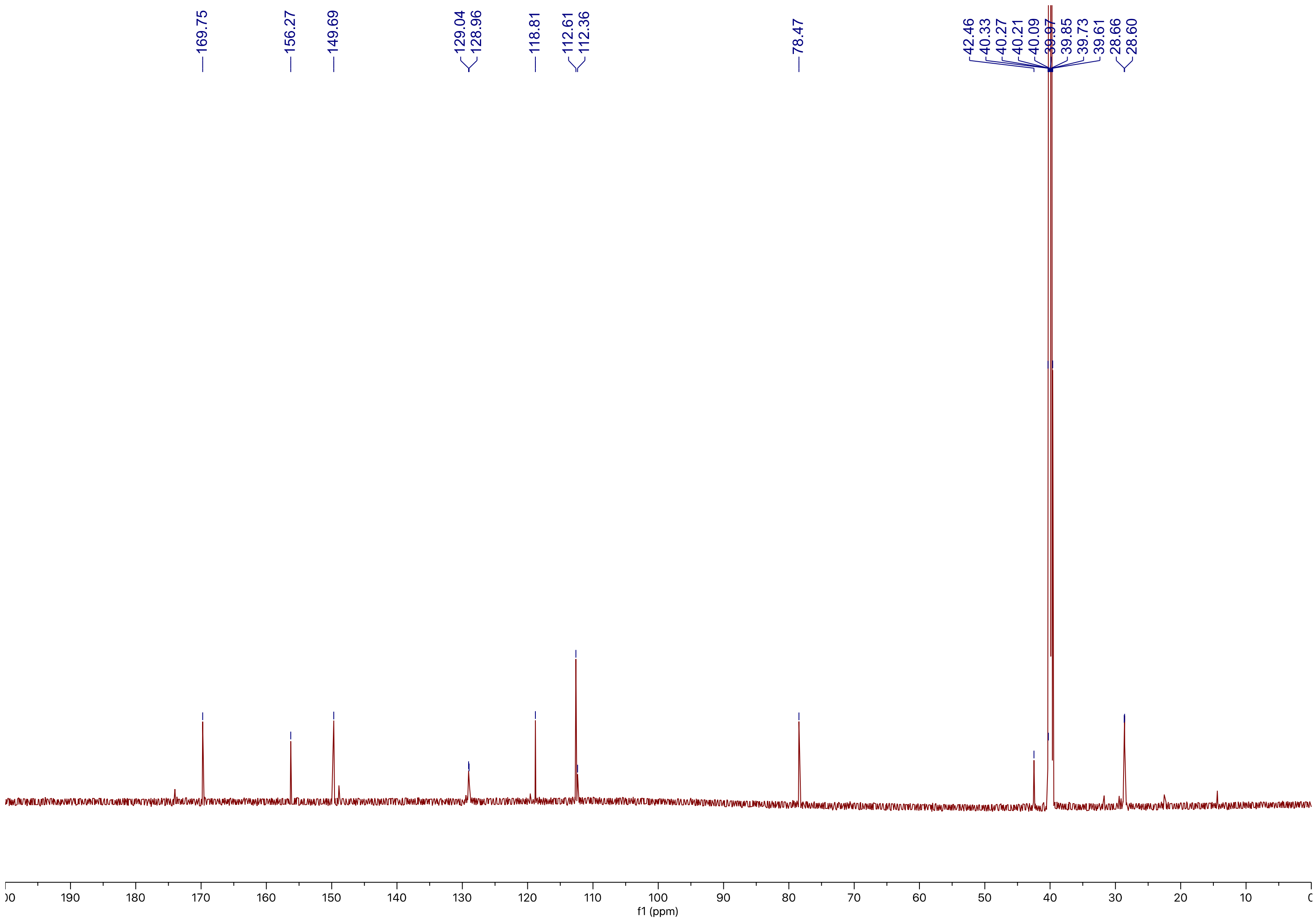
169.75
156.27
149.69
129.04
128.96
118.81
112.61
112.36
78.47
42.46
40.33
40.27
40.21
40.09
39.97
39.85
39.73
39.61
28.66
28.60
190
180
170
160
150
140
130
120
110
100
90
80
70
60
50
40
30
20
10
f1 (ppm)

Compound 19a

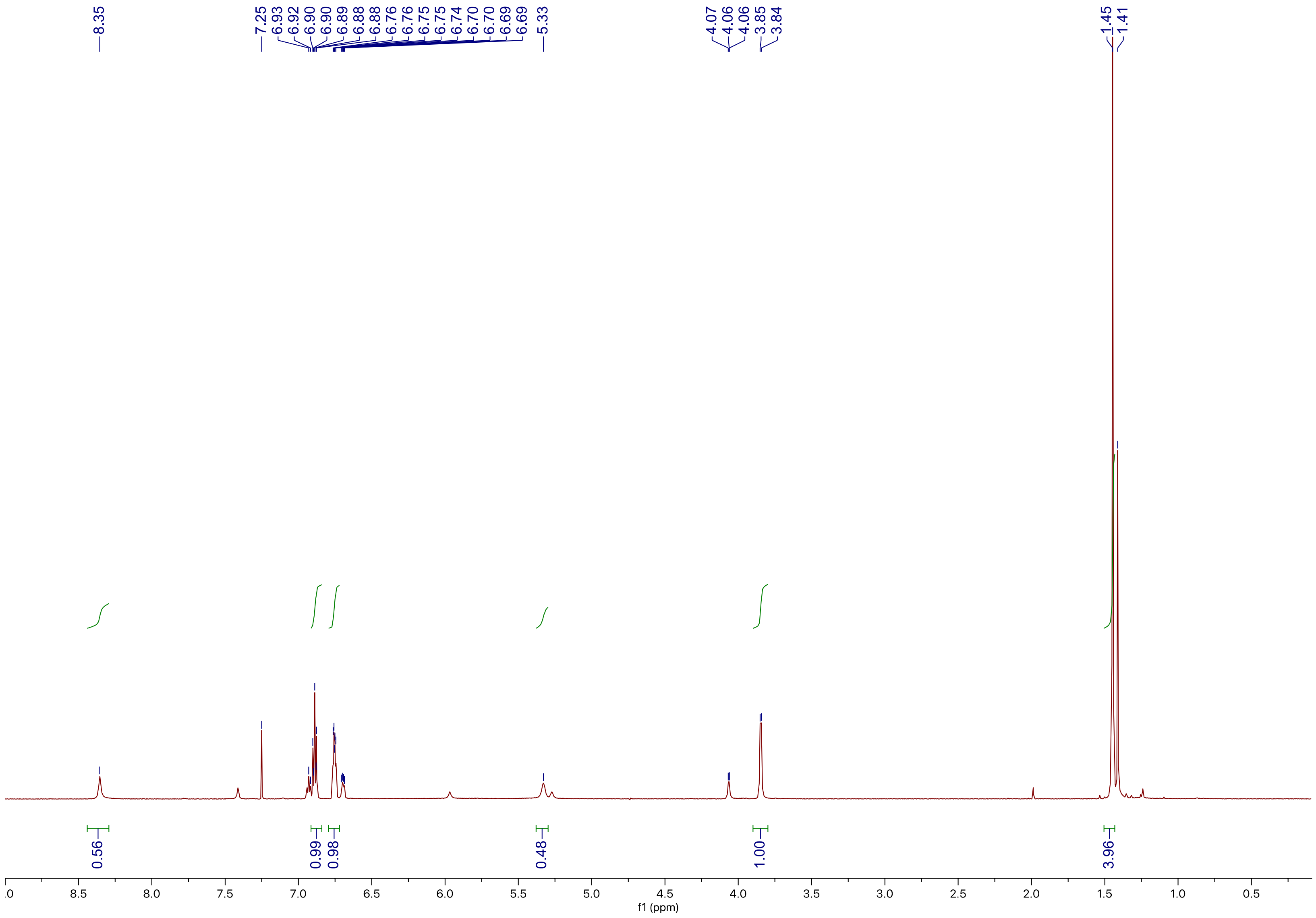

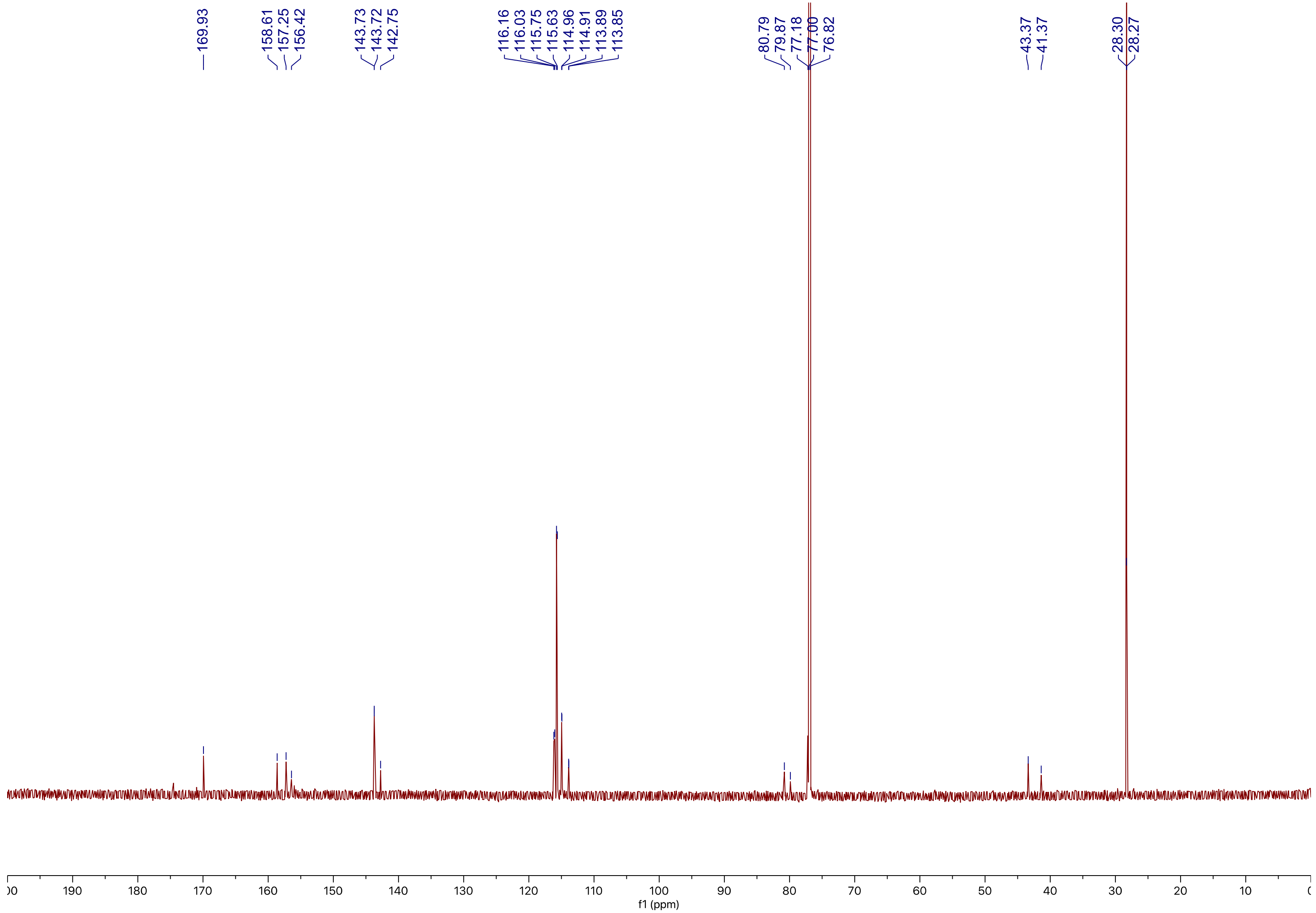
169.93
158.61
157.25
156.42
143.73
143.72
142.75
116.16
116.03
115.75
115.63
114.96
114.91
113.89
113.85
80.79
79.87
77.18
77.00
76.82
43.37
41.37
28.30
28.27
f1 (ppm)

/Users/JDSella...S0031_5626.raw/ Injection 1 PDA - Total Absorbance Chromatogram

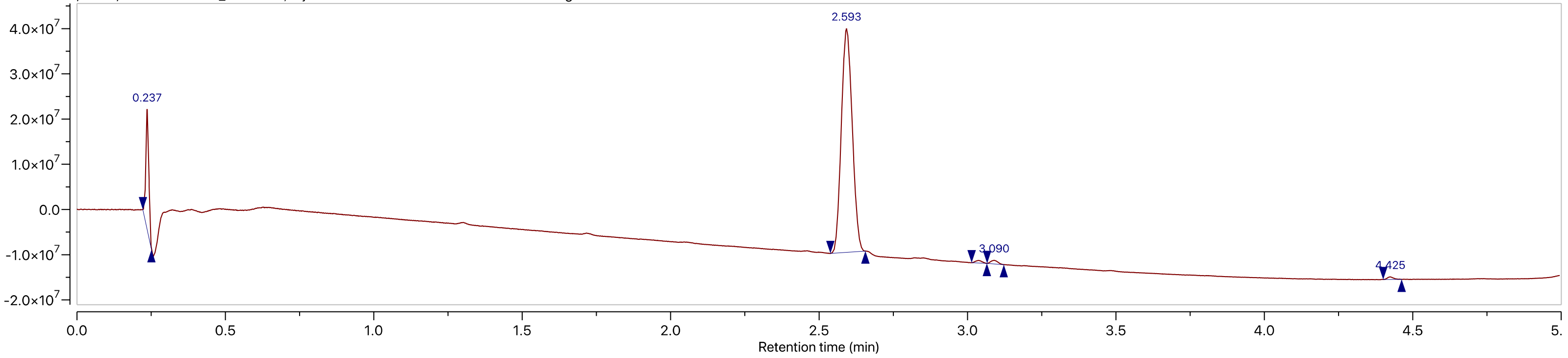

/Users/JDSella...S0031_5626.raw/ Injection 1 MS ES+ TIC

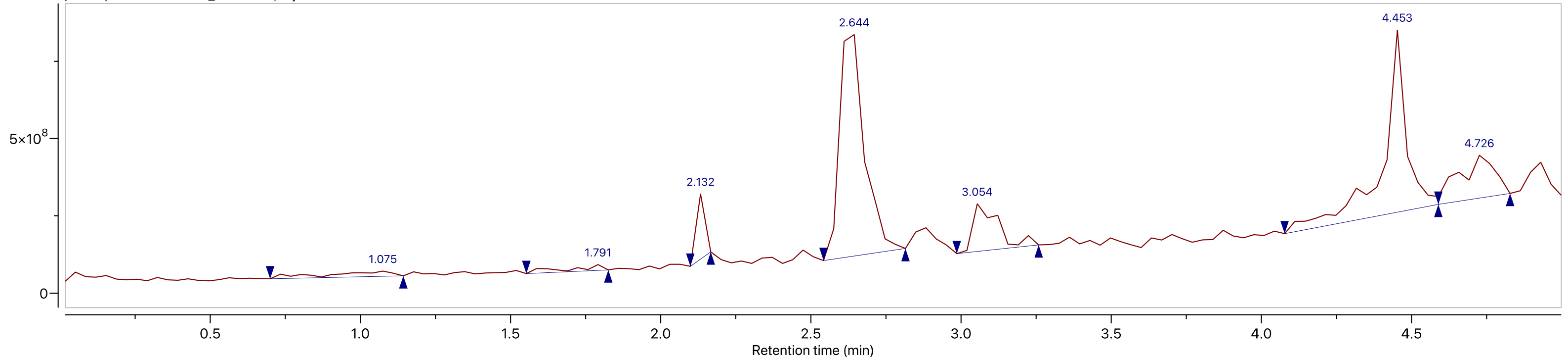

/Users/JDSella...S0031_5626.raw/ Injection 1 MS ES+ MS + spectrum 2.61..2.64

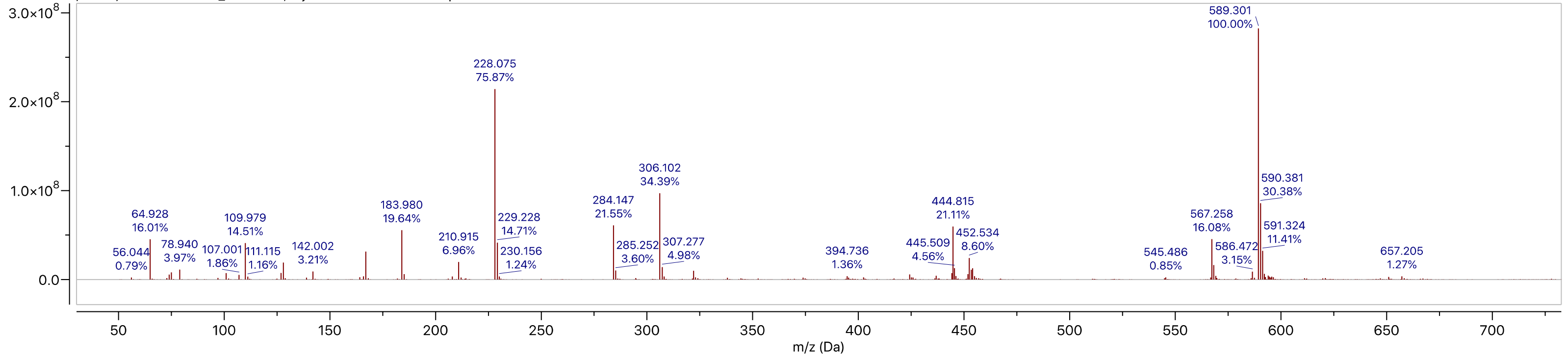

Compound 19b

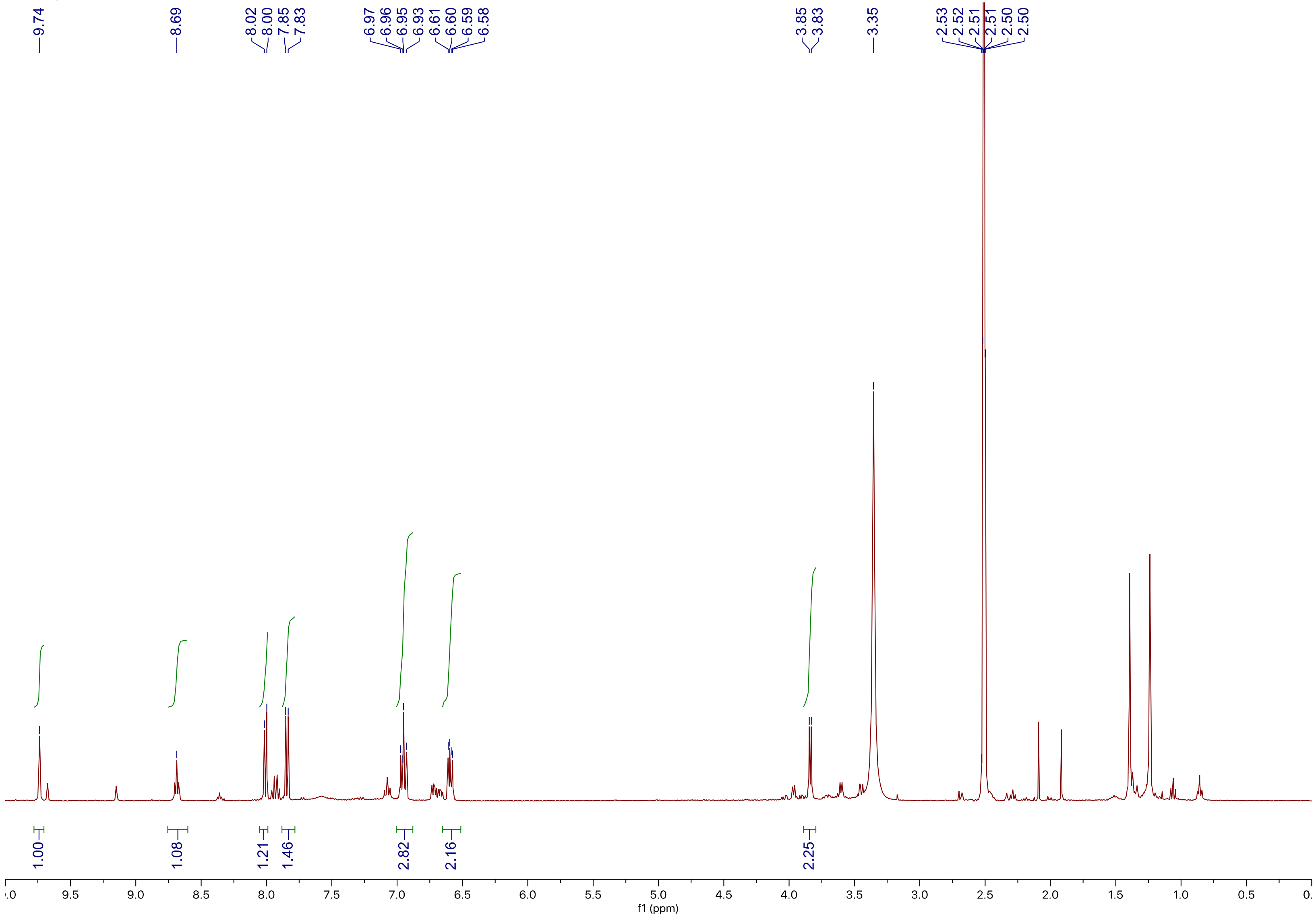

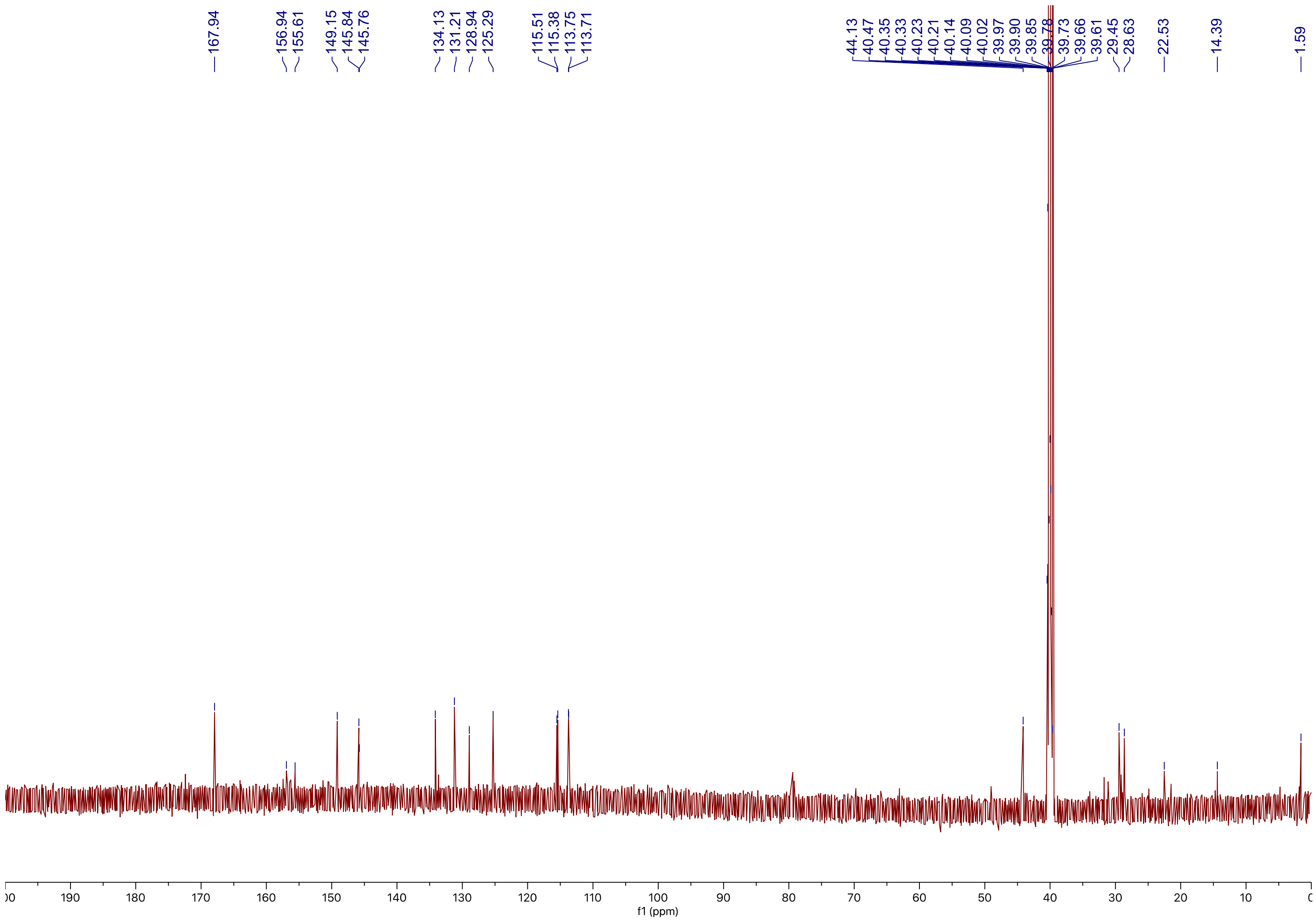

167.94
156.94
155.61
149.15
145.84
145.76
134.13
131.21
128.94
125.29
115.51
115.38
113.75
113.71
44.13
40.47
40.35
40.33
40.23
40.21
40.14
40.09
40.02
39.97
39.90
39.85
39.78
39.73
39.66
39.61
29.45
28.63
22.53
14.39
1.59
f1 (ppm)

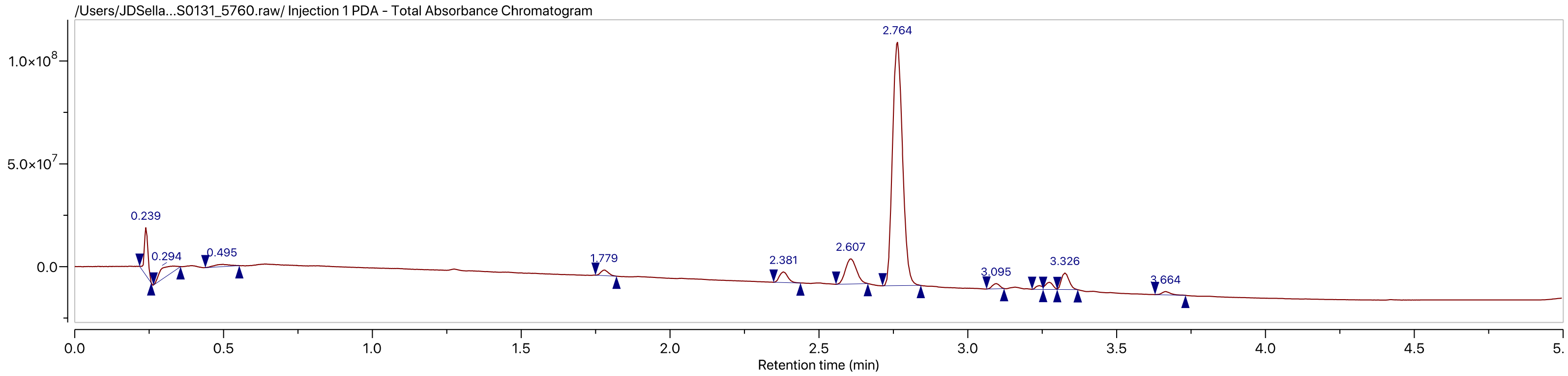

/Users/JDSella...S0131_5760.raw/ Injection 1 PDA - Total Absorbance Chromatogram
1.0×10^8
5.0×10^7
0.0
2.764
0.239
0.294
0.495
1.779
2.381
2.607
3.095
3.326
3.664
Retention time (min)

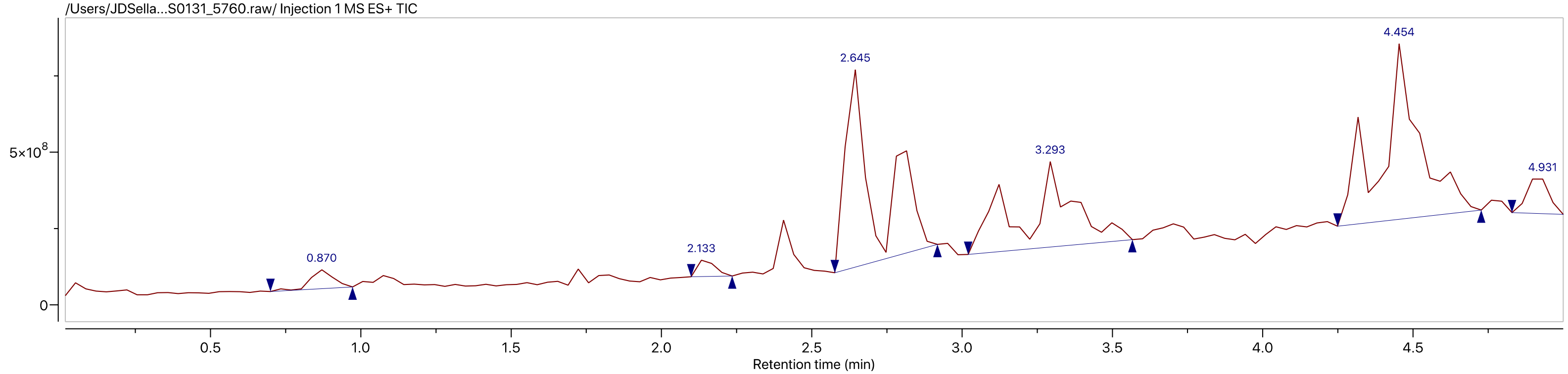

/Users/JDSella...S0131_5760.raw/ Injection 1 MS ES+ TIC
5×10^8
0
4.454
2.645
3.293
4.931
2.133
0.870
Retention time (min)

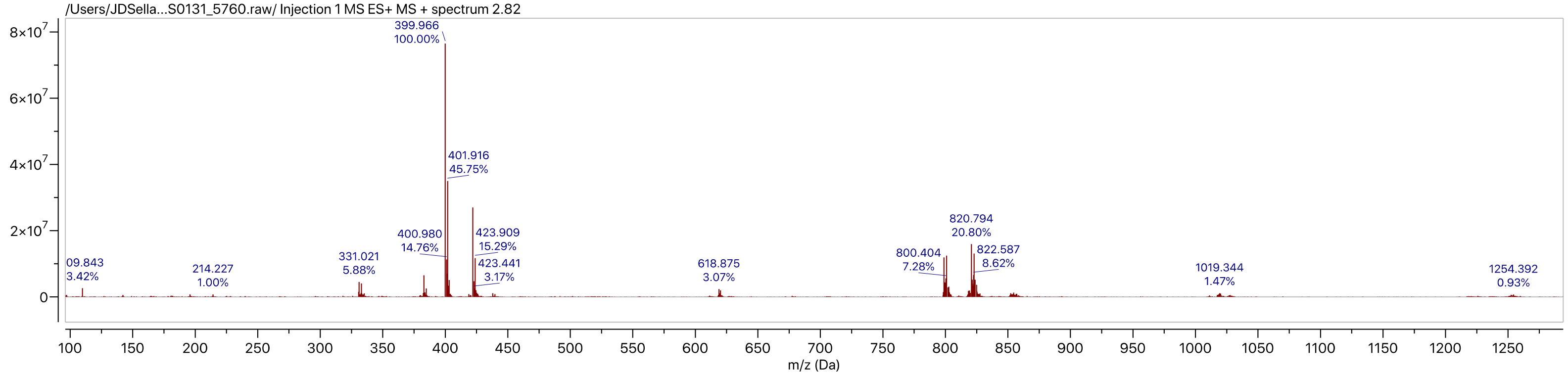

/Users/JDSella...S0131_5760.raw/ Injection 1 MS ES+ MS + spectrum 2.82
8×10^7
6×10^7
4×10^7
2×10^7
0
399.966
100.00%
401.916
45.75%
400.980
14.76%
423.909
15.29%
423.441
3.17%
331.021
5.88%
09.843
3.42%
214.227
1.00%
618.875
3.07%
800.404
7.28%
820.794
20.80%
822.587
8.62%
1019.344
1.47%
1254.392
0.93%
m/z (Da)

Compound 20a

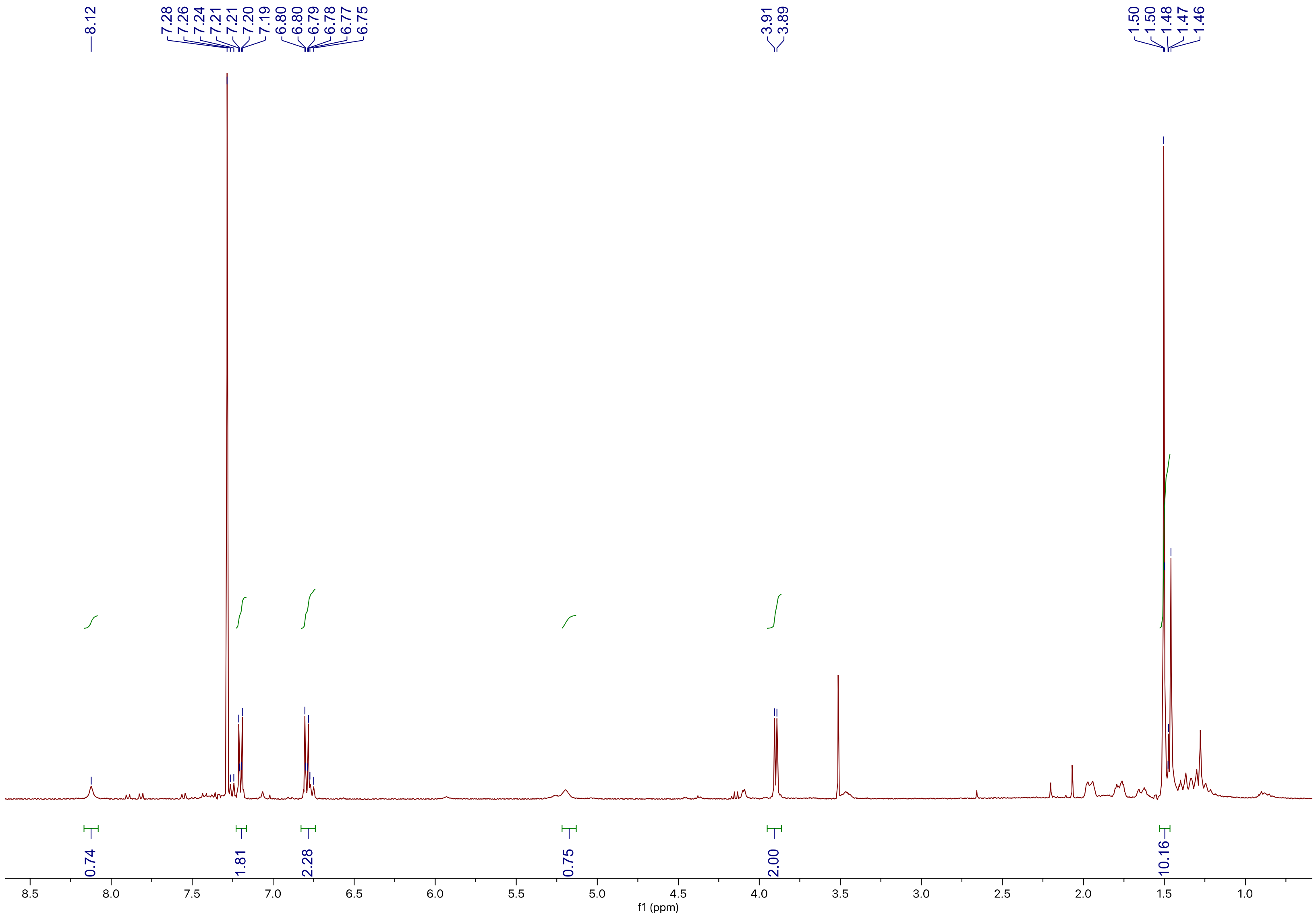

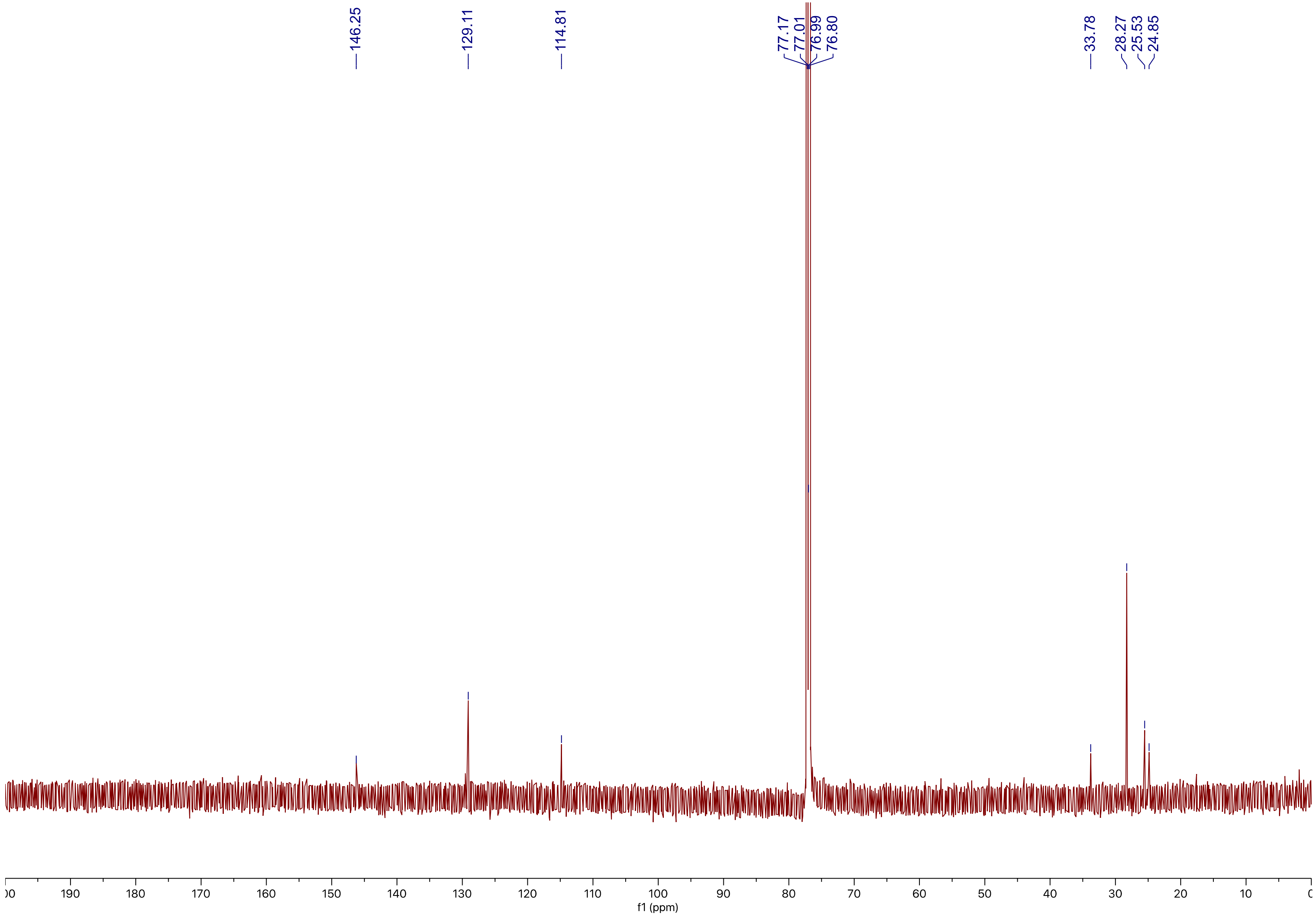

146.25
129.11
114.81
77.17
77.01
76.99
76.80
33.78
28.27
25.53
24.85
200
190
180
170
160
150
140
130
120
110
100
90
80
70
60
50
40
30
20
10
0
f1 (ppm)

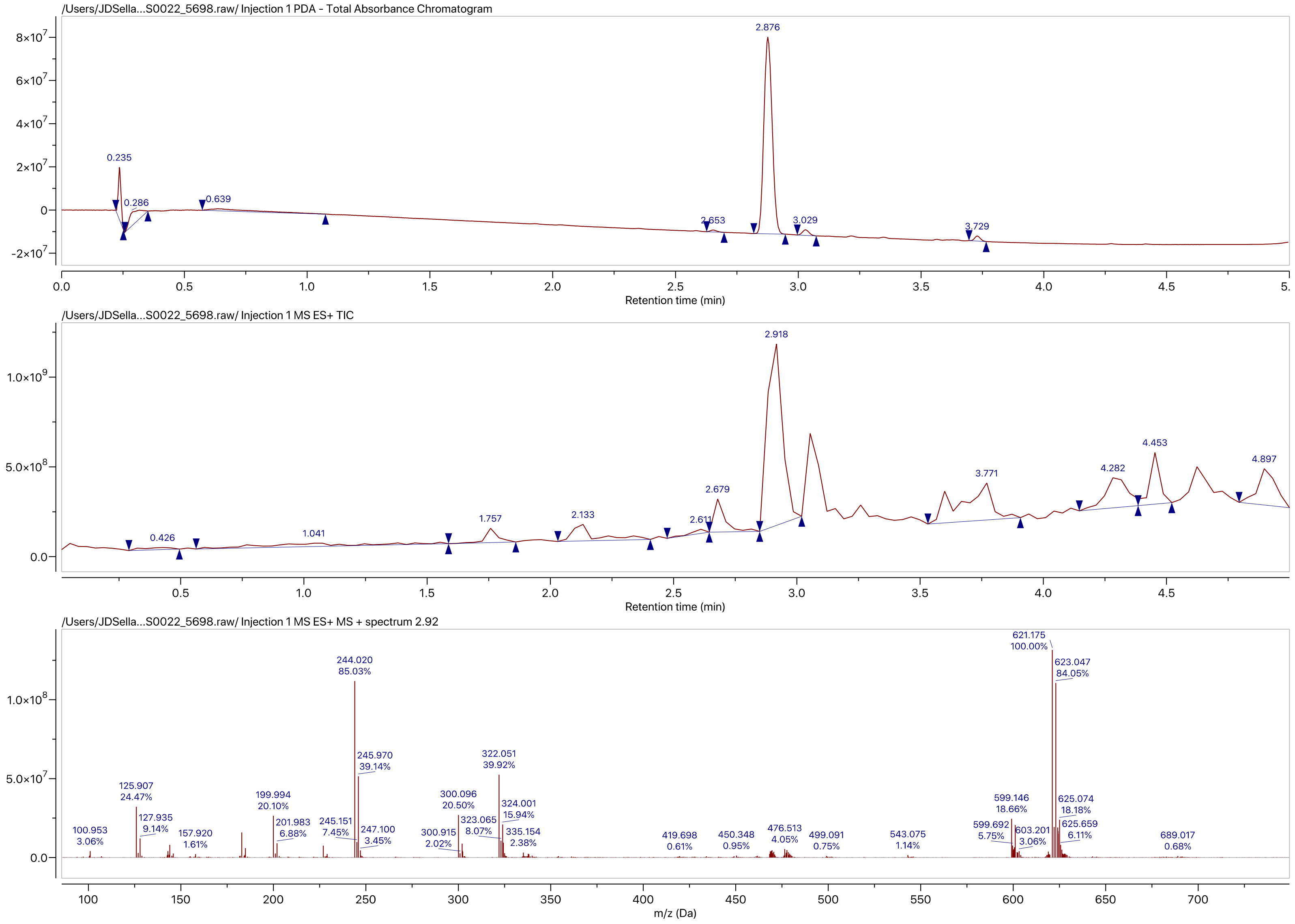
/Users/JDSella...S0022_5698.raw/ Injection 1 PDA - Total Absorbance Chromatogram
0.235
0.286
0.639
2.653
2.876
3.029
3.729
Retention time (min)
/Users/JDSella...S0022_5698.raw/ Injection 1 MS ES+ TIC
0.426
1.041
1.757
2.133
2.611
2.679
2.918
3.771
4.282
4.453
4.897
Retention time (min)
/Users/JDSella...S0022_5698.raw/ Injection 1 MS ES+ MS + spectrum 2.92
100.953 3.06%
125.907 24.47%
127.935 9.14%
157.920 1.61%
199.994 20.10%
201.983 6.88%
244.020 85.03%
245.151 7.45%
245.970 39.14%
247.100 3.45%
300.096 20.50%
300.915 2.02%
322.051 39.92%
323.065 8.07%
324.001 15.94%
335.154 2.38%
419.698 0.61%
450.348 0.95%
476.513 4.05%
499.091 0.75%
543.075 1.14%
599.146 18.66%
599.692 5.75%
603.201 3.06%
621.175 100.00%
623.047 84.05%
625.074 18.18%
625.659 6.11%
689.017 0.68%
m/z (Da)

Compound 20b

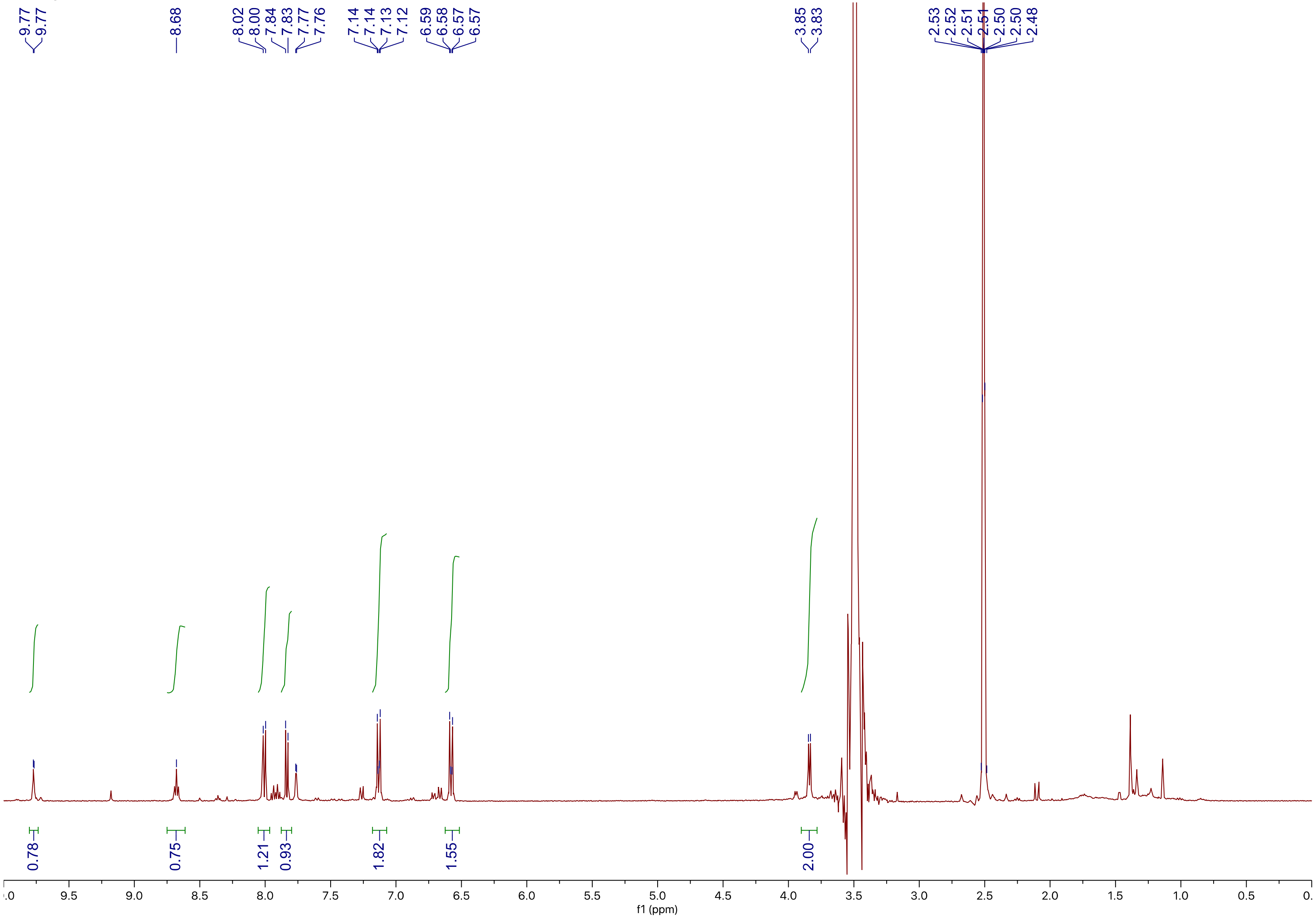

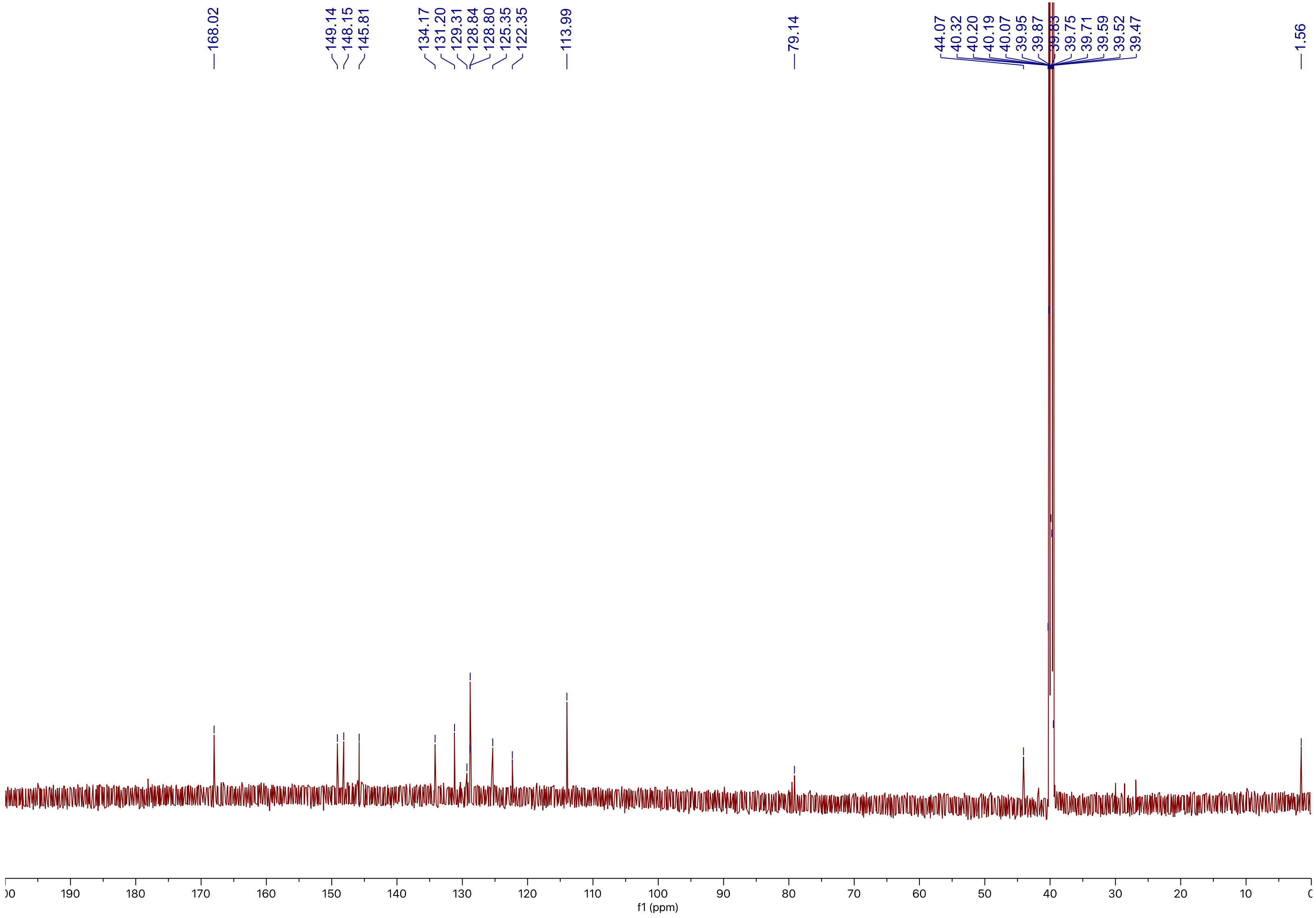

168.02
149.14
148.15
145.81
134.17
131.20
129.31
128.84
128.80
125.35
122.35
113.99
79.14
44.07
40.32
40.20
40.19
40.07
39.95
39.87
39.83
39.75
39.71
39.59
39.52
39.47
1.56
190
180
170
160
150
140
130
120
110
100
90
80
70
60
50
40
30
20
10
f1 (ppm)

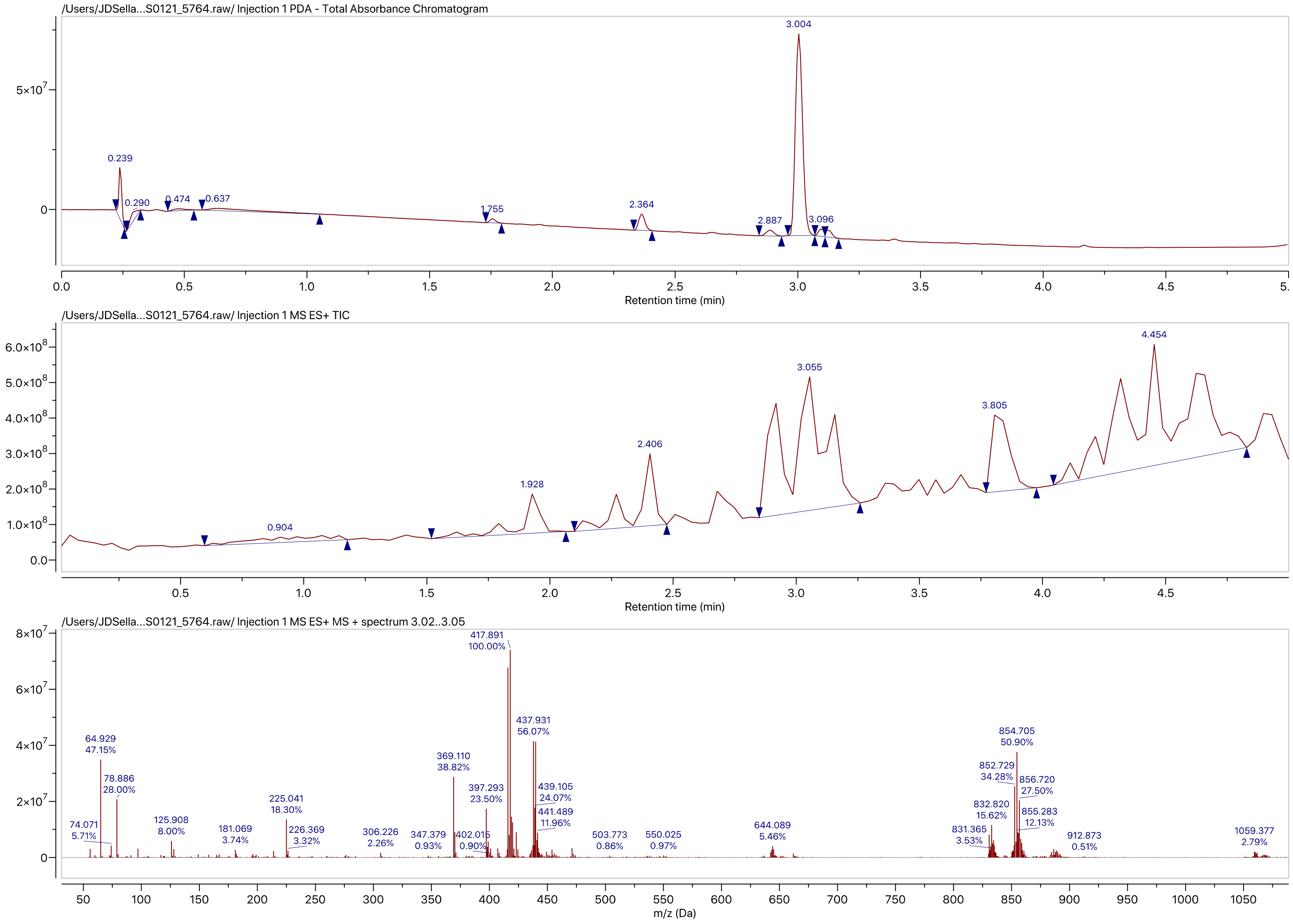
/Users/JDSella...S0121_5764.raw/ Injection 1 PDA - Total Absorbance Chromatogram
3.004
0.239
0.290
0.474
0.637
1.755
2.364
2.887
3.096
Retention time (min)
/Users/JDSella...S0121_5764.raw/ Injection 1 MS ES+ TIC
4.454
3.055
3.805
2.406
1.928
0.904
Retention time (min)
/Users/JDSella...S0121_5764.raw/ Injection 1 MS ES+ MS + spectrum 3.02..3.05
417.891 100.00%
437.931 56.07%
854.705 50.90%
64.929 47.15%
369.110 38.82%
852.729 34.28%
78.886 28.00%
856.720 27.50%
439.105 24.07%
397.293 23.50%
225.041 18.30%
832.820 15.62%
855.283 12.13%
441.489 11.96%
125.908 8.00%
74.071 5.71%
644.089 5.46%
181.069 3.74%
831.365 3.53%
226.369 3.32%
1059.377 2.79%
306.226 2.26%
550.025 0.97%
347.379 0.93%
402.015 0.90%
503.773 0.86%
912.873 0.51%
m/z (Da)

Compound 21a

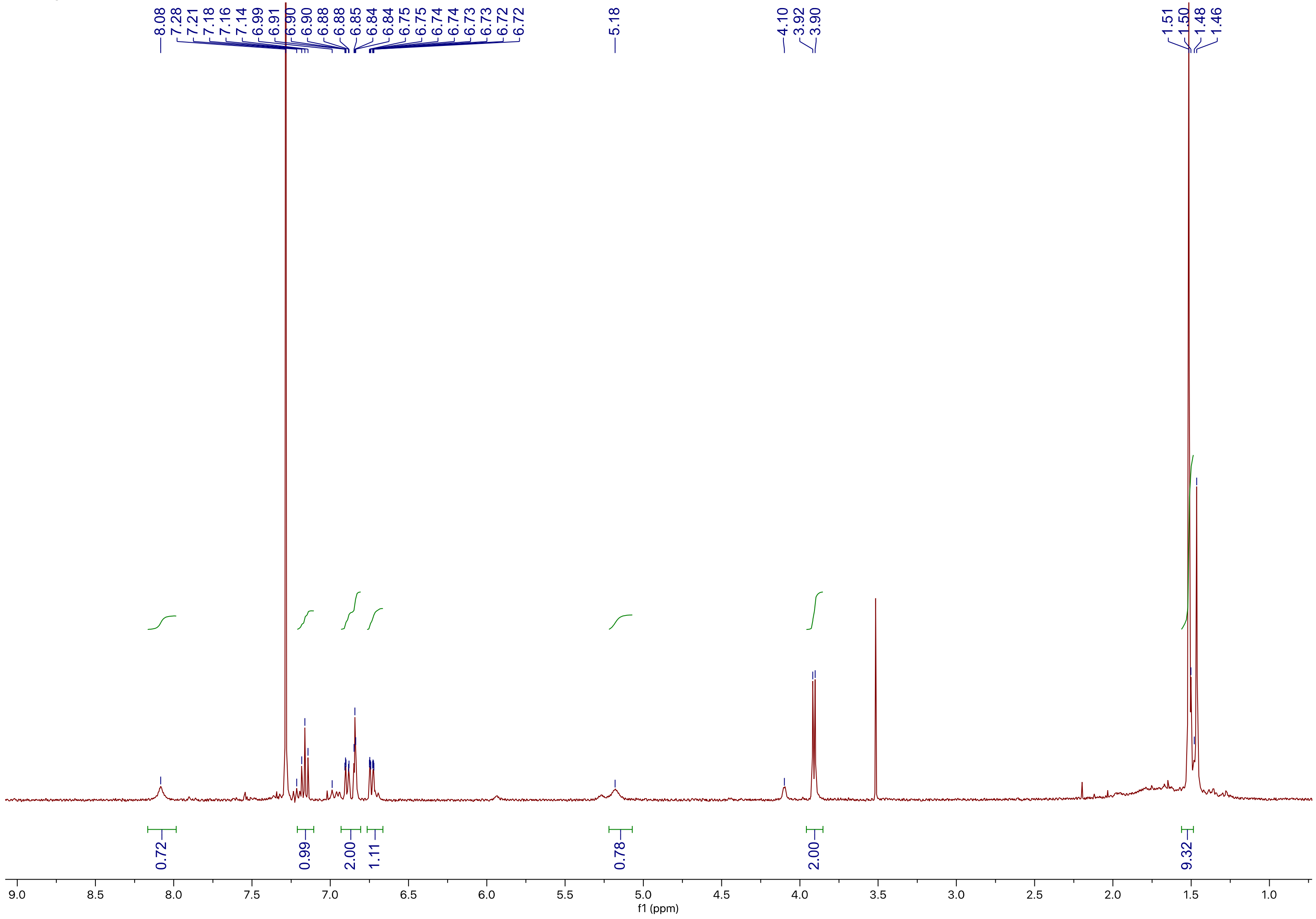

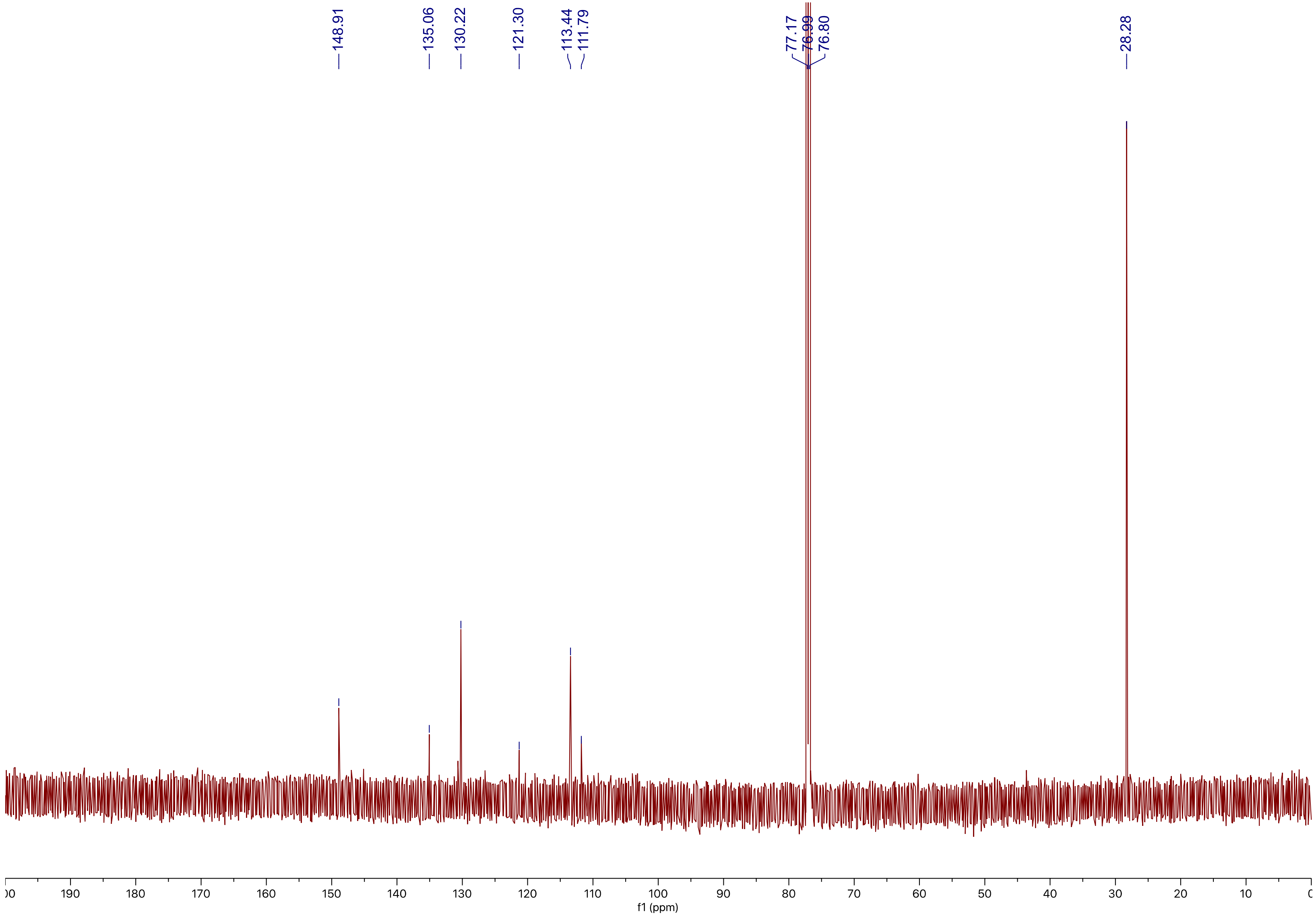
148.91
135.06
130.22
121.30
113.44
111.79
77.17
76.99
76.80
28.28
00
190
180
170
160
150
140
130
120
110
100
90
80
70
60
50
40
30
20
10
0
f1 (ppm)

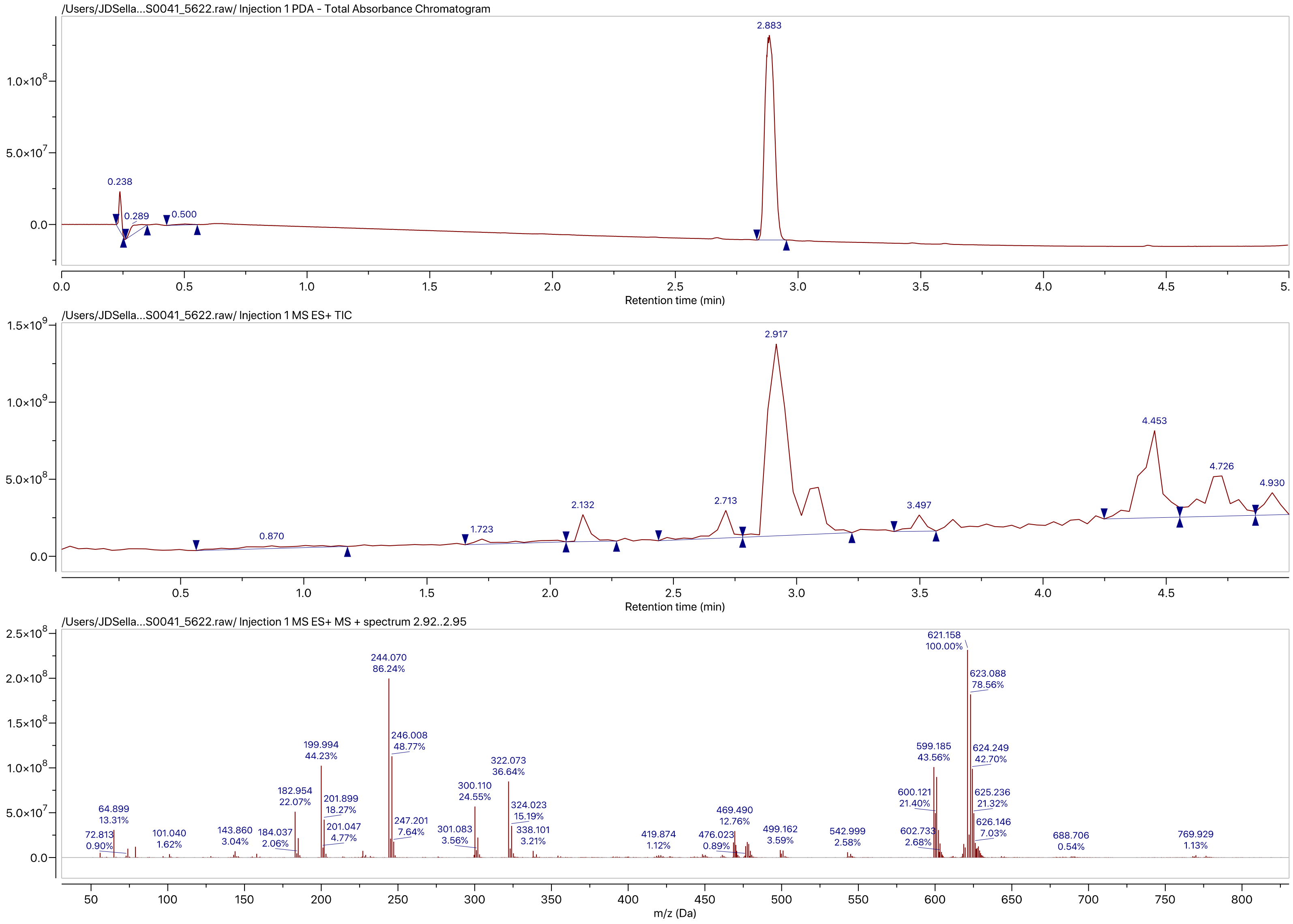

/Users/JDSella...S0041_5622.raw/ Injection 1 PDA - Total Absorbance Chromatogram
2.883
0.238
0.289
0.500
Retention time (min)
/Users/JDSella...S0041_5622.raw/ Injection 1 MS ES+ TIC
2.917
4.453
4.726
4.930
2.132
2.713
3.497
1.723
0.870
Retention time (min)
/Users/JDSella...S0041_5622.raw/ Injection 1 MS ES+ MS + spectrum 2.92..2.95
621.158 100.00%
623.088 78.56%
244.070 86.24%
246.008 48.77%
199.994 44.23%
599.185 43.56%
624.249 42.70%
322.073 36.64%
300.110 24.55%
182.954 22.07%
201.899 18.27%
600.121 21.40%
625.236 21.32%
324.023 15.19%
469.490 12.76%
64.899 13.31%
626.146 7.03%
247.201 7.64%
201.047 4.77%
72.813 0.90%
101.040 1.62%
143.860 3.04%
184.037 2.06%
301.083 3.56%
338.101 3.21%
419.874 1.12%
476.023 0.89%
499.162 3.59%
542.999 2.58%
602.733 2.68%
688.706 0.54%
769.929 1.13%
m/z (Da)

Compound 21b

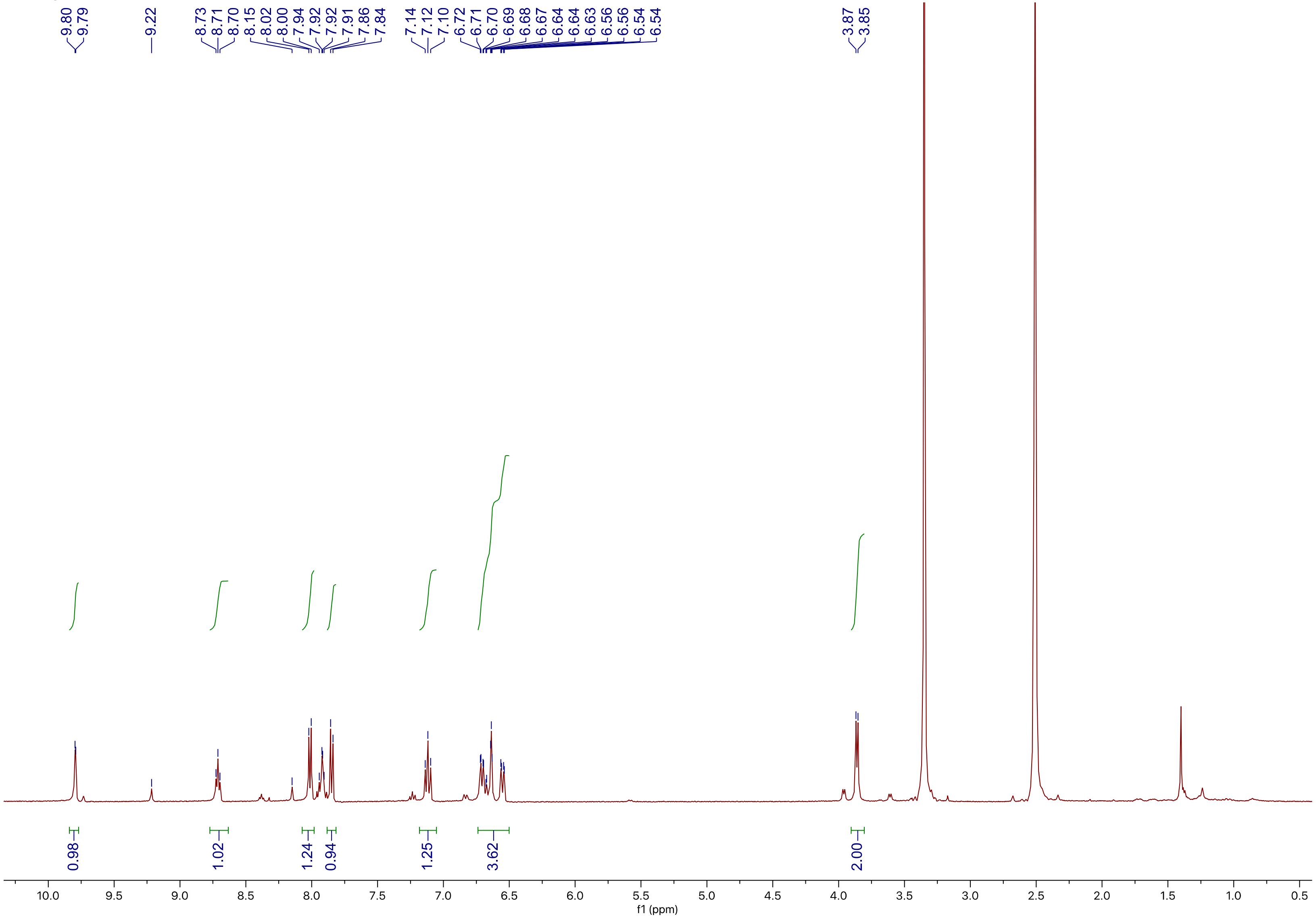

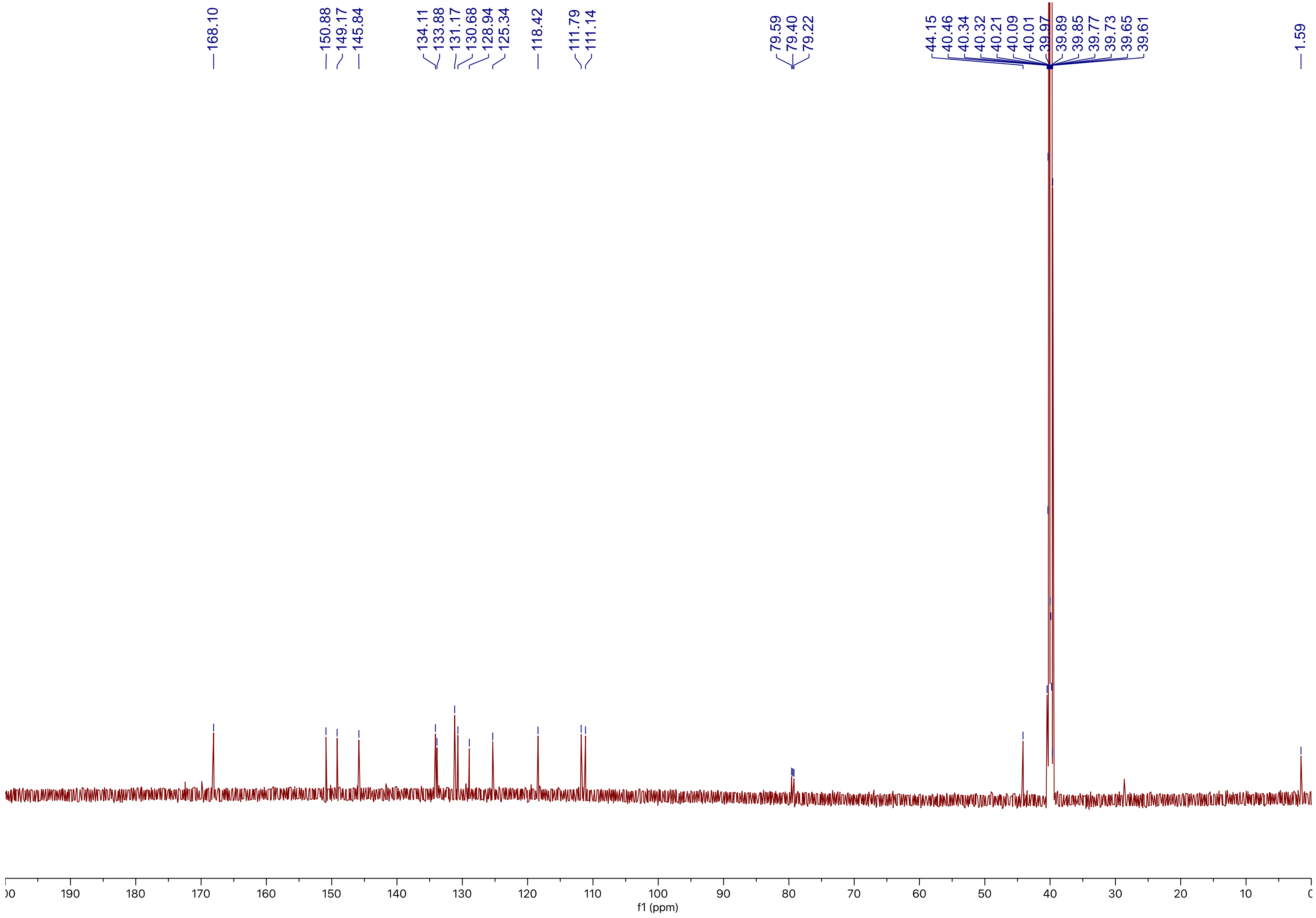
168.10
150.88
149.17
145.84
134.11
133.88
131.17
130.68
128.94
125.34
118.42
111.79
111.14
79.59
79.40
79.22
44.15
40.46
40.34
40.32
40.21
40.09
40.01
39.97
39.89
39.85
39.77
39.73
39.65
39.61
1.59
190
180
170
160
150
140
130
120
110
100
90
80
70
60
50
40
30
20
10
f1 (ppm)

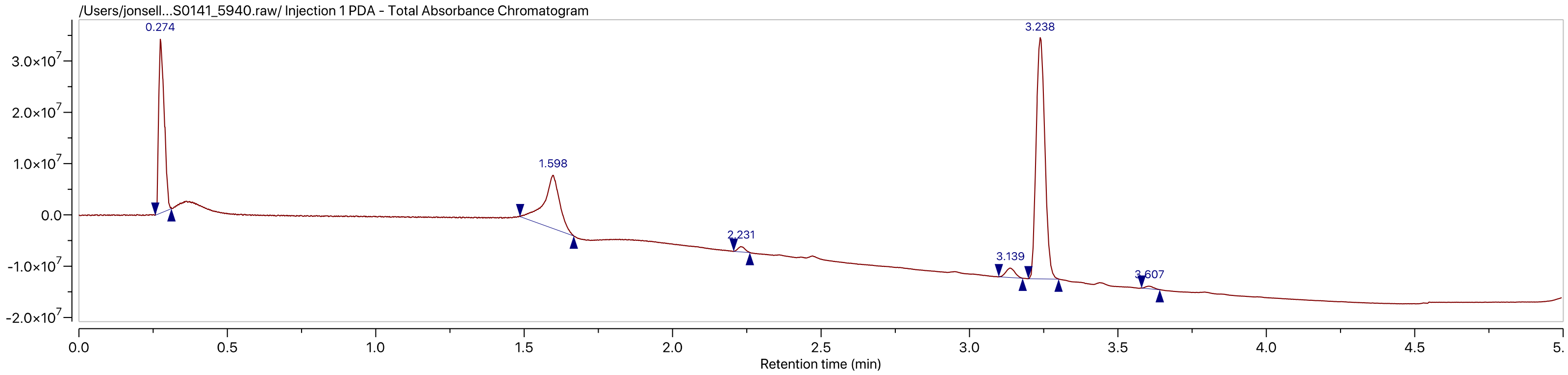
/Users/jonsell...S0141_5940.raw/ Injection 1 PDA - Total Absorbance Chromatogram
0.274
1.598
2.231
3.139
3.238
3.607
Retention time (min)

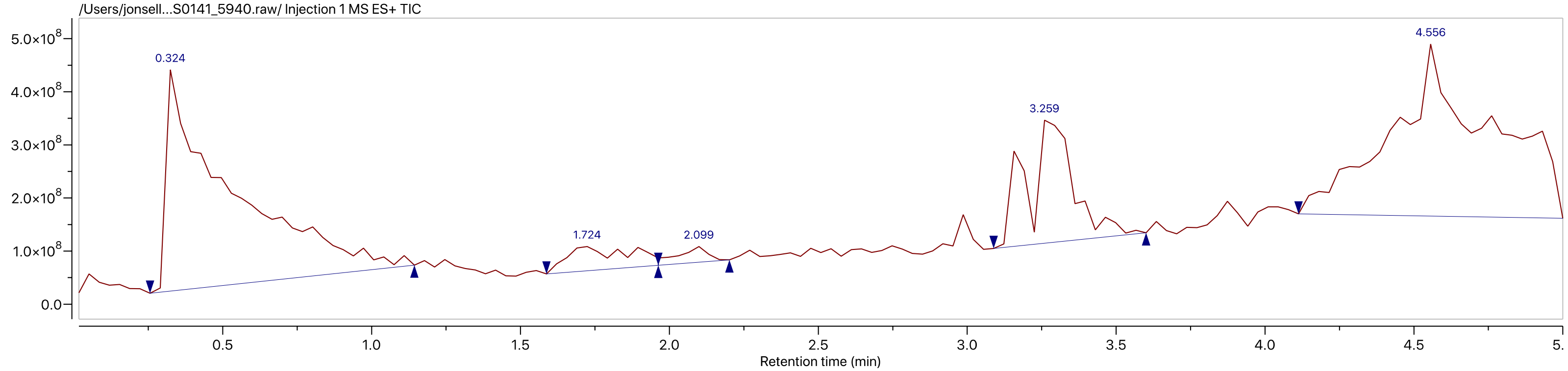
/Users/jonsell...S0141_5940.raw/ Injection 1 MS ES+ TIC
0.324
1.724
2.099
3.259
4.556
Retention time (min)

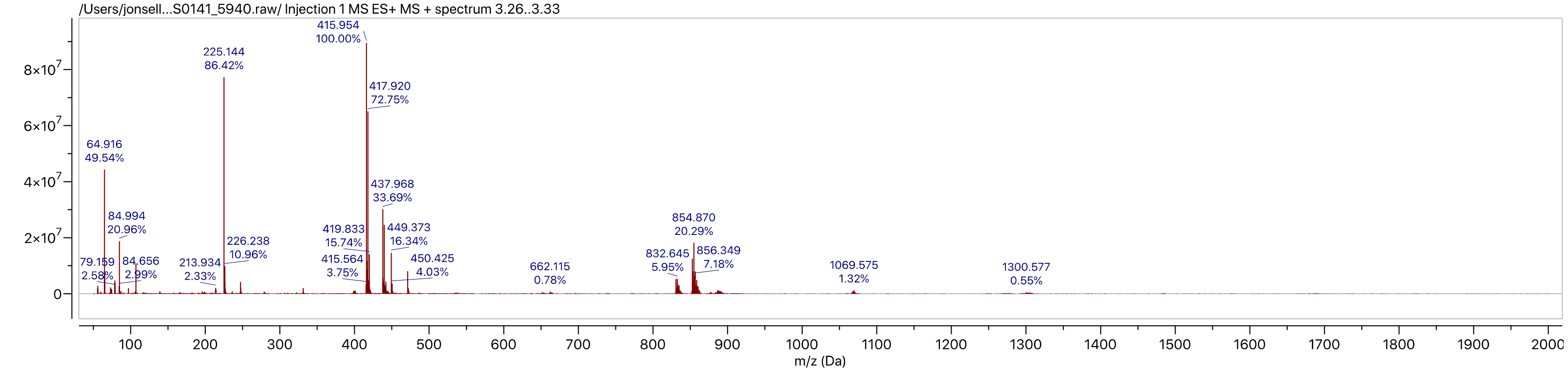
/Users/jonsell...S0141_5940.raw/ Injection 1 MS ES+ MS + spectrum 3.26..3.33
415.954 100.00%
225.144 86.42%
417.920 72.75%
64.916 49.54%
437.968 33.69%
84.994 20.96%
854.870 20.29%
419.833 15.74%
449.373 16.34%
226.238 10.96%
79.159 2.58%
84.656 2.99%
213.934 2.33%
415.564 3.75%
450.425 4.03%
662.115 0.78%
832.645 5.95%
856.349 7.18%
1069.575 1.32%
1300.577 0.55%
m/z (Da)

Compound 22a

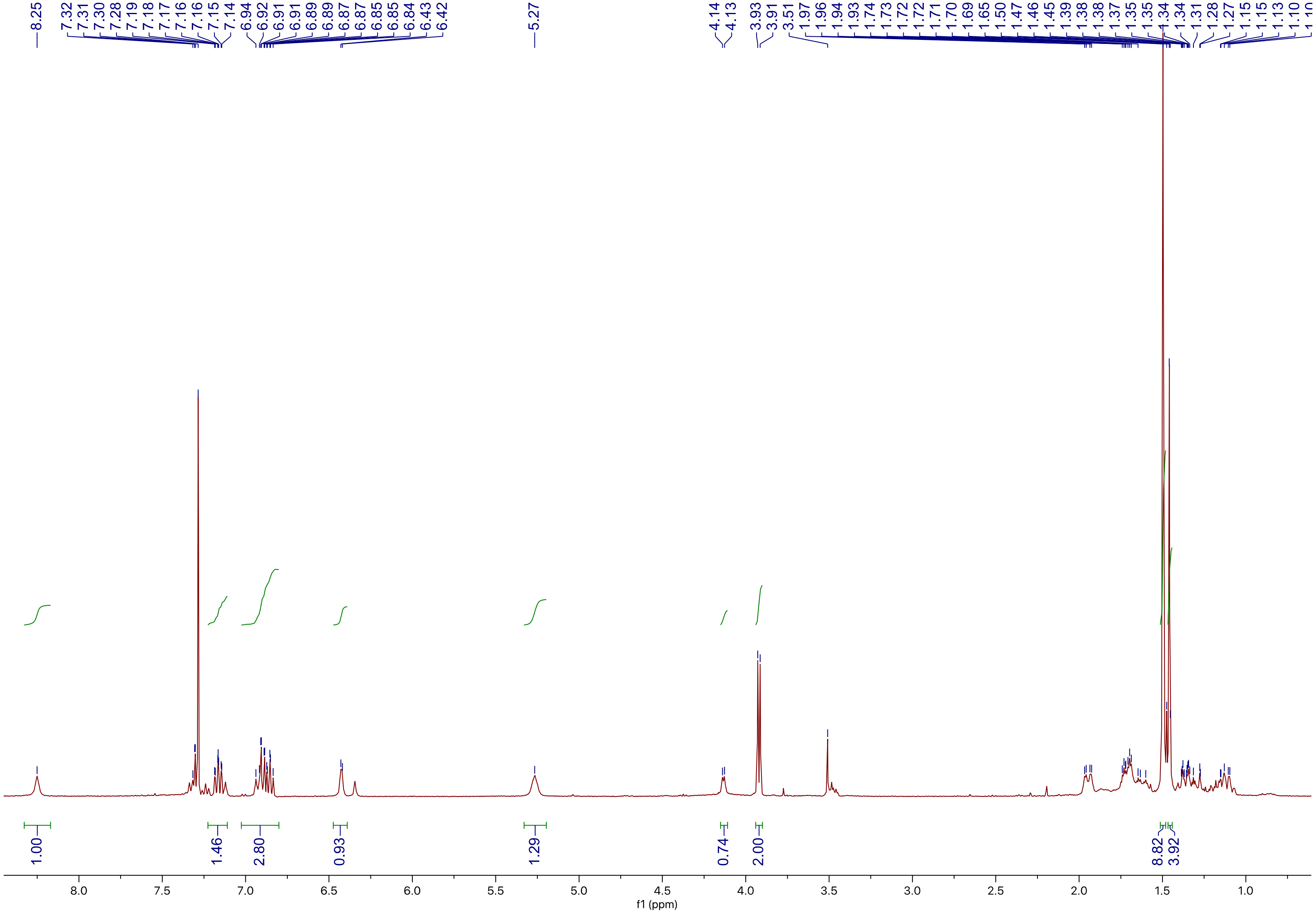

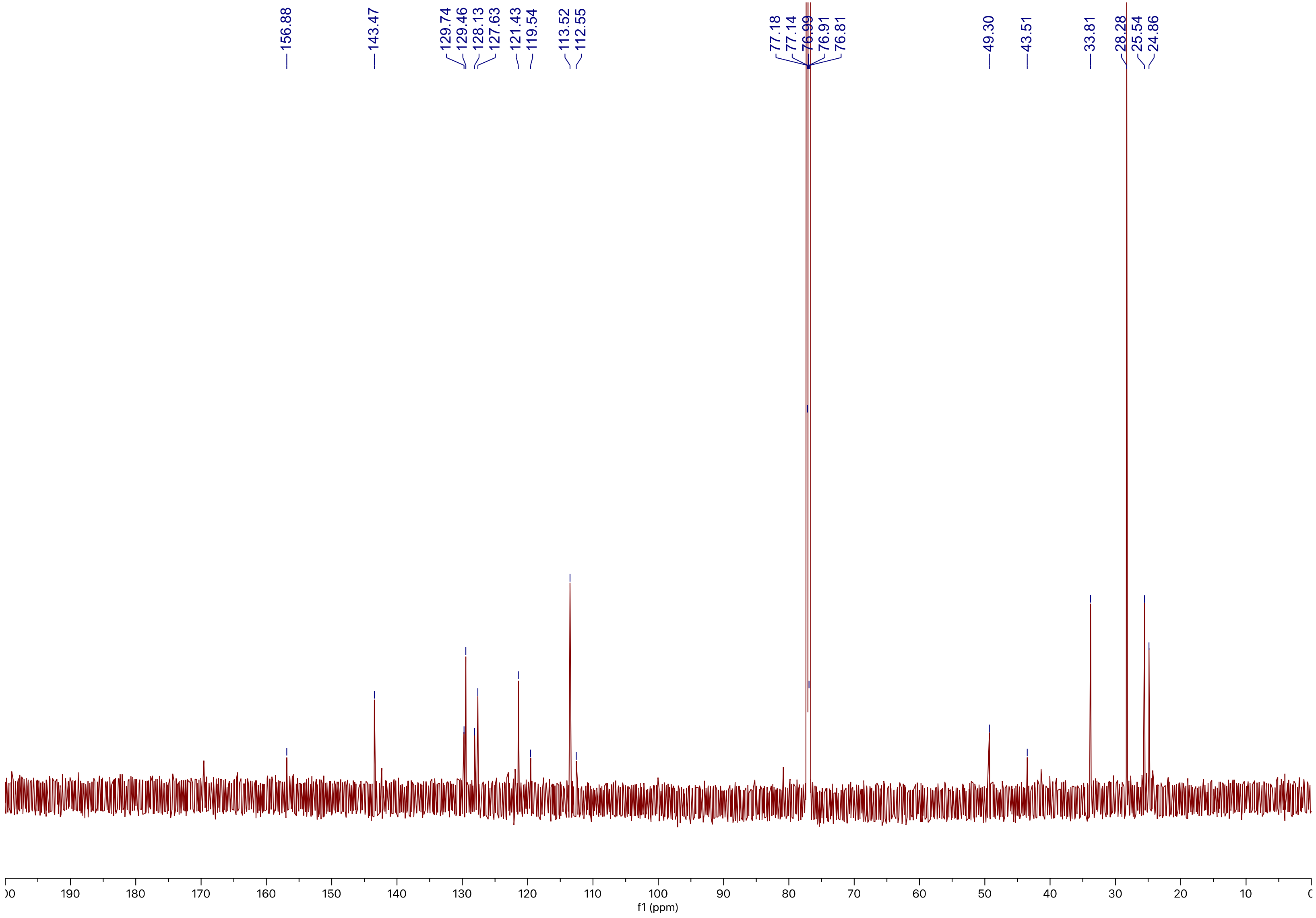
156.88
143.47
129.74
129.46
128.13
127.63
121.43
119.54
113.52
112.55
77.18
77.14
76.99
76.91
76.81
49.30
43.51
33.81
28.28
25.54
24.86
f1 (ppm)

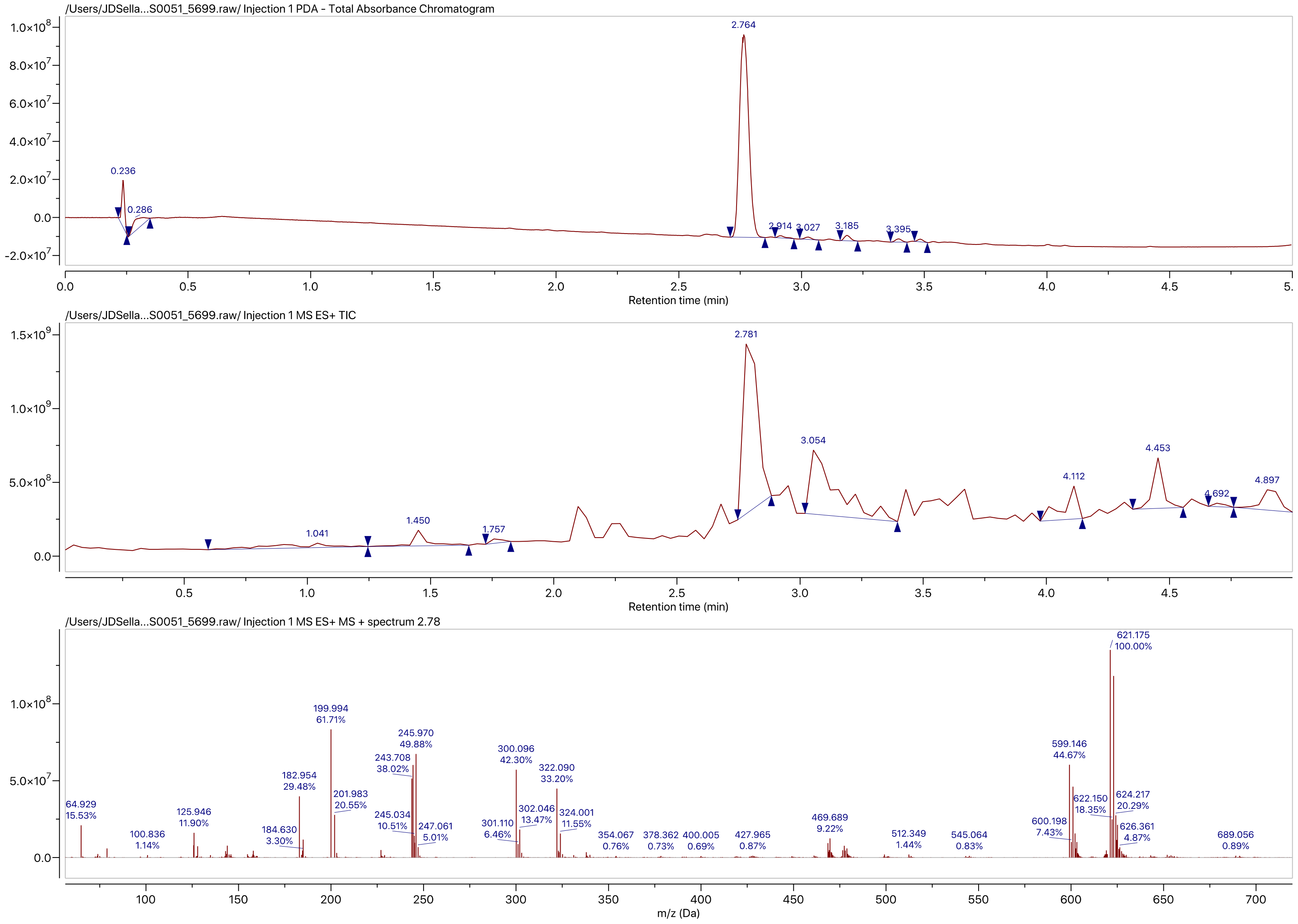
/Users/JDSella...S0051_5699.raw/ Injection 1 PDA - Total Absorbance Chromatogram
0.236
0.286
2.764
2.914
3.027
3.185
3.395
Retention time (min)
/Users/JDSella...S0051_5699.raw/ Injection 1 MS ES+ TIC
1.041
1.450
1.757
2.781
3.054
4.112
4.453
4.692
4.897
Retention time (min)
/Users/JDSella...S0051_5699.raw/ Injection 1 MS ES+ MS + spectrum 2.78
64.929 15.53%
100.836 1.14%
125.946 11.90%
182.954 29.48%
184.630 3.30%
199.994 61.71%
201.983 20.55%
243.708 38.02%
245.034 10.51%
245.970 49.88%
247.061 5.01%
300.096 42.30%
301.110 6.46%
302.046 13.47%
322.090 33.20%
324.001 11.55%
354.067 0.76%
378.362 0.73%
400.005 0.69%
427.965 0.87%
469.689 9.22%
512.349 1.44%
545.064 0.83%
599.146 44.67%
600.198 7.43%
621.175 100.00%
622.150 18.35%
624.217 20.29%
626.361 4.87%
689.056 0.89%
m/z (Da)

Compound 22b

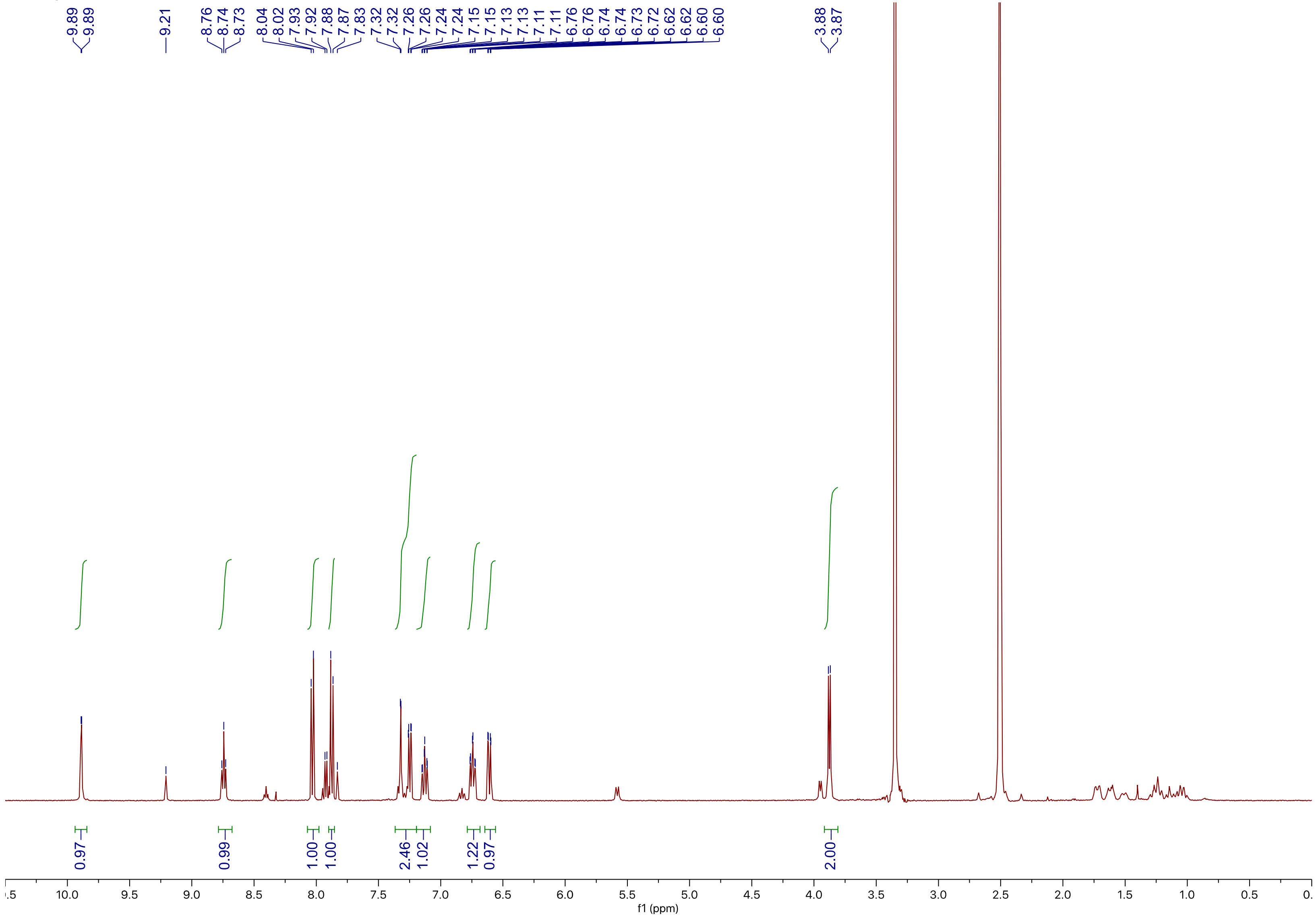

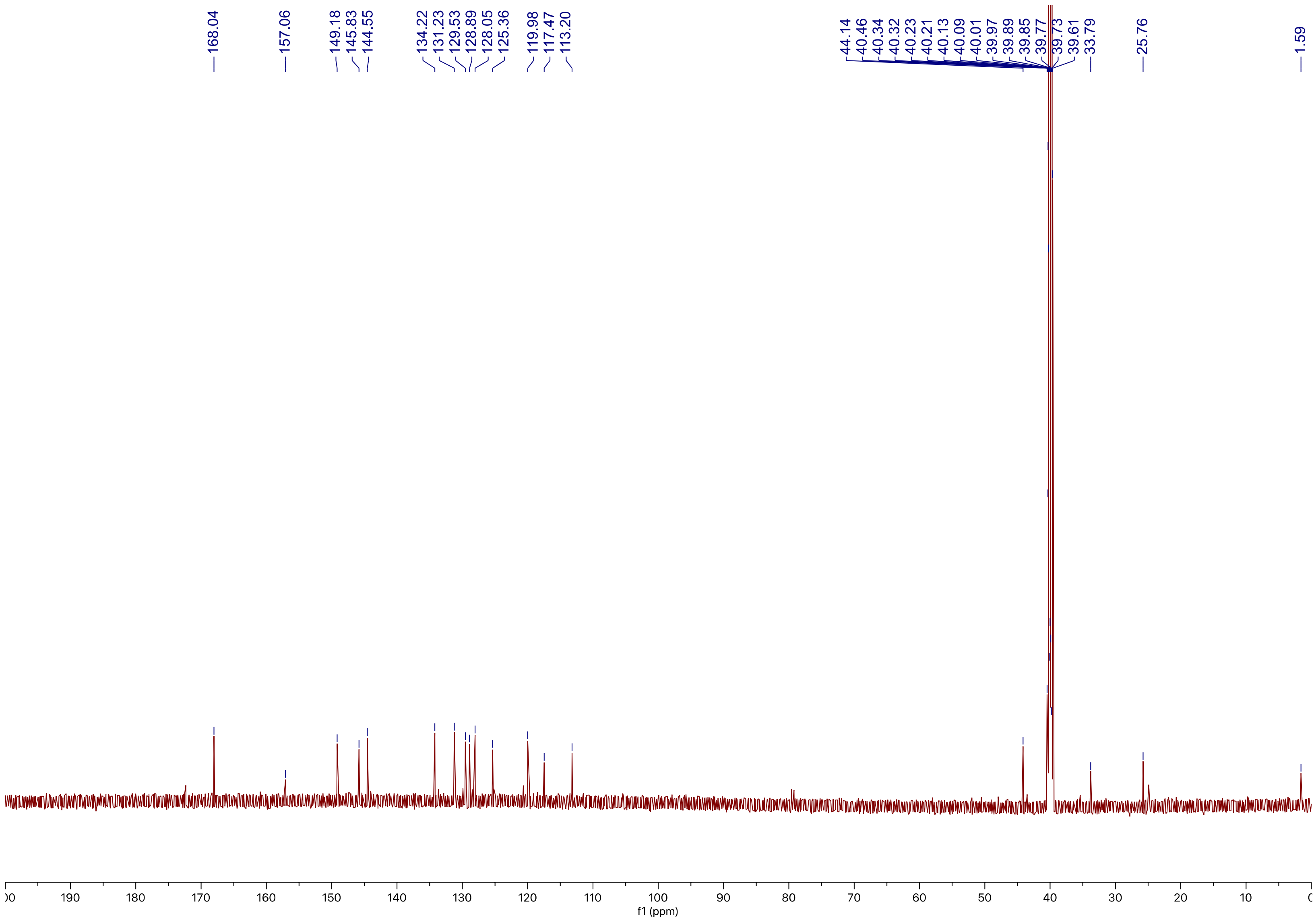
168.04
157.06
149.18
145.83
144.55
134.22
131.23
129.53
128.89
128.05
125.36
119.98
117.47
113.20
44.14
40.46
40.34
40.32
40.23
40.21
40.13
40.09
40.01
39.97
39.89
39.85
39.77
39.73
39.61
33.79
25.76
1.59
f1 (ppm)

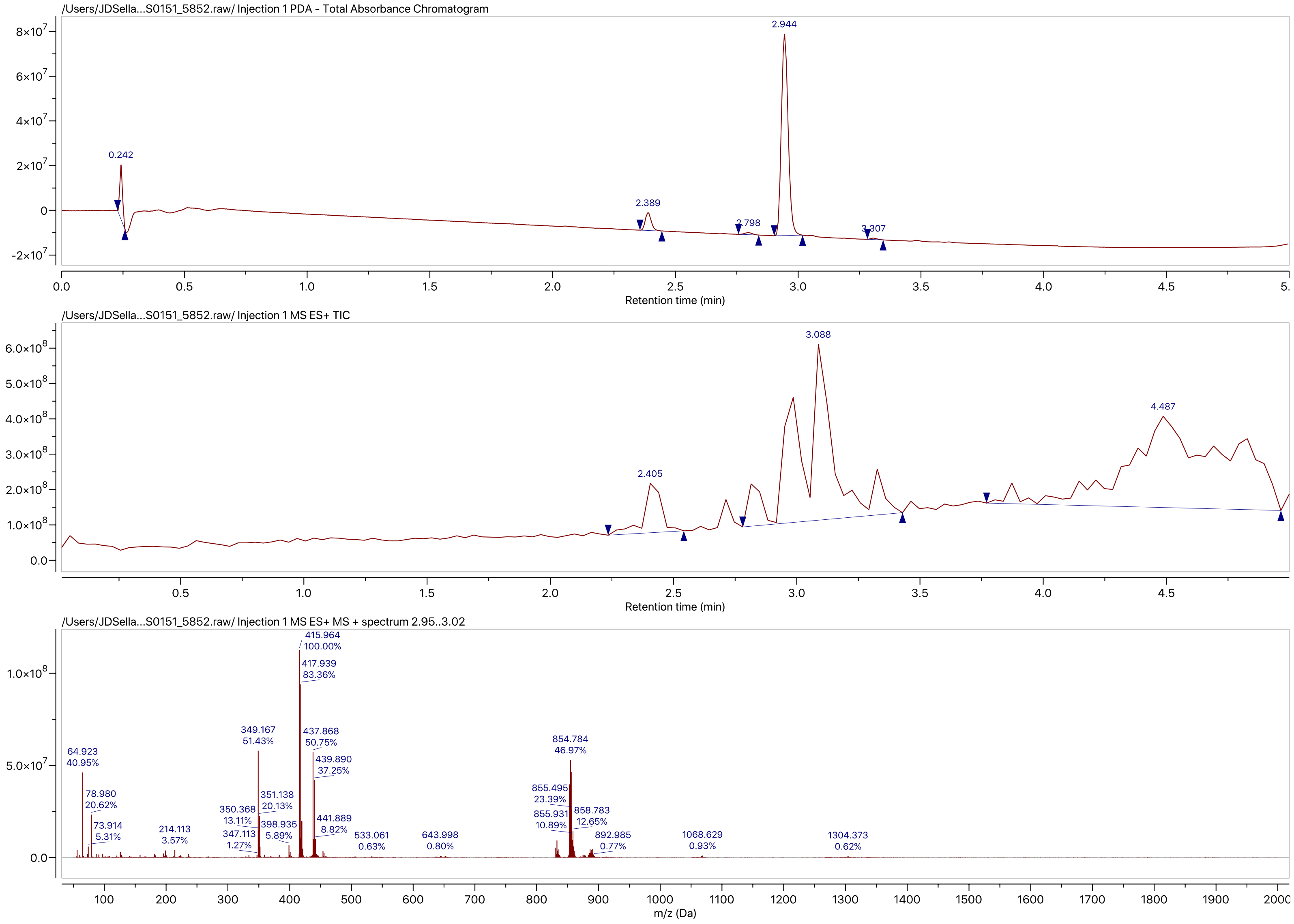

/Users/JDSella...S0151_5852.raw/ Injection 1 PDA - Total Absorbance Chromatogram
0.242
2.389
2.798
2.944
3.307
Retention time (min)
/Users/JDSella...S0151_5852.raw/ Injection 1 MS ES+ TIC
2.405
3.088
4.487
Retention time (min)
/Users/JDSella...S0151_5852.raw/ Injection 1 MS ES+ MS + spectrum 2.95..3.02
415.964 100.00%
417.939 83.36%
349.167 51.43%
437.868 50.75%
854.784 46.97%
64.923 40.95%
439.890 37.25%
855.495 23.39%
78.980 20.62%
351.138 20.13%
350.368 13.11%
858.783 12.65%
855.931 10.89%
441.889 8.82%
398.935 5.89%
73.914 5.31%
214.113 3.57%
347.113 1.27%
1068.629 0.93%
643.998 0.80%
892.985 0.77%
533.061 0.63%
1304.373 0.62%
m/z (Da)
